# Abstracts from the 6th International Scientific Conference on Exercise and Quality of Life

**DOI:** 10.1186/s12919-024-00297-y

**Published:** 2024-07-19

## S1 Effects of resistance training on sleep quality and disorders among individual diagnosed with cancer: an overview within the OACCUS project

### Antonino Bianco^1,2^

#### ^1^Sport and Exercise Sciences Research Unit, Department of Psychology, Educational Science and Human Movement, University of Palermo, Palermo, Italy; ^2^Outdoor Against Cancer Connects Us; OACCUS EU-Health project

##### **Correspondence:** Antonino Bianco (antonino.bianco@unipa.it)

*BMC Proceedings 2024*, **18(11)**: S1

**Abstract**

A healthy lifestyle appears to be difficult to achieve for cancer patients after the end of therapies, and this may cause different side effects and physiological and psychological impairments. The preliminary findings from the OACCUS team pointed out that not only the healthy diet and the non-pharmacological strategies such as exercise interventions but also sleep disorders and mental health require attention and a deeper investigation, particularly in Young Cancer Survivors (YCS). Accordingly, I'll explore the scientific evidence already published within the OACCUS project. In more detail, I'll mention our interesting systematic review of the potential novel effect of RT on sleep outcomes in cancer survivors.

The overall methodological rationale adopted by the consortium will be shared with the audience, particularly all preliminary actions required for the research lines definitions. Regarding the literature review, the search was conducted on MEDLINE (Pubmed), Web of Science, Scopus, and Cochrane Central Register of Controlled Trials databases, including only randomized controlled trials (RCTs). The screening procedure was conducted using the web-based software COVIDENCE. The existing review protocol investigating on sleep outcomes in cancer survivors has been registered in the PROSPERO database [CRD42023426762].

Gentile et al. investigated the effect of lack of social support in physical activity practice on quality of life and mental health in YCS in 2023 (PMID 37837211) and 2024 (DOI:10.1002/casp.2786). On the other hand, Lakicevic et al. in 2023 (DOI:10.3389/fonc.2023.1284052) highlighted the importance of resistance training in young cancer survivors. Concerning the sleep disorders review, more than 20 studies were included in the analysis (taken from 11,663 studies identified during the first-round search).

The OACCUS project is about fostering a future-oriented healthy lifestyle through four core elements: a) outdoor sports and exercise, b) psychoeducation, c) healthy nutrition and d) a healthy natural environment. Resistance training exercise interventions performed weekly, with exercise intensity ranging from 60 to 80% of one-repetition maximum can be administered to cancer survivors, aiming to improve sleep outcomes.

## S2 Sports cardiology

### Aleksandra Milovančev^1,2^

#### ^1^Faculty of Medicine, University of Novi Sad, Novi Sad, Serbia; ^2^Institute of Cardiovascular Diseases of Vojvodina, Sremska Kamenica, Serbia

##### **Correspondence:** Aleksandra Milovančev (aleksandra.milovancev@mf.uns.ac.rs)

*BMC Proceedings 2024*, **18(11)**: S2

**Abstract**

A sedentary lifestyle has led to an increase in obesity, high blood pressure, and type 2 diabetes. Exercise is promoted to mitigate these risks, with exercise intensity considered the most critical element. Although physical activity and aerobic capacity are believed to protect against various morbidities through multiple pathways few people in developed countries are sufficiently physically active to derive health benefits. Recommendations call for engagement in moderate physical activity for about 30 minutes each day for most days. An athlete’s heart is a normal, physiological adaptation of the body to the intensive workout and aerobic exercise. There is no clear boundary on when physiological adaptation, which can be extreme in highly trained athletes (especially endurance athletes), starts contributing to arrhythmogenesis or becomes pathologic. Excessive training stimulus without adequate recovery poses the risk of cumulative microscopic injury and right ventricular structural and functional remodeling, while ideal training stimulus with recovery causes physiological adaptation with balanced hypertrophy and little or no fibrosis of the athlete's heart. Increased biomarker levels of cardiac injury are reported after extreme endurance sports activities. Case reports of adverse events have been reported in specific situations like rapid weight loss. Additional screening, follow up, and assessment is needed to preserve athlete's health and safety and avoid catastrophic events.

Further research is required to distinguish between changes in biomarkers caused by normal physiological processes and those that indicate pathologies. We need to understand the mechanisms underlying the release of biomarkers during exercise and determine whether post-exercise biomarker levels can be used as a novel indicator of cardiovascular risk. Although regular physical activity provides numerous health benefits, intense exercise can paradoxically trigger life-threatening ventricular arrhythmias and myocardial injury. Therefore, ensuring cardiovascular safety during sports participation for individuals of all ages is crucial to prevent often avoidable adverse events

## S3 Bridging the gap: Translating biomechanics research from lab to field for practical impact and collaborative innovation

### Nachiappan Chokalingam

#### School of Health, Science and Wellbeing, Staffordshire University, UK

##### **Correspondence:** Nachiappan Chokalingam (n.chockalingam@staffs.ac.uk)

*BMC Proceedings 2024*, **18(11)**: S3

**Abstract**

Biomechanics research holds a huge potential to improve human health, performance, and quality of life. However, the path from academic labs to real-world applications is often met with challenges. To maximize the impact of their work, practitioners and scientists must establish strong collaborative partnerships. A key obstacle is the inherent differences between the controlled conditions of the lab and the complexities of the field. Laboratory studies allow for precise data collection and rigorous analysis, but may not fully capture the dynamic, multifaceted nature of real-world activities and environments.

Practitioners, who intimately understand the needs and constraints of their domains, can provide crucial insights to help researchers design studies that better reflect on-the-ground realities. Similarly, scientists possess specialized expertise in biomechanical principles, novel technologies, measurement techniques, and data interpretation that can greatly enhance the practical applications developed by practitioners. By working together, they can iterate on prototypes, validate findings, and refine solutions to ensure they are feasible, effective, and user-friendly.

Another crucial element is the funding of this translation. A key to any successful translational research is appropriate stakeholder interaction and funding. These stakeholders essentially form a part of the wider collaborative team. These teams must also navigate organizational, regulatory, and commercial hurdles that can impede the translation of research into marketable products or clinical interventions. Scientists bring technical credibility, while practitioners offer crucial knowledge of industry standards, consumer preferences, and healthcare systems. Merging these perspectives is essential for steering innovations through the complex pathways to real-world implementation.

Ultimately, the most promising biomechanics breakthroughs will emerge from a spirit of mutual respect, open communication, and a shared commitment to improving human lives. By bridging the lab-to-field divide through strategic partnerships, we can maximize its potential to transform how we move, function, and thrive.

## S4 Exploring the potential physiological effects of molecular hydrogen on exercise performance and adaptations

### Tyler W. LeBaron^1,2^

#### ^1^Department of Kinesiology and Outdoor Recreation, Southern Utah University, Cedar City, United States of America; ^2^Molecular Hydrogen Institute, Southern Utah University, United States of America

##### **Correspondence:** Tyler W. LeBaron (tylerlebaron@suu.edu)

*BMC Proceedings 2024*, **18(11)**: S4

**Abstract**

Exercise is known to confer numerous health benefits, yet it can also induce physiological stressors such as inflammation and oxidative damage, particularly during high-intensity or noxious exercise. Molecular hydrogen (H2) has emerged as a promising therapeutic agent due to its antioxidant and anti-inflammatory properties. This raises the possibility of potential synergistic effects of H2 administration and exercise on physiological adaptations. Current research indicates that exercise-induced pro-inflammatory cytokines and reactive oxygen species (ROS) play a crucial role in mediating the beneficial effects of exercise, including cytoprotective hormesis and mitochondrial biogenesis. However, excessive inflammation and oxidative stress can lead to redox dysregulation and chronic inflammation, impairing exercise-induced adaptations. Nevertheless, ingestion of conventional antioxidants and anti-inflammatories may blunt the beneficial effects of exercise by interfering with ROS signaling pathways. In contrast, H2 administration has been shown to mitigate redox dysregulation and inflammation associated with exercise, potentially enhancing exercise-induced adaptations. Intriguingly, H2 may act as an exercise mimetic and redox adaptogen. By potentiating the benefits of beneficial exercise and reducing the harm from noxious exercise, H2 administration could offer novel strategies for optimizing exercise performance and promoting overall health. While preliminary evidence suggests the potential ergogenic and therapeutic effects of H2 in exercise medicine, further research is needed to elucidate its mechanisms of action and optimize its application in athletic performance and clinical settings. This exploration of the interplay between molecular hydrogen and exercise holds promise for enhancing our understanding of exercise physiology and developing innovative approaches to optimize human performance and health.

## S5 When the kids stood still: The effects of COVID-19 mitigation measures on children’s development

### Gregor Starc

#### Faculty of Sport, University of Ljubljana, Slovenia

##### **Correspondence:** Gregor Starc (gregor.starc@fsp.uni-lj.si)

*BMC Proceedings 2024*, **18(11)**: S5

**Abstract**

In 2020 and 2021 the global COVID-19 pandemic profoundly affected children’s everyday life and the introduced mitigation measures diminished their access to physical activity.

The somatic and motor development data from the Slovenian national surveillance system SLOfit was used to analyze the changes in children. Cohort and cross-sectional data were used for comparison of developmental trends between pre-COVID and COVID-affected generations of children. In addition, the ACDSi study data from 2013 and 2023 was used to observe the trends in physical activity and sedentariness among children.

Between 2013 and 2023 the sedentary time of children in Slovenia after school increased from 75 minutes to 305 minutes per day, while their moderate to vigorous daily physical activity decreased from 140 to 130 minutes. Consequently, physical fitness of generations, affected by COVID-related mitigation measures, decreased up to 19%. The decreased physical activity during the pandemic was linked also to accelerated growth of children, who grained 8mm more height than their peers from the pre-pandemic period.

The generations of children, affected by the COVID-19 mitigation measures are facing increased health risks and reduced work capacity in comparison to their pre-pandemic peers. Due to lack of reliable population data in most of the countries, the decision makers are unaware of the long-term consequences and so far, failed to introduce any policies that would provide more opportunity for physical activity of affected children and youth.

## S6 A complex, health-economic and public health study of the effects of physical activity

### Pongrác Ács

#### Faculty of Health Sciences, Institute of Physiotherapy and Sport Science, University of Pécs, Pécs, Hungary

##### **Correspondence:** Pongrác Ács (dekan@etk.pte.hu)

*BMC Proceedings 2024*, **18(11)**: S6

**Abstract**

In Europe, physical inactivity is responsible for 600,000 deaths and 5.3 million of healthy life years lost each year. According to the 2022 Eurobarometer data, 59% of Hungarian society is still inactive. Therefore, broadening our target population, in addition to certified athletes (4.5% in 2017, approximately 450,000 people), we also target the general population who report themselves as physically active or inactive.

Our institution provides sports and health-related services in the fields of dietetics, physiotherapy, sports programs, prevention and rehabilitation within the framework of the entrepreneur university concept: beyond university citizens, it offers diagnostic and performance development opportunities for athletes, for diaspora Hungarians beyond the region through our cross-border training, and provides links to companies and industrial investors (analytical testing, functional food development).

The complex examination of the effects of physical activity in the field of health preservation, health promotion and rehabilitation will be presented. Athletes and the general population are assessed similarly with different protocols, and based on the measurements, we provide dietary and exercise management advice. In our population-based studies, questionnaires on physical activity are supplemented with objective measurements (anthropometry, body composition, exercise physiology, 3D full body scanning, accelerometry).

In addition to health-related aspects, we also investigate empirical studies of the territorial competitiveness of sport, national economic burden of physical inactivity, public sport consumption patterns, the economic and social effects of sport events, return on investment of sports facilities, sport portfolio matrix.

Our team, operating in a research and innovation model within the higher education ecosystem, has a number of innovations and developments, public health maintenance apps, full body scanning, instrumental and questionnaire measures of stress and anxiety, all aimed at improving athletes’ performance, health and reducing inactivity.

This research was sponsored by the National Research, Development and Innovation Fund of Hungary (TKP-2021-EGA-10).

## S7 “Personalized Recovery” – The future of recovery

### Julio Calleja Gonzalez

#### University of the Basque Country (UPV/EHU), Spain

##### **Correspondence:** Julio Calleja Gonzalez (julio.calleja.gonzalez@gmail.com)

*BMC Proceedings 2024*, **18(11)**: S7

**Abstract**

Recovery is a trending topic in the field of sports science, and even more so in the world of elite sport. This is evidenced by the significant increase in scientific publications during the last 10 years as teams look to find a competitive edge during competitions.

Different protocols describe specific recovery methodologies that can be employed in order to achieve more efficient recovery processes, these include (1) recovery strategies foam roll, massage, compression garments, stretching, nutrition, active recovery, sleep, water immersion, (2) combinations of recovery strategies, (3) sport-specific recovery characteristics - soccer, basketball, volleyball, rugby, and combat sports, and (4) emerging recovery strategies. Additionally, factors, such as recovery time-periods - post-match and during congested schedules, have also been investigated, with emerging literature examining recovery specific to female athletes and youth athletes.

However, the future goal is to optimize an athlete's recovery and overall athletic performance by tailoring recovery strategies to their individual/personal needs and techniques that can assist in developing effective individualized recovery strategies. The importance of assessing an athlete's specific recovery needs, selecting appropriate recovery techniques, and monitoring outcomes to develop personalized recovery strategies.

Therefore, Team staff members and athletes should adopt a combination of approaches, such as the time-based and individualized approaches, to create effective recovery strategies that can be adjusted as needed to ensure peak performance and overall well-being. [28]. In consequence, future customization on recovery should address related issues such as: response to the recovery process, being a good or bad responder, differences between members, preferences, anatomical variability, intolerances, as well as intra-individual variability.

## S8 Beyond the ring - Understanding self-declared prohibited substance use in combat sports: A Serbian perspective

### Ivana M. Milovanović, Vuk Raonić, Roberto Roklicer, Marko Manojlović, Tatjana Trivić, and Patrik Drid

#### Faculty of Sport and Physical Education, University of Novi Sad, Novi Sad, Serbia

##### **Correspondence:** Ivana M. Milovanović (ivana.milovanovic@fsfvns.edu.rs)

*BMC Proceedings 2024*, **18(11)**: S8

**Abstract**

Social science research on doping practices in sports primarily depends on self-reports. Research on the use of prohibited substances suggests that self-reporting tends to involve under-reporting. Similarly, doping practices are likely to be under-reported, with potentially increasing occurrence.

This exploratory research was carried out using a semi-structured interview with 30 athletes (male) from combat sports. The common characteristic of all interviewees is self-reported prohibited substance use (to the researcher), at least in some phase of sports career. The main goal of the research was to determine reasons for the doping use in combat sports, as well as, duration of use, type of used substances, and the self-evaluated consequences after the substance abuse.

Most of the interviewees learned about doping from training peers or more experienced sportsmen, from parents who were active in sports, internet sources; noticeably less often from coaches. The interviewees confirmed positive experiences with doping in combat sports. They point out that quick recovery is crucial for more training and emphasize the positive impact of doping on their performance. Some interviewees claim that the use of doping is necessary to achieve top results, and possible side effects after stopping use include pain in the bones and muscles.

Doping fundamentally remains an intractable issue in modern sports and occurs in a complex environment that includes interactions between individual and environmental factors. This exploratory field research carried out with representatives of combat sports, confirm this claim. At the same time, they testify to the necessity of "demystification" of the use of doping and systemic primary prevention among (younger) athletes.

## S9 Sport & Sustainability: A paradox, a challenge, an opportunity

### Attilio N. Carraro

#### Faculty of Education, Free University of Bozen-Bolzano, Italy

##### **Correspondence:** Attilio N. Carraro (attilio.carraro@unibz.it)

*BMC Proceedings 2024*, **18(11)**: S9

**Abstract**

Sustainability is defined as the ability to uphold improved and desirable conditions in the long term, considering both intergenerational and intragenerational perspectives. A three-pillar conception of sustainability (social, economic and environmental), with diverse possibilities for connections between the pillars has been proposed (Purvis, Mao, & Robinson, 2019). Starting from this view, the sport and sustainable development concept considers sport as a potential powerful vehicle for promoting sustainability, identifying six key dimensions: personal, social, economic, ecological, technological, and political. Each dimension sets specific objectives that guide decision-making, encouraging people and stakeholders to prioritize sustainable development within the realm of sport.

The aim of this presentation is to introduce the cons (paradoxes), the pros (opportunities), and the challenges (research and education) in the sport & sustainability relationship, providing insights to respond to two questions: “Is sport sustainable?” and “How can sport educate to sustainability?”.

A narrative review has been conducted to present elements which can help to provide answers to the question posed above.

The United Nations recognizes the power of sport in achieving the Sustainable Development Goals. Sport is universally acknowledged for promoting health and well-being (Goal 3) and providing a means to foster inclusive and quality education and lifelong learning (Goal 4). Additionally, sport can serve as a driving force in promoting gender equality (Goal 5), and reducing social inequalities (Goal 10). However, major sporting organizations often fall short in practice, with their high carbon footprint standing as a testament to their departure from true sustainability. Moreover, elite sports, as they currently operate, don't serve as a model, and even the individual practice of sports seems often not-sustainable when viewed from a holistic perspective.

While there are evident incongruences within the sport system, the potential of sport to significantly contribute to global sustainable development remains undeniable.

## S10 Exceptional body height of people from the Karst Area of the Dinaric Mountain Range: A retrospective of previous knowledge and perspective of advanced assessment methods

### Stevo Popović^1,2^, Miodrag Zarubica^3^

#### ^1^Balkan Institute of Science and Innovation, University of Montenegro, Podgorica, Montenegro; ^2^Faculty for Sport and Physical Education, University of Montenegro, Nikšić, Montenegro; ^3^Faculty of Electrical Engineering, University of Montenegro, Podgorica, Montenegro

##### **Correspondence:** Stevo Popović (stevop@ucg.ac.me)

*BMC Proceedings 2024*, **18(11)**: S10

**Abstract**

The exceptional body height of people from the karst area of the Dinaric Mountain range has attracted the attention of leading anthropologists since the end of the 19th century, mainly because the inhabitants of the Dinaric Alps have long been known as people of impressive stature. However, in the last century, there was a critical lack of systematic monitoring of this phenomenon and, therefore the purpose of this presentation was to collect all prior studies and make the most suited retrospective of knowledge, but also to offer a suitable perspective of advanced assessment methods.

A comprehensive synthesis of recent studies has led to the fact that the inhabitants of the Dinaric Alps are some of the tallest people in the world. From the reason body height is important in many settings such as giving an assessment of nutritional status and a meaningful measure of determination of basic energy requirements, standardization of measures of physical capacity and adjusting drug dosage, evaluation of children’s growth, prediction and standardization of physiological variables as well as talent identification and success in sports, this study precisely summarized all the specific socioeconomic, nutritional, and genetic characteristics of people from this mountain range. Hence, it offered possible explanations for this anthropological phenomenon as well as advanced assessment methods, all to better ensure longevity, a lower risk of adverse pregnancy outcomes and cardiovascular and respiratory diseases, but also to decrease a higher risk of some cancers that are strongly correlated with stature, et cetera.

Obtained knowledge in exceptional body height of people from the Dinaric Alps presents valid guidelines for further investigations, with a growing emphasis on providing advanced assessment methods such as machine learning that would simplify measuring body height and reduce the cost of data collection in the future.

## S11 FISU's multifaceted approach to student well-being, urban development, and sustainability: A comprehensive analysis

### Andrea Uccello

#### The International University Sports Federation

##### **Correspondence:** Andrea Uccello (a.uccello@fisu.net)

*BMC Proceedings 2024*, **18(11)**: S11

**Abstract**

The International University Sports Federation (FISU), established in 1949, is the preeminent global governing body for national university sports organizations. Beyond its primary role of orchestrating sports competitions for student-athletes worldwide, FISU embarks on a broader mission encompassing various domains. This research explores FISU's multifaceted approach, highlighting its endeavors to promote student well-being, nurture urban development, and champion sustainability.

This research delves into FISU's flagship events, such as the World University Games (Universiade), World University Championships, and University World Cups, unveiling their profound impact on city development and the cultivation of youth citizenship. Additionally, it delves into FISU's educational initiatives, accentuating its efforts in facilitating dual career paths and advocating for healthy lifestyles among students. Furthermore, the research illuminates FISU's commitment to environmental stewardship through its sustainability projects to mitigate ecological footprints associated with sporting events.

Through a holistic analysis of FISU's endeavors, this research underscores the organization's pivotal role in sculpting a brighter, healthier future for students globally while fostering sustainable development on a grand scale.

## S12 Understanding the Ski Easy unified teaching model tools. How and why is easy?

### Saša Pišot^1^, Uroš Marušič^1,2^, Rado Pišot^1^

#### ^1^Science and Research Center Koper, Institute for Kinesiology Research, Koper, Slovenia; ^2^Department of Health Sciences, Alma Mater Europaea – ECM, Maribor, Slovenia

##### **Correspondence:** Saša Pišot (sasa.pisot@zrs-kp.si)

*BMC Proceedings 2024*, **18(11)**: S12

**Abstract**

The landscape of alpine skiing has changed dramatically in recent years. With the rapid development of ski equipment, which increases enjoyment, we also face decrease of motivation for outdoor sports in general. In addition, the societal shift towards a sedentary lifestyle has increased snow sports participants with limited motor and functional abilities, contributing to more injuries. It is therefore necessary to adapt the methods of teaching skiing and the skills of the teachers to make guest experience better. To teach young children effectively, the use of games and the inclusion of visual aids is essential for better understanding and learning. In the Erasmus+ project SKIEASY, the nine project partners have developed a Unified Teaching Model (UTM) for ski instructors using a mobile application to improve, adapt and simplify teaching methods.

With the aim of creating user-friendly tools that simplify teaching, the development of the UTM tools consists of several scientific and didactic approaches: the definition of 5 stages of ski skills acquisition, kinematic screen analysis with creation of graphic materials; creation of basic terminology for five different communication areas and translations and IT development (static website, unified animations).

The SKIEASY MobApp with dictionaries in 15 languages with audio collection of words about ski equipment, body parts, ski environment, ski technique, didactic tools, useful phrases) and 13 video animations of a unified 5-step teaching model with additional paper pocket manuals in 8 languages and many other didactic materials available on the SKI EASY website can be free accessed as additional help for teaching skiing.

How and why is easy? Because UTM offers a structured and coherent approach where the results reflect the initials of the project: Educational/Accessible/Simple/Youthful. The SKI EASY tools benefit both teachers and students by promoting clarity, efficiency, and a broader understanding of the subject matter.

## S13 From podium to hospital: Understanding the female athlete triad

### Fatma Neşe Şahin

#### Faculty of Sport Sciences, Ankara University, Ankara, Türkiye

##### **Correspondence:** Fatma Neşe Şahin (nese.sahin@ankara.edu.tr)

*BMC Proceedings 2024*, **18(11)**: S13

**Abstract**

In the realm of sports, athletes are celebrated for their physical prowess, endurance, and dedication. However, behind the glitz and glamour of the podium, there lies a lesser-known but critically important issue: the Female Athlete Triad. This condition, characterized by a combination of energy deficiency with or without eating disorder, menstrual irregularities, and decreased bone density, can have serious implications for the health and well-being of female athletes.

The Female Athlete Triad represents a spectrum of interrelated health concerns that often go unrecognized and untreated. It typically begins with disordered eating patterns, as athletes, driven by the pressure to excel and maintain a certain physique, may engage in restrictive eating or extreme dieting practices. This can lead to energy deficiency, which not only impairs athletic performance but also disrupts hormonal balance. In addition, although there is no eating disorder, we also see inadequate energy intake involuntarily or even without awareness.

Female athletes in aesthetic sports, such as gymnastics, are found to be at the highest risk for eating disorders. Elite athletes are found to have a higher rate of eating disorders than in a female control group 20% vs. 9%. In judge’s sports, such as gymnastics, the prevalence of eating disorders is 13% compared to 3% in refereed sports. Sports that athletes typically begin in childhood, low self-esteem; family dysfunction, physical or sexual abuse, peer, family or cultural pressures to be thin and other traumatic life experiences caused by eating disorder

Because eating disorders disrupt the body's energy input-output balance, they affect not a single structure in the body, but the entire organism. Eating disorders disrupt the hormonal balance in women at the highest level, right from the central nervous system. Energy deficit causes the body to shut down production of several hormones necessary to make estrogen. The lack of estrogen, in turn, triggers amenorrhea. The lack of estrogen in an amenorrheic athlete, combined with the lack of calcium in many women’s diets, results in loss of bone density

The medical consequences of the Female Athlete Triad can be permanent. Recent research on osteoporosis suggests that bone density lost as a result of the triad’s disorders is never regained, meaning the victim will have weaker bones and an increased risk of fractures for the rest of her life. Low energy availability with or without eating disorders, hypothalamic amenorrhea, and low BMD alone or in combination, significant health risks to physically active girls and women.

Prevention, recognition, and treatment of these clinical conditions should be a priority in those who work with female athletes. Increased energy availability and restoration of gonadal function are the corner -stones of treatment.

## A1 From theory to practice: Promoting sedentary behavior awareness

### Ana Cikač, Kaja Teraž, and Saša Pišot

#### Science and Research Centre Koper, Institute for Kinesiology Research, Koper, Slovenia

##### **Correspondence:** Ana Cikač (ana.cikac@zrs-kp.si)

*BMC Proceedings 2024*, **18(11)**: A1

**Background**

Although sedentary behavior is still an under-researched area, some studies have shown a significant association between prolonged sitting and an increased risk of mortality, due to various causes, independent of physical activity [1]. As part of the “Knowledge for Health” event, we created a brief online quiz “Sedometer” using a figurative scale to help participants estimate their daily sedentary time thus promoting a better understanding of the associated health risks.

**Materials and methods**

The quiz questions were asked following the WHO definition of »sedentary behavior«, encompassing activities involving low energy expenditure while sitting or lying down in various daily settings like work, school, home, and transportation [2]. Participants subjectively reported the time they spent sitting on a typical day and received their sedentary results at the end [3]. We also examined possible differences between age groups and different “sedentary activities”.

**Results**

The analysis confirmed sedentary behavior across all age groups, although possible differences were identified. Participants reported overall daily sitting time of 533.0 ± 224.7 min, ranging from 20 min to 1160 min. The 15–18-year-old group reported the highest sitting time (590.5 ± 146.0 min), while the oldest group reported the lowest (394.3 ± 106.8 min). Participants reported time spent sitting for specific sedentary activities (Table 1). This total was compared to their overall estimation of sedentary time, indicating an underestimation. Results are not statistically significant, further research on sedentary behavior in various age groups is needed.

**Conclusions**

The WHO recommends reducing sedentary behavior by decreasing sitting time, incorporating interruptions from prolonged sitting as frequently as possible, and increasing moderate to vigorous physical activity [2]. However, there are currently no specific guidelines for individuals to self-assess their sedentary behavior. To address this gap and highlight the health risks of sedentary lifestyle, a quiz was developed as part of an event. Despite limitations in the research method, the positive response to the “bad” results across all age groups indicates the effectiveness of the awareness-raising initiative.

**References**

1. Van der Ploeg HP. Sitting time and all-cause mortality risk in 222,497 Australian adults. Arch Intern Med. 2012;172(6):494.
2. World Health Organization. WHO guidelines on physical activity and sedentary behaviour. S.L.: S.N; 2020.
3. Aunger J, Wagnild J. Objective and subjective measurement of sedentary behavior in human adults: a toolkit. Am J Hum Biol. 2020;34(1).

**Table 1 (abstract A1).** The sum of estimated time spent on sedentary activities, presented in frequencies

Variable	Age group (years)					
	≤15 (*N* = 1)	16-18 (*N* = 20)	19-30 (*N* = 32)	31-45 (*N* = 27)	46-64 (*N* = 20)	65-80 (*N* = 15)
< 240 min			12.5	7.4	15.0	6.7
240-360 min			12.5	14.8	5.0	33.3
361-600 min	100.0	65.0	40.6	40.7	25.0	53.3
601-720 min		15.0	21.9	11.1	25.0	6.7
>720 min		20.0	12.5	25.9	30.0	
TOTAL	100.0	100.0	100.0	100.0	100.0	100.0

## A2 Physical activity in Vojvodina: Is there a gendered pattern?

### Višnja Đorđić, Tatjana Tubić, and Lidija Marković

#### Faculty of Sport and Physical Education, University of Novi Sad, Novi Sad, Serbia

##### **Correspondence:** Lidija Marković (lidija.markovic@fsfvns.edu.rs)

*BMC Proceedings 2024*, **18(11)**: A2

**Background**

Regular physical activity (PA) yields numerous positive outcomes, both in terms of physical health, and psychological well-being [1]. Since PA is a modifiable behavior crucial for the promotion of public health, policies focused on monitoring and enhancing PA should be developed [2,3]. The aim of the study was to analyze gender differences in frequency and level of PA, preferred PA settings and motivators for PA in Vojvodina region.

**Materials and methods**

The sample included 1495 participants (691 men, 804 women) aged 15+ from the Vojvodina region. The study applied the Eurobarometer survey, which includes questions about frequency and level of PA, as well as settings and motivators for engagement in PA [4].

**Results**

The results indicated statistically significant gender differences in exercising/playing sport (χ2(6, *N*=1495) = 48.591, *p* < 0.001), as well as in the frequency and duration of vigorous physical activities (VPA), favoring men (χ2(8, *N*=1495) = 35.796; *p* < 0.001, χ2(6, *N*=1495) = 80.797, *p* < 0.001, respectively), while differences in the frequency of walking were in favor of females (χ2(8, *N*=1495) = 17.112, *p*=0.029). No significant differences were observed in frequency of moderate PA and sedentary behavior. Gender preferences in PA settings were evident, with men favoring sports clubs (χ2(1, *N*=1495) = 31.080; *p* < 0.001), centers (χ2(1, *N*=1495) = 21.629, *p* < 0.001), and exercise at work (χ2(1, *N*=1495) = 15.521; *p* < 0.000), and women preferring home exercising (χ2(1, *N*=1495) = 25.473, *p* < 0.001), and active transport (χ2(1, *N*=1495) = 14.220, *p* < 0.001). Regarding reasons for engaging in PA, although main motivators in both genders were the same (health, relaxation, and better physical appearance), some gender differences were observed. Men more frequently practiced PA for enjoyment (χ2(1, *N*=1495) = 22.256, *p*=0.000), socializing (χ2(1, *N*=1495) = 14.118, *p*=0.000), performance improvement (χ2(1, *N*=1495) = 21.671, *p*=0.000), and competitiveness (χ2(1, *N*=1495) = 26.682, *p*=0.000), while women engaged in PA more often to control body weight (χ2(1, *N*=1495) = 6.191, *p*=0.013).

**Conclusions**

The study reveals gender disparities in PA engagement and preferences. Men exhibit higher involvement in VPA, and a preference for formal PA settings. Conversely, women favor exercising at home and using active transport. The gendered pattern of motivation is identified, as men are more motivated by enjoyment, socializing, performance improvement, and competitiveness, while women are more motivated by weight control. These findings should be considered when developing effective strategies for PA promotion.

**References**

1. Pinckard K, Baskin KK, Stanford KI. Effects of exercise to improve cardiovascular health. Front Cardiovasc Med. 2019;6:69.
2. WHO Regional Office for Europe. Report on the fifth round of data collection, 2018–2020: WHO European Childhood Obesity Surveillance Initiative (COSI). Copenhagen: WHO Regional Office for Europe; 2022.
3. Marković L, Đorđić V, Trajković N, Božić P, Halaši S, Cvejić D, Ostojić SM. Childhood obesity in Serbia on the rise. Children. 2021;8(5):409.
4. European Commission. Special Eurobarometer 525: Sport and physical activity. Brussels: European Commission; 2022.

**Table 1 (abstract A2).** Gender differences in PA frequency and levels, settings, and motivators (*N* = 1495)

		Value	p
Frequency and Level of PA	Exercising/playing sports	48.591	0.000
	Frequency of VPA	35.796	0.000
	Duration of VPA	80.797	0.000
	Frequency of MPA	19.020	0.008
	Duration of MPA	4.527	0.807
	Frequency of walking	17.112	0.029
	Duration of walking	10.911	0.143
	Sedentary behavior	10.575	0.392
PA Settings	Fitness center	2.836	0.092
	Sports club	31.080	0.001
	Sports center	21.629	0.001
	At School/University	1.668	0.197
	At work	15.521	0.000
	At home	25.473	0.001
	Active transport	14.220	0.001
	In the park/Outdoors	2.876	0.090
	Elsewhere	0.007	0.934
	Don’t know	1.477	0.224
	Health	0.325	0.569
	Physical appearance	3.001	0.083
	Counteracting the effect of ageing	0.050	0.824
	Enjoyment	22.256	0.000
	Relaxation	1.990	0.158
	Socializing	14.118	0.000
	New acquaintances	2.288	0.130
	Meeting people from other cultures	0.093	0.761
	Physical performance	21.671	0.000
	Fitness	0.304	0.581
	Weight control	6.191	0.013
	Self-esteem	0.133	0.715
	Learning new skill	0.144	0.705
	Competitiveness	6.191	0.013
	Better integration into society	0.043	0.836
	Other	0.165	0.685
	Don’t know	2.226	0.136

## A3 Effects of additional plyometric training with elastic bands on the jump performance, change of direction, and repeated sprint ability of female handball players

### Damjan Jakšić, Stefan Maričić, Vladimir Šipka, Radenko Matić, and Jovan Vuković

#### Faculty of Sport and Physical Education, University of Novi Sad, Novi Sad, Serbia

##### **Correspondence:** Stefan Maričić (stefan.maricic@fsfvns.edu.rs)

*BMC Proceedings 2024*, **18(11)**: A3

**Background**

Previous research shows that plyometric training is commonly used to improve athletic performance in various sports, especially team sports [1]. Adding extra plyometric exercises seems to be beneficial for developing explosive strength in handball players [2]. The plyometric training with elastic band seems to help produce more force by allowing a greater range of motion and faster muscle contraction. This can lead to better performances in explosive strength which may be important for handball.

**Materials and methods**

The research involved the participation of female handball players exclusively from handball club “Srem Santa” from Sremska Mitrovica. Sixteen participants (age: 20.05 ± 1.02 years) were randomly assigned to two groups: experimental (*N* = 8) and control (*N* = 8). Testing was conducted at the start and end of an 8-week mid-season period.

During this time, the experimental group had extra training with elastic band-resisted jumps two times per week. The central focus of this investigation was the evaluation of explosive strength, which was assessed through various tests including squat jump (SJ), countermovement jump (CMJ), vertical jump (VJ), and single leg jumps. Change of direction capabilities were appraised through the Modified T-test and Repeated Change of Direction (RCOD) test.

**Results**

With a group x time interaction, the experimental group statistically improved in the following variables: SJ (*p*=0.025), CMJ (*p*=0.025), VJ (*p*=0.041), single leg left (*p*=0.006), and single leg right (*p*=0.001). The tested change of direction variables did not provide statistically significant changes in group x time interaction (*p*>0.05) (Table 1). The experimental group demonstrated notable increases in their jump heights across various jump tests. Furthermore, even though the COD variables did not yield statistically significant changes, observable trends suggested potential improvements in agility.

**Conclusions**

It may be concluded that additional plyometric training performed two times per week during 8 weeks enhances abilities related to game performance, especially jump performance in female handball players. Combining elastic bands and plyometric training, provides additional resistance that pushes the muscles throughout the entire range of motion, encouraging greater force production and muscle activation [3]. Also, variable resistance of bands through entire motion, challenges better muscle activation in both (movement and opposing) directions, leading to better neuromuscular coordination, strength, and power output.

**Acknowledgment**

The preparation of this paper was supported by the Provincial Secretariat for Higher Education and Scientific Research, grant number (142-451-3039/2023-01).

**References**

1. Makaruk H, Starzak M, Suchecki B, Czaplicki M, Stojiljković N. The effects of assisted and resisted plyometric training programs on vertical jump performance in adults: a systematic review and meta-analysis. J Sports Sci Med. 2020;19(2):347-357.
2. Gaamouri N, Hammami M, Cherni Y, Rosemann T, Knechtle B, Chelly MS, van den Tillar R. The effects of 10-week plyometric training program on athletic performance in youth female handball players. Front Sports Act Living. 2023;5:1193026.
3. Aloui G, Hermassi S, Hammami M, Cherni Y, Gaamouri N, Shephard RJ, van den Tillar R, Chelly MS. Effects of elastic band based plyometric exercise on explosive muscular performance and change of direction abilities of male team handball players. Front Physiol. 2020;11:604983.

**Table 1 (abstract A3).** Vertical jump test and change of direction performances in experimental and control groups before and after 8-week intervention

Variable	Experimental group (*N* = 8)		Control group (*N* = 8)			
	Pre	Post	Pre	Post	F	p
Squat jump (cm)	24.27±4.88	27.28±5.10	22.46±2.61	23.85±2.85	2.50	0.025
Countermovement jump (cm)	26.20±5.22	29.04±5.46	24±3.50	24.56±2.52	6.31	0.025
Vertical jump (cm)	30.62±7.13	32.40±6.89	28.60±3.45	28.90±3.38	4.36	0.046
Single left (cm)	14.01±4.05	16.52±4.80	12.66±2.86	11.65±2.09	10.38	0.006
Single right (cm)	12.55±3.48	14.71±3.28	12.99±1.91	12.69±2.57	12.22	0.004
COD (Modified T-test) (s)	8.90±0.74	8.19±0.62	9.21±0.71	8.75±0.64	1.31	0.271
Repeated COD mean (s)	6.59±0.54	6.04±0.67	6.91±0.51	6.42±0.21	0.06	0.808

## A4 Y-balance test performance among previously lower-limb injured and uninjured recreational athletes: A cross-sectional study

### Draženka Mačak, Jelena Slankamenac, Dragan Marinković, Brigita Banjac, Branislav Kokeza, and Danilo Radanović

#### Faculty of Sport and Physical Education, University of Novi Sad, Novi Sad, Serbia

##### **Correspondence:** Draženka Mačak (drazenka.macak@fsfvns.edu.rs)

*BMC Proceedings 2024*, **18(11)**: A4

**Background**

Previous injury is an ominous risk factor for recurrent lower limb injury in athletic populations [1]; thus, reliable protocols must be established for detecting a person’s injury risk. The Y balance test (YBT) measures dynamic balance, and even though the YBT ability to predict lower limb injury risk in athletes has been a subject of previous research, it failed to provide consistent data [2, 3]. Therefore, this study evaluated the YBT performance among previously uninjured and injured athletes.

**Materials and methods**

Athletes were categorized into previously injured (*N* = 18; age = 20.2±0.71; BMI = 23.63±2.70) and uninjured (*N* = 44; age = 20.24±0.54; BMI = 24.40±2.29). The study included recreational athletes with a minimum of 3 years of experience in both groups and a history of any musculoskeletal injuries (for the previously injured group) or without any (for the uninjured group). The anterior, posteromedial, and posterolateral YBT reach distances, limb lengths, and dominant leg were measured, along with bilateral deficit respecting each direction distance.

**Results**

General linear models estimated the differences between the groups’ YBT performance while controlled for leg length and showed that the mean performance of Y balance test items of the previously injured athletes’ group was significantly lower as compared to the group of uninjured athletes (F (9, 50) = 2.89, *p*<0.01, partial η2=0.34). The group effects on the YBT items' performance ranged from medium (posteromedial reach, η2=0.09) to large (anterior-posterior reach, η2=0.23). No significant differences were, however, observed between the groups’ bilateral deficits as measured by the YBT. Finally, YBT performance could not detect a person with previous injury (AUC=0.24 [0.11, 0.38]; Table 1).

**Conclusions**

Even though the findings suggest differences between the YBT performance of athletes with and without prior lower limb injuries, the difference in bilateral deficits is lacking and logistic model failed to fit the data to estimate the probability of an individual having a previous injury based on YBT performance. Hence, further research must provide data, establish criteria, and detect the model emphasizing lower limb injury risk.

**References**

1. Toohey L, Drew M, Cook J, Finch C, Gaida J. Is subsequent lower limb injury associated with previous injury? A systematic review and meta-analysis. Br J Sports Med. 2017;51:bjsports-2017.
2. Alkhathami K. Using the Y-balance test as a predictor tool for evaluating non-contact injuries in university league football players: A prospective longitudinal study. Cureus. 2023;15.
3. O'Connor S, McCaffrey N, Whyte EF, Fop M, Murphy B, Moran K. Can the Y balance test identify those at risk of contact or non-contact lower extremity injury in adolescent and collegiate Gaelic games? J Sci Med Sport. 2020;23:943–948.

**Table 1 (abstract A4).** Fitting logistic models of the previous injury in the function of YBT performance

YBT predictors (cm)					95% CI	
	β	Wald F_(1)_	p	Odds ratio	Lower	Upper
Testing model (Model 1)						
anteriorrightMAXa	0.281	4.569	0.033	1.325	1.024	1.715
anteriorleftMAXa	-0.337	7.465	0.006	0.714	0.561	0.909
pmedialrightMAX	-0.097	2.135	0.144	1.102	0.967	1.256
pmedialleftMAX	0.109	2.770	0.096	0.897	0.789	1.019
plateralrightMAX	-0.073	0.511	0.475	1.075	0.881	1.313
plateralleftMAX	-0.053	0.423	0.516	1.054	0.899	1.236
Constant	11.783	6.129	0.013	0.000		
Model 1 summery						
Nagelkerke R^2^	0.409					
Overall % correct	79 yes = 44.4	no = 93.2				
Final model (Model 2)						
anteriorrightMAXa	0.124	1.900	0.168	0.884	0.741	1.054
anteriorleftMAXa	-0.238	7.294	0.007	0.788	0.663	0.937
Constant	6.505					
Model 2 summery						
Nagelkerke R^2^	0.262					
Overall % correct	77.4 yes = 33.3	no = 95.5				

a. Controlled for Leg length (cm) in Block 1

β, logistic regression coefficient; Wald F (df) test; p, *p* value; 95% CI, confidence interval

## A5 Impact of elasticity on explosive power in athletes of pioneer age from Sjenica

### Ilona Mihajlović, Vuk Ralić, Nikola Radulović

#### Faculty of Sport and Physical Education, University of Novi Sad, Novi Sad, Serbia

##### **Correspondence:** Nikola Radulović (nikola.radulovic@fsfvns.edu.rs)

*BMC Proceedings 2024*, **18(11)**: A5

**Background**

Athletics is deemed a basic sport because it is incorporated in most sports. As such, it is in the focus of interest of everybody in terms of the specific equation for its successful performance.

**Materials and methods**

UThe aim of the research was to establish the ratio of individual parameters for assessment of elasticity onto manifestation of explosive power in 34 athletes, boys (*n*=20), and girls (*n*=14), from Sjenica (12.53 ± 0.52 and 12.35 ± 0.39 years, respectively). The predictor variables were as follows: stick twist, deep forward bend on the bench, split and leaning forward, whereas the following was selected as criteria: standing long jump, the Abalak test, and a 2 kg medicine ball throwing. Mathematical statistical data processing included the calculation of descriptive properties. For the purpose of defining the impact of the predictor system to the selected criteria, linear regression analysis was applied.

**Results**

Using linear regression analysis, it was established that there is statistically significant impact of the system of predictor variables *p*=0.00. The percentage of common variability was somewhat higher in young female athletes and ranged within the scope from 42% (for the criterion 2 kg medicine ball throwing) to 55% (for the criterion long jump without a running start). In young male athletes, the common variability ranged within the scope of 30% (for the criterion long jump without a running start) to 39% (for the criterion Abalak test) [1]. The highest predictive impact on manifestation of explosive power of leg muscles was achieved for the variable deep forward bend on the bench.

**Conclusions**

The authors conclude that standard motoric tests for assessment of elasticity of the upper leg hamstrings, the arms, and the shoulder girdle largely determine the manifestation of explosive power of arms and legs in young athletes of both genders [2].

**References**

1. Donti O, Tsolakis C, Bogdanis GC. Effects of baseline levels of flexibility and vertical jump ability on performance following different volumes of static stretching and potentiating exercises in elite gymnasts. J Sport Sci Med. 2014 Jan 20;13(1):105-113.
2. Place N, Blum Y, Armand S, Maffiuletti NA, Behm DG. Effects of a short proprioceptive neuromuscular facilitation stretching bout on quadriceps neuromuscular function, flexibility, and vertical jump performance. J Strength Cond Res. 2013 Feb;27(2):463-470.

**Table 1 (abstract A5).** Results of the regression analysis for all three tests

Variable	Standing long jump				Abalak test				2kg medicine ball thrrowing			
	r	p	β	pβ	r	p	β	pβ	r	p	β	pβ
♂												
Stick twist	0.04	0.39	0.10	0.46	-0.03	0.41	0.06	0.71	-0.08	0.29	0.04	0.77
Deep forward bend on the bench	0.31	0.02	0.21	0.59	0.11	0.25	-0.05	0.91	0.26	0.04	-0.01	0.97
Split	0.44	0.01	0.41	0.00	0.60	0.01	0.61	0.01	0.53	0.01	0.51	0.01
Leaning forward	0.30	0.02	0.08	0.80	0.10	0.25	0.07	0.81	0.27	0.03	0.23	0.51
♀												
Stick twist	-0.32	0.05	-0.10	0.48	-0.24	0.11	-0.08	0.58	0.26	0.09	0.15	0.68
Deep forward bend on the bench	0.65	0.01	0.83	0.02	0.70	0.01	0.47	0.18	-0.59	0.01	-0.52	0.04
Split	0.45	0.01	0.25	0.09	0.14	0.24	-0.04	0.72	0.30	0.06	-0.04	0.91
Leaning forward	0.51	0.01	-0.30	0.36	0.67	0.00	0.23	0.48	0.39	0.02	0.27	0.12
R	♂	0.52	♀	0.73	♂	0.60	♀	0.71	♂	0.57	♀	0.66
R^2^		0.30		0.55		0.39		0.51		0.33		0.42
P		0.00		0.00		0.00		0.00		0.00		0.00

## A6 Correlation between anthropometric measures and motor test performance among students

### Boris Popović, Nina Nikolić, Milan Cvetković, Nikola Manolopoulos

#### Faculty of Sport and Physical Education, University of Novi Sad, Novi Sad, Serbia

##### **Correspondence:** Milan Cvetković (milan.cvetkovic@fsfvns.edu.rs)

*BMC Proceedings 2024*, **18(11)**: A6

**Background**

College is a transitional period from adolescence to adulthood, and is also a crucial period for the development of healthy lifestyles and the formation of healthy behaviors [1]. With increasing awareness about the health benefits of physical activity, the role of sport in society has become increasingly important for the promotion of public health [2]. Anthropometric measures, such as body weight, body height, including the body mass index (BMI) are associated with motor test performance and have been proposed as predictors of physical fitness among adolescents and students [3,4,5]. The present study aimed to determine the correlation between anthropometric characteristics and motor test performance in university students.

**Materials and methods**

Ninety-one participants (28 male and 63 female students aged 21.5±0.89) from the University of Novi Sad were included in this cross-sectional study. Motor test performance was assessed with standing long jump, hand grip strength, push-ups, 60s sit-ups and sit and reach. Anthropometric measures were evaluated with body height, body weight, waist and hip circumference.

**Results**

Multiple linear regression showed that standing long jump highly correlated with body height (t=3.79; *p*=0.00); waist circumference (t=2.98; *p*=0.00) and hip circumference (*t*=-2.92; *p*=0.01); hand grip strength correlated with body height (*t*=2.10; *p*=0.04) and hip circumference (*t*=-2.39; *p*=0.02); push-ups with body height (*t*=2.40; *p*=0.02); 60s sit-ups with body height (*t*=2.02; *p*=0.05) and hip circumference (*t*=-1.98; *p*=0.05); sit and reach test with waist circumference (*t*=-2.16; *p*=0.03).

**Conclusions**

The findings underscore the importance of anthropometric measures in understanding motor performance. Furthermore, the study's insights contribute to the ongoing discourse on the optimization of physical performance and rehabilitation outcomes. Professionals in fields ranging from sports training to physical therapy can leverage this knowledge to refine their methodologies, ensuring that interventions are not only evidence-based but also attuned to the unique attributes of students.

**References**

1. Niedermeier M, Frühauf A, Kopp-Wilfling P, et al. Alcohol consumption and physical activity in Austrian college students-a cross-sectional study. Subst Use Misuse. 2018;53(10):1581–1590.
2. Tahira, S. The association between sports participation and physical fitness. Int. J. Sport Stud. Health. 2021;4:e127001.
3. Lopes, V. P., Cossio-Bolaños, M., Gómez-Campos, R., de Arruda, M., Hespanhol, J. E., and Rodrigues, L. P. Linear and nonlinear relationships between body mass index and physical fitness in Brazilian children and adolescents. Am. J. Hum. Biol. 2017;29(6):e23035.
4. García, R. L., Carrasco, J. O. L., and Castruita, R. M. C. Body composition, body indexes and somatotype in university handball players. VISUAL review. Int. Visual Cult. Rev. 2022;9:1–11.
5. Ben Brahim, M., Sal-de-Rellán, A., Hernaiz-Sánchez, A., Yasin, H. and García-Valverde, A. The relationships between body mass index, reciprocal ponderal index, waist-to-height ratio, and fitness in young adult males. Front. Psychol. 2023;14:1250913.

**Table 1 (abstract A6).** Multiple linear regression of anthropometric measures and motor test performance

Variables	Standing long jump		Hand grip strength		Push ups		60s Sit ups		Sit and reach	
	t	p	t	p	t	p	t	p	t	p
Body height	3.79	0.00	2.10	0.04	2.40	0.02	2.02	0.05	0.70	0.49
Body weight	-0.45	0.65	1.42	0.16	-0.21	0.83	0.54	0.59	-0.12	0.90
Waist circumference	2.98	0.00	1.18	0.24	0.95	0.35	1.01	0.32	-2.16	0.03
Hip circumference	-2.92	0.01	-2.39	0.02	-1.31	0.19	-1.98	0.05	0.15	0.88

*t t*-value for multiple linear regression p – statistical significance ≤ 0.05

## A7 Time and mechanical parameters in three different swimming starts

### Milorad Jakšić, Goran Dimitrić, and Maja Batez

#### Faculty of Sport and Physical Education, University of Novi Sad, Novi Sad, Serbia

##### **Correspondence:** Milorad Jakšić (milorad.jaksic@fsfvns.edu.rs)

*BMC Proceedings 2024*, **18(11)**: A7

**Background**

With growing interest in competitive swimming, analyzing race performances has become increasingly important [1]. Different phases of swimming, including the start, are crucial for overall performance [2]. Traditionally, two start techniques, grab and track, were commonly used. However, the introduction of the new starting block OMEGA OSB11 allowed for a third technique, the kick start, to be employed. Research has suggested that the kick start offers advantages over grab and track starts, including shorter block time, flight time, and faster speeds [3]. This study aimed to assess the performance differences among these three techniques in novice swimmers.

**Materials and methods**

Twenty male students with no prior swimming experience participated in the study. They performed three attempts of each start technique, and kinematic data were collected using video recordings. Variables such as time on the block, flight time, total time, flight distance, flight velocity, take-off angle, and angle of entry were analyzed. Statistical analysis was conducted using SPSS.

**Results**

Based on ANOVA and statistical significance (Table 1), significant differences were noted in three of seven analyzed parameters reflecting start efficiency: Block Time (*p*=0.00), Total Time (*p*=0.00), and Flight Speed (*p*=0.00). No significant differences (*p*>0.05) were observed for the remaining four variables. The "Kick start" technique emerged as the most efficient, with notably shorter Block Time, Total Flight Time, and higher Flight Speed compared to traditional "Grab start" and "Track start" techniques.

**Conclusions**

The study concludes that, among participant not actively engaged in swimming, the Kick start proves to be the most efficient technique. It significantly reduces block time, total flight time, and contributes to greater flight speed compared to Grab and Track starts. These findings underscore the advantages of employing the Kick start in the context of amateur swimming.

**References**

1. Cossor J, Slawson S, Shillabeer B, Conway P, West A. Are land tests a good predictor of swim start performance? Port J Sports Sci. 2011;11(2):183–186.
2. Thow JL, Naemi R, Sanders RH. Comparison of modes of feedback on glide performance in swimming. J Sports Sci. 2012;30(1):43–52.
3. Honda K, Sinclair P, Mason B, Pease D. The effect of starting position on elite swim start performance using an angled kick plate. In: Proceedings of the 30th Annual Conference of Biomechanics in Sports; 2012; Melbourne. p. 72–75.

**Table 1 (abstract A7).** Differences between groups

Variable	Grab start (Mean)	Track start (Mean)	Kick start (Mean)	F	p
Time on block (s)	0.91	0.80	0.70	15.81	0.00
Flight time (s)	0.28	0.27	0.28	0.40	0.67
Total time (s)	1.19	1.03	0.99	12.48	0.00
Flight distance (cm)	99.89	90.90	102,31	1.38	0.26
Flight speed (cm/s)	85.27	82.68	104.61	6.40	0.00
Take-off angle (°)	27.53	30.03	32.03	2.59	0.08
Angle of entry (°)	36.43	37.28	37.15	0.11	0.89

## A8 The global sporting arms race: A comparative analysis of sport system models

### Berkay Ayverdi and Hakan Sunay

#### Faculty of Sport Sciences, Department of Sports Management, Ankara University, Ankara, Türkiye

##### **Correspondence:** Berkay Ayverdi (berkay.ayverdi@ankara.edu.tr)

*BMC Proceedings 2024*, **18(11)**: A8

**Background**

The importance given to sports systems by countries dates back to the 1950s and the beginning of the Cold War. In these years, with the beginning of the Cold War, governments developed policies and produced systems for the development of elite athletes. Especially in the period between 1950 and 1980, it is seen that the Soviet Union and East Germany attached a high level of importance to elite sports development. As a result of these events, it has been observed that there has been a significant increase in interest in comparative sports management. This competition is defined as “The Global Sporting Arms Race” [1].

**Materials and methods**

This study aims to provide an overview of comparative sports management by making a comprehensive analysis of the sports systems available in the academic literature. Document analysis, one of the qualitative research methods, was preferred for this research. With this method, existing sports system models were examined, and a detailed comparison was made. During the research process, a comprehensive academic literature review was carried out to determine the critical findings and different models in the literature. During data analysis, content and descriptive methods were preferred, and MAXQDA software was used.

**Results**

As a result, five crucial sports system models (policy-related) have been reached [2; 3; 4; 5; 6]. A total of 37 elements were reached, later reduced to 12 by the researchers. 12 themes and 25 codes emerged (Table 1). The elements that should be in a sports system are defined as follows: Organization and Sport Policy, Sport Participation, Financial Support, Training Facilities and Equipment, Career Support, Sport Sciences, (Inter) National Competition, Coach Development, Sport Environment, Anti-Doping Targeting resources on few sports, Armed Forces.

**Conclusions**

The factors recommended for a sports system are similar, but some differences have been determined. There is a consensus on the elements of a successful sports system.

As a result, it can be said that Organization and Sport Policy, Sport Participation and Financial Support are the most important factors for the success of international support, namely, “The Global Sporting Arms Race”. Anti-Doping, Targeting Resources on a few Sports and Armed Forces factors are less important.

The recommendations for this research are as follows: Longer-term research is expected to uncover more models and compare countries' sports systems.

**References**

1. Oakley B, Green M. The production of Olympic champions: International perspectives on elite sport development systems. Eur J Sports Manag. 2001;(8):83-105.
2. Green M, Oakley B. Elite sport development systems and playing to win: uniformity and diversity in international approaches. Leis Stud. 2001;20(4):247-267.
3. Digel H. A comparison of competitive sport systems. New Stud Athl. 2002.
4. De Bosscher V, De Knop P, Van Bottenburg M, Shibli S. A conceptual framework for analysing sports policy factors leading to international sporting success. Eur Sport Manag Q. 2006;6(2):185-215.
5. Böhlke N, Robinson L. Benchmarking of elite sport systems. Manag Decis. 2009;47(1):67-84.
6. Hallmann K, Petry K. Comparative sport development. Springer Nature. 2013.

**Table 1 (abstract A8).** Themes, codes and frequency

Theme (12)	Code (25)	Frequency
Organization and Sport Policy	• Organizational Culture • Constitutional Law • Mass Sport Policy • Elite Sport Policy • Comprehensive Planning • Clear roles in the organization • Communication	8
Sport Participation	• Clubs • Talent Development	6
Financial Support	• Government Budget • Other Incomes	4
Training Facilities and Equipment	• Location • Accessibility • Qualification	3
Career Support	• After Career • During Career	3
Sport Sciences	• Research Institutes • Universities	3
(Inter) National Competition	• International Exposure • National Exposure	3
Coach Development	• Salary • Insurance	2
Sport Environment	• Media • Education System • Culture	2
Anti-Doping		1
Targeting resources on few sports		1
Armed Forces		1

## A9 Age at peak performance of elite male and female volleyball players

### Sunčica Poček, Duško Cvijović, and Lana Bajić

#### Faculty of Sport and Physical Education, University of Novi Sad, Novi Sad, Serbia

##### **Correspondence:** Sunčica Poček (suncica.pocek@fsfvns.edu.rs)

*BMC Proceedings 2024*, **18(11)**: A9

**Background**

The long-term goal of an athletic career should be sport mastery defined as consistent, successful, senior international competitive performance. Over many years of systematic training an athlete will develop abilities and skills, which with planned peaking, should result in a high level of performance [1]. The aim of this study was to examine chronological age at peak performance of volleyball players who competed at world championships 2022.

**Materials and methods**

The sample of subjects consisted of 672 volleyball players of which 336 men and 336 women (1.96±0.08 m, 87.7±9.5 kg; and 1.83±0.08 m, 70.6±8.5 kg, of body height and body mass, respectively). A one-way ANOVA was applied for analysis of player’s position (setter, opposite, middle blocker, outside hitter, and libero), and performance level (level 1: 1st – 4th, level 2: 5th – 8th, level 3: 9th to last), while for the gender differences was applied t-test. Statistical significance was set at p≤0.05.

**Results**

Age at peak performance of elite volleyball players is 27.6±4.4 and 26.0±4.1 years for men and women, respectively (*t*=4.87, *p*=0.00). Regarding player’s position (Table 1), there are differences for men (F=3.60, *P*=0.01), but not for women (F=0.91, *P*=0.46). Male setters are older than outside hitters, while libero players are older than both middle blockers and outside hitters. In terms of performance level there are differences for women (F=3.45, *P*=0.03). The best female volleyball players are older than lower performance level players. Although for men we observed differences, values didn’t reach statistically significant threshold (F= 2.58, *P*=0.08).

**Conclusions**

Knowledge of age at peak performance in volleyball could inform decisions about selection and preparation of athletes for specific events [1,2,3]. The results of this study may be used as information about the time required to reach the highest level of competition in volleyball, to estimate the chronological setting of the phase for in-depth specialization towards maximum performance [4,5].

**References**

1. Palao JM, Manzanares P, Valadés D. Anthropometric, physical, and age differences by the player position and the performance level in volleyball. J Hum Kinet. 2014 Dec 1;44(1):223-236.
2. Hollings SC, Hopkins WG, Hume PA. Age at peak performance of successful track & field athletes. Int J Sports Sci Coach. 2014 Sep;9(4):651-661.
3. Longo AF, Siffredi CR, Cardey ML, Aquilino GD, Lentini NA. Age of peak performance in Olympic sports: A comparative research among disciplines. J Hum Sport Exerc. 2016;11(1):31-41.
4. Lloyd RS, Oliver JL, Faigenbaum AD, Myer GD, De la Croix MB. Chronological age vs. biological maturation: implications for exercise programming in youth. J Strength Cond Res. 2014 May 1;28(5):1454-1464.
5. Smith DJ. A framework for understanding the training process leading to elite performance. Sports Med. 2003 Dec;33:1103-1126.

**Table 1 (abstract A9).** Age at peak performance of elite male and female volleyball players

	Age	
	Male	Female
Setter	28.8±4.2^oh^	26.8±3.8
Opposite	27.4±4.8	26.0±4.3
Middle blocker	27.5±4.0	25.8±4.2
Outside hitter	26.6±4.5^s,l^	25.6±4.0
Libero	28.9±3.9^oh^	26.3±4.3
∑	27.6±4.4	26.0±4.1
	F=3.60 *P*=0.01	F=0.91 *P*=0.46

## A10 Is there a difference in the functional stability of women according to their physical activity?

### Jelena Obradović, Mila Vukadinović Jurišić, Anja Obradović, Marko Manojlović, and Patrik Drid

#### Faculty of Sport and Physical Education, University of Novi Sad, Novi Sad, Serbia

##### **Correspondence:** Mila Vukadinović Jurišić (mila.vukadinovic@fsfvns.edu.rs)

*BMC Proceedings 2024*, **18(11)**: A10

**Background**

Functional movement capability is the effective movement in various fundamental movement patterns and motor skills, specifically characterized by the human body's mobility, stability, coordination, and symmetry [1]. The aging process and sedentary lifestyle decrease functional movement capability [2] and influence functional stability and mobility in women. The aim of this study was to find out the difference in functional stability and mobility of middle-aged women who are involved in the Dynamic Neuromuscular Stabilization and Hatha Yoga program.

**Materials and methods**

Forty-one middle- aged women (age 51.37±6.16 years) divided into groups based on physical activity: dynamic neuromuscular stabilization group (DNS, *N*=13, body height 169.0±5.67 cm, body mass 70.15±6.79 kg), yoga group (YOGA, *N*=13, body height 167.92±5.36 cm, body mass 69.92±9.12 kg) and control group (CON, *N*=15, body height 168.80±3.87 cm, body mass 70.87±8.63 kg). The participants were measured by the Functional Movement Screening (FMS) system [3]. The Kruskal-Wallis Test and univariate analysis of variance was used for statistical analysis.

**Results**

Table 1 shows significant differences (p≤0.01) in the Deep Squat, Hurdle Step, In-Line Lunge, Rotary Stability, Trunk Stability Push-Up and Shoulder Mobility between groups. In Active Strength Leg Raise, there are no significant differences (p≥0.01) between groups (Table 1). There were significant differences in FMS Total Score between groups (p≤0.01), with the DNS group (17.62±1.50) outperforming the YOGA group (12.69±2.84) and CON group (13.27±3.06).

**Conclusions**

The DNS group had good functional mobility of the hip, knees, ankles joints and excellent functional stability of the trunk. The YOGA group had a good functional mobility of the shoulders. The CON group had insufficient functional stability of the trunk and mobility of the shoulders, hip, knee, and ankle. The women who exercised Dynamic Neuromuscular Stabilization for one year had better functional stability and mobility than women who exercised Hatha Yoga or did not exercise. Middle-aged women should increase physical activity to improve the body functional capability.

**References**

1. Huang J, Zhong M, Wang J. Effects of exercise-based interventions on functional movement capability in untrained populations: a systematic review and meta-analysis. Int J Environ Res Public Health. 2022; 19(15):9353.
2. Tomás MT, Galán-Mercant A, Carnero EA, Fernandes B. Functional capacity and levels of physical activity in aging: a 3-year follow-up. Frontiers in medicine. 2018;4:244.
3. Cook G, Burton L, Hoogenboom BJ, Voight M. Functional movement screening: the use of fundamental movements as an assessment of function-part 2. Int J Sports Phys Therapy. 2014; 9(4):549-563.

**Table 1 (abstract A10).** Kruskal-Wallis Test results

Variable	DNS GROUP (*N* = 13)	YOGA GROUP (*N* = 13)	CON GROUP (*N* = 13)	*p*
Deep squat	24.58	14.00	23.97	0.01
Hurdle step	27.54	23.42	13.23	0.00
In-line lunge	35.62	17.42	24.73	0.00
Shoulder mobility	29.73	37.88	16.17	0.00
Active strength leg raise	29.19	24.88	27.90	0.42
Trunk stability push-up	42.77	13.46	22.07	0.00
Rotary stability	44.19	16.88	23.70	0.00

## A11 “10 minutes for health” - Examining the effectiveness of workplace health promotion programs

### Bence Cselik^1^, Ágnes Borsos^2^, János Girán^3^, and Nikolett Ildikó Tumpek^1^

#### ^1^Institute of Physiotherapy and Sports Science, Faculty of Helth Science, University of Pécs, Pécs, Hungary; ^2^Faculty of Engineering and Information Technology, University of Pécs, Pécs, Hungary; ^3^Medical School, University of Pécs, Pécs, Hungary

##### **Correspondence:** Bence Cselik (cselik.bence@pte.hu)

*BMC Proceedings 2024*, **18(11)**: A11

**Background**

Office design optimization is crucial for enhancing productivity and employee well-being. The shift from cubicles to open layouts aimed at addressing social and health needs but introduced challenges like increased noise and compromised privacy [1, 2]. Currently, research is focused on preventing health problems, therefore health improvement is not addressed. An important pillar of this is to achieve a high percentage of people in active employment. Europe is characterized by an ageing society, so the most important factor is to keep workers active for longer, and this is potentially the key to increasing employment intensity. This condition is influenced by a number of factors, primarily the physical environment of the workplace the physical factors of the workplace and the psychosocial factors of the individual.

**Materials and methods**

The goal of the present study was to assess the effectiveness of a prevention program aimed at reducing health risks associated with office workplaces, particularly those related to sedentary work. In the framework of the prevention program, a group of employees of the Rector's Office of the University of Pécs were selected to participate in a specific architectural and health promotion program, which was designed to reduce the risk of health problems caused by office work.

**Results**

Overall, the sedentary office worker training program was popular amongst workers. They found the application of the Workpad useful, yet as more and more people are working with laptops instead of personal computers, the ergonomics of their work is only partially implemented, which needs to be addressed in the future. Amongst the physical activity program, walking was the most popular, probably due to the fact that in most of the participating environments this form of activity is the easiest to be implemented and achieved.

**Conclusions**

Our research highlighted that employees are not sufficiently active due to their sedentary work habits. Consequently, during the planning phase, we capitalized on the opportunity to integrate this essential aspect into the office design [3]. The holistic approach of the program, encompassing both physical and mental well-being, is reflected in the positive change observed in the WHO well-being index. Moreover, the program's success underscores the significance of incorporating health promotion initiatives into the workplace.

**References**

Cakó B, Zoltán ES, Girán J, Medvegy G, Miklós ME, Nyers Á, Grozdics AT, Kisander Z, Bagdán V, Borsos Á. An efficient method to compute thermal parameters of the comfort map using a decreased number of measurements. Energies. 2021;14:5632. https://doi.org/10.3390/en14185632

Jimenez P, Bregenzer A. Integration of eHealth tools in the process of workplace health promotion: Proposal for design and implementation. J Med Internet Res. 2018. doi:10.2196/jmir.8769

Yassaee M, Mettler T, Winter R. Principles for the design of digital occupational health systems. Elsevier Inf Organ. 2019 Jun;29(2):77-90. 10.1016/j.infoandorg.2019.04.005

**Table 1 (abstract A11).** The change between the results of the willingness to do sports between the two measurement periods

Willingness to do sport	1. measure	2. measure
None	17.87 %	14.28 %
Rarely	25.00 %	17.87 %
1-2x/ month	7.14 %	3.57 %
1-2x/ week	28.54 %	32.14 %
3-4x/ week	17.87 %	21.42 %
Daily	7.14 %	10.41 %

## A12 Acute effects of social dances on self-perceived health: A pre-post pilot study

### Snežana Damjanović, Aleka Vujatović, Draženka Mačak, Boris Popović

#### Faculty of Sport and Physical Education, University of Novi Sad, Novi Sad, Serbia

##### **Correspondence:** Boris Popović (boris.popovic@fsfvns.edu.rs)

*BMC Proceedings 2024*, **18(11)**: A12

**Background**

Self-perceived health is a valid and robust predictor of morbidity and mortality of several diseases and perceived health issues such as cancer, stress, and cardiovascular disease, among other chronic long-term health conditions [1]. Previous research [2, 3, 4] claims that dance positively impacts multidimensional characteristics, including physical, psychological, cognitive, and social domains; therefore, the study objective is to determine the impact of social dances on the perceived health of the middle-aged population.

**Materials and methods**

Inclusion criteria: both males and females, recreational athletes (a minimum of 3 years of experience), attending the course of social dances for a minimum of 6 months, 2/week, healthy, without any musculoskeletal injuries. Exclusion criteria: any neurological, musculoskeletal, and/or endocrine disorder, any present musculoskeletal injury, and a history of musculoskeletal injuries. The sample consisted of 30 recreational (course of social dances for a minimum of 6 months, 2/week) participants (11 males and 19 females) aged from 40 to 60 years. The participants completed the questionnaire about self-perceived health immediately before and after the class, which lasted 60 minutes.

**Results**

The percentage data showed that 83% of participants perceived no health issues before the social dance class, while 93% perceived no health issues after the dance class (Table 1). Therefore, the number of respondents who did not perceive health issues after a dance class has increased by 10%.

**Conclusions**

This pilot study indicates a potential positive impact of the social dance class on how middle-aged persons perceive their health. Dance is an activity that can be equally useful and fun for middle-aged people. Thus, future research is warranted to clarify the social dance outlooks in managing self-perceived health.

**References**

1. Gumà J. What influences individual perception of health? Using machine learning to disentangle self-perceived health. SSM Popul Health. 2021 Dec 9;16:100996.
2. Lu J, Abd Rahman NA, Wyon M, Shaharudin S. The effects of dance interventions on physical function and quality of life among middle-aged and older adults: A systematic review. PLoS One. 2024 Apr 19;19(4):e0301236.
3. Salihu D, Kwan RYC, Wong EML. The effect of dancing interventions on depression symptoms, anxiety, and stress in adults without musculoskeletal disorders: An integrative review and meta-analysis. Complement Ther Clin Pract. 2021 Nov;45:101467.
4. Laird KT, Vergeer I, Hennelly SE, Siddarth P. Conscious dance: Perceived benefits and psychological well-being of participants. Complement Ther Clin Pract. 2021;44:101440

**Table 1 (abstract A12).** Z-test results for pre- and post-measurements

Statistics	PRE	POST
%	83%	93%
z	-1.21	
p	0.23	

## A13 Preliminary results on the nutritional assessment and Mediterranean diet of older adults in Slovenia

### Kaja Teraž^1,2^, Saša Pišot^2^, Ana Cikač^2^, Katarina Puš^2,3^, Boštjan Šimunič^2^

#### ^1^Clinical University Department of Medical, Surgical and Health Sciences, University of Trieste, Trieste, Italy; ^2^Science and Research Center Koper, Institute for Kinesiology Research, Koper, Slovenia; ^3^Faculty of Sport, University of Ljubljana, Ljubljana, Slovenia

##### **Correspondence:** Kaja Teraž (kaja.teraz@zrs-kp.si)

*BMC Proceedings 2024*, **18(11)**: A13

**Background**

Adequate nutrition is increasingly recognized as playing a key role in reducing the risk of non-communicable diseases and promoting health. In addition, to a Mediterranean diet is associated with better health in older adults. It reduces risk of cardiovascular disease, type 2 diabetes mellitus, cancer and it is beneficial with cognitive decline [1, 2]. After conducting an epidemiological study, we were interested in the nutritional status and adherence to the Mediterranean diet (and lifestyle) among older adults in Slovenia and its association with selected body composition parameters.

**Materials and methods**

Measurements were conducted in all Slovenian regions. Older people came to a health center for the measurements, where sociodemographic, nutritional and body characteristics (body height, body mass, body mass index (BMI)) were measured. The nutrition questionnaires were completed as part of a guided interview and evaluated using the Mini Nutritional Assessment (MNA) questionnaire and the MEDLIFE questionnaire. The correlation between body characteristics and dietary habits was assessed using Pearson's coefficient. Differences in adherence to a Mediterranean lifestyle in the different Slovenian regions were assessed using ANCOVA, with gender and age as covariates.

**Results**

A total of 518 older adults (30.1% male) with a mean age of 69.6 ± 6.2 years, a mean body height of 164.7 ± 8.1 cm and a mean body mass 73.4 ± 13.7 kg were included in this analysis. The mean MNA score was 25.7 ± 3.52, while the mean MEDLIFE index was 16.4 ± 3.6. We found differences in the MEDLIFE index between regions, adjusting for gender and age. We also found a weak negative correlation between the MEDLIFE index and body height, body mass and body mass index (*r*=-0.087, *p*=0.048; *r*=-0.130, *p*=0.003; *r*=-0.100, *p*=0.023, respectively).

**Conclusions**

Regarding MNA results, our participants were well-nourished. Nevertheless, we did not find any correlation between MNA score and body characteristics [3]. Our preliminary results suggest that adherence to the Mediterranean lifestyle differs among Slovenian regions. Moreover, we also found that older adults with higher body mass and BMI had lower adherence to the Mediterranean lifestyle.

**References**

1. Barrea L, Muscogiuri G, Di Somma C, Tramontano G, De Luca V, Illario M, Colao A, Savastano S. Association between Mediterranean diet and hand grip strength in older adult women. Clin Nutr. 2019;38(2):721–729. https://doi.org/10.1016/j.clnu.2018.03.012
2. Cacciatore S, Calvani R, Marzetti E, Picca A, Coelho-Júnior HJ, Martone AM, Massaro C, Tosato M, Landi F. Low adherence to Mediterranean diet is associated with probable sarcopenia in community-dwelling older adults: Results from the Longevity Check-Up (Lookup) 7+ Project. Nutrients. 2023;15(4):1026. https://doi.org/10.3390/nu15041026
3. Kastorini CM, Milionis HJ, Esposito K, Giugliano D, Goudevenos JA, Panagiotakos DB. The effect of Mediterranean diet on metabolic syndrome and its components. J Am Coll Cardiol. 2011;57(11):1299–1313. 10.1016/j.jacc.2010.09.073

**Table 1 (abstract A13).** Correlation between selected body characteristics and nutritional assessment. Pearson’s R (*p*-value) is reported

Variable	MNA	MEDLIFE index
Body height	0.005 (0.910)	-0.087 (0.048)
Body mass	0.004 (0.932)	-0.130 (0.003)
Body mass index	-0.004 (0.929)	-0.100 (0.023)

## A14 Differences among the sports science students with low and high entrepreneurial intentions: The case study of University of Novi Sad

### Radenko Matić, Nebojša Maksimović, Ivana Milovanović, and Brigita Banjac

#### Faculty of Sport and Physical Education, University of Novi Sad, Novi Sad, Serbia

##### **Correspondence:** Radenko Matić (radenko.matic@fsfvns.edu.rs)

*BMC Proceedings 2024*, **18(11)**: A14

**Background**

Due to the economic recession and significant increase in unemployment in the context of the recent international economic crisis, as well as the crisis caused by the COVID-19 pandemic, and also armed conflicts in Ukraine, among researchers there is a renewed interest in the role of entrepreneurship and its determinants [1, 2. 3]. The sports industry ecosystem, as a fertile ground for entrepreneurial opportunities, is a vital focus of this research, which aims to determine the entrepreneurial intentions (EI) of sports science students (SSS).

**Materials and methods**

Our study was conducted among bachelor's students at the Faculty of Sport and Physical Education, University of Novi Sad, Serbia (*N* = 571, 390 males and 181 females). The data collected in 2022 using a validated questionnaire which was statistically interpreted through path analysis (entrepreneurial skills (ES) as an independent dimension, attitude toward behavior (ATB), subjective norm (SN), perceived behavioral control (PBC) as mediators, and EI as a dependent dimension.

**Results**

The results are noteworthy for students with high EI, with a significant positive impact on ATB to EI in both genders (β = 0.54, males, β = 0.69, female students). Other latent dimensions (SN and PBC) do not have these impacts on EI for both genders.

**Conclusions**

This result implies that ATB is a significant and dominant predictor of the development of entrepreneurial intentions. This study can help recognize SSS as having high potential for future sports entrepreneurship, providing valuable insights for educational institutions and policymakers alike.

**Acknowledgment**

The manuscript was part of the Faculty of Sport and Physical Education project (Reg. No. 142-451-3459/2023-01), financed by the Provincial Secretariat for Higher Education and Scientific Research.

**References**

1. Huertas González Serrano M, Valantine I, Matić R, Milovanović I, Sushko R, Calabuig F. Determinants of entrepreneurial intentions in European sports science students: Towards the development of future sports entrepreneurs. Eur. Res. Manag. Bus. Econ. 2023; 29: 100229.
2. Stelmach M, Paár D, Matić, R. International co-operation of higher education institutions for the innovation and exchange of good practices in sport management: the MOSMEN educational project. Health Probl. Civiliz. 2023; 17:103-105.
3. Matic RM, González Serrano MH, Damjanović J, Maksimovic B, Papić-Blagojević N, Milošević I, Vuković J. Professional competencies development of sports science students: the need for more entrepreneurship education. Mark. Manag. 2022; 17:426-448.

## A15 Differences in body composition of professional football players according to their playing positions

### Dejan Javorac^1^, Jelena Slankamenac^1^, Saša Marković^2^, Slavko Molnar^1^

#### ^1^Faculty of Sport and Physical Education, University of Novi Sad, Novi Sad, Serbia; ^2^Faculty of Sport and Physical Education, University of Banja Luka, Banja Luka, Bosnia and Herzegovina

##### **Correspondence:** Dejan Javorac (dejan.javorac@fsfvns.edu.rs)

*BMC Proceedings 2024*, **18(11)**: A15

**Background**

In football training, players are subjected to technical-tactical activities according to their playing position [1]. Optimum body mass may impact activities in the game during the match, mainly in terms of ballistic efforts, i.e., jumps, acceleration, changes of direction, and thus sports performances. Determining and analyzing body composition in football is essential for optimizing performance and preserving players' health [2,3]. This research presents professional football players and their anthropometric properties by specialization of their playing positions.

**Materials and methods**

The sample comprised 46 professional football players (7 goalkeepers, 15 defenders, 17 midfielders, and 7 attackers) from the First Serbian League (aged 24.63±4.73) to research differences in body composition according to their playing positions. A bioelectrical impedance type Omron BF 511, Kyoto, Japan, was used to measure body composition. The first step involved analyzing the normality of the data distribution using the Kolmogorov Spearman test and calculating basic descriptive statistics. Multivariate analysis of variance (MANOVA) was used for statistical analysis, with a significance level set at p≤0.05.

**Results**

Professional football players from the First Serbian League are 183.7±5.6 cm tall; have body mass of 78.72±7.9 kg; body mass index 23.4±1.8 kg/m^2^; fat mass 16.1±3.1%; muscle mass 41.7±2.2 %; visceral fat 4.9±1.5 %; and basal metabolic rate 1767.1±96.6 kcal. Based on the results, there are no statistically significant differences in body composition according to playing positions among professional football players (F=1.25, *p*=0.21).

**Conclusions**

Knowledge of body composition can help coaches optimize players' performances. Regular measurement and body composition monitoring allow players and coaches to monitor progress. Football players who understand their body composition have greater awareness of their physical needs and health risks. Education on body composition can contribute to better health and long-term athletic career success. The horizontal distribution of playing positions contributed to these results. Future research should also focus on the vertical division of playing positions.

**References**

1. Cavia M, Moreno A, Fernández-Trabanco B, Carrillo C, Alonso-Torre S. Anthropometric characteristics and somatotype of professional soccer players by position. J Sport Med Ther. 2019;4:73–80.
2. Sebastiá-Rico J, Martínez-Sanz JM, González-Gálvez N, Soriano JM. Differences in body composition between playing positions in men’s professional soccer: A systematic review with meta-analysis. Appl Sci. 2023;13:4782.
3. Sutton L, Scott M, Wallace J, Reilly T. Body composition of English Premier League soccer players: influence of playing position, international status, and ethnicity. J Sports Sci. 2009;27(10):1019-1026.

**Table 1 (abstract A15).** Differences between playing positions

Variable	Goalkeepers (*N* = 7)	Defenders (*N* = 7)	Midfielders (*N* = 15)	Attackers (*N* = 17)		
	AS±SD	AS±SD	AS±SD	AS±SD	f	p
Height (cm)	187.57±7.41	184.57±5.06	184.13±5.82	181.35±4.14	2.351	0.086
Weight (kg)	80.93±9.69	82.69±5.84	78.10±7.67	76.74±7.90	1.174	0.331
Body mass index (kg/m^2^)	23.94±2.82	24.13±1.86	22.99±1.10	23.31±1.92	0.820	0.490
Fat mass (%)	16.76±2.39	16.99±1.92	14.87±2.92	16.66±3.76	1.274	0.296
Muscle mass (%)	41.19±2.20	40.87±1.33	42.53±1.97	41.44±2.63	1.235	0.309
Visceral fat (%)	4.86±1.57	5.43±1.51	4.40±1.12	5.06±1.64	0.959	0.421
Basal metabolic rate (kcal)	1803.29±118.32	1806.57±89.38	1765.60±82.79	1737.18±98.83	1.287	0.291
		F=1.002	*P*=0.468			

## A16 Mental health and satisfaction with life of good and poor sleepers

### Ivana Duvnjak, Iva Šklempe Kokić

#### Faculty of Kinesiology, “Josip Juraj Strosssmayer” University of Osijek, Osijek, Croatia

##### **Correspondence:** Ivana Duvnjak (iduvnjak@kifos.hr)

*BMC Proceedings 2024*, **18(11)**: A16

**Background**

The concept of sleep represents an essential biological need in human life, especially in periods of great mental and physical fatigue. To determine the type of sleep, it is important to consider both quantity and quality of sleep. Good sleep is related to stable mood, concentration and academic achievement. Poor sleep is related to physical aspects of health, like chronic pain, hypertension, and higher body mass index [1]. However, there are also undesirable psychological consequences due to poor sleep quality. Some findings show the emergence of anxiety, aggression, attention deficit and generally poorer mental health [2]. This study aims to examine the differences in life satisfaction and mental health of good and poor sleepers.

**Materials and methods**

The sample comprised 221 participants with a mean age of 22 years (M = 21.9, SD = 1.9). Participants were undergraduate and graduate kinesiology students who completed The Pittsburgh Sleep Quality Index, The 36-item Short-Form Health Survey and the Satisfaction with Life Scale.

**Results**

The global score of the Pittsburgh Sleep Quality Index, whereby a higher score represents poorer sleep quality, measured sleep quality. Considering the cut-off global score participants were divided into good sleepers and poor sleepers [3]. The obtained results show that 52% of students have poor quality of sleep. A third of the students considered their subjective sleep quality to be very good, and 25.8% obtained a score of 0 for sleep latency. The sleep duration factor shows that 41.6% of students slept more than 7 hours every day (about 75% in other research on student population), and 75.1% obtained a sleep efficiency rating of more than 85% using the formula actual sleep time/actual time in bed. None of students scored 19 or more for sleep disorders, and 5% used sleep medicines (about 10% in other studies on students). By comparing means of good and poor sleepers, it was found that there are significant differences in all of the measured components of mental health and satisfaction with life (Table 1). Students who have good sleep quality have fewer role limitations due to emotional problems, greater vitality, higher levels of emotional well-being, and better social functioning. Furthermore, good sleepers are significantly more satisfied with their life.

**Conclusions**

The obtained results emphasize the effect of good quality of sleep on various components of mental health and general satisfaction with life in the student sample.

**References**

1. Clement-Carbonell V, Portilla-Tamarit I, Rubio-Aparicio M, Madrid-Valero JJ. Sleep quality, mental and physical health: A differential relationship. Int J Environ Res Public Health. 2021;18(2):460. https://doi.org/10.3390/ijerph18020460
2. Franquelo-Morales P, Sánchez-López M, Notario-Pacheco B, Miota-Ibarra J, Lahoz-García N, Gómez-Marcos MÁ, Martínez-Vizcaíno V. Association between health-related quality of life, obesity, fitness, and sleep quality in young adults: the Cuenca adult study. Behav Sleep Med. 2018;16(4):347-355. https://doi.org/10.1080/15402002.2016.1228638
3. Buysse DJ, Reynolds CF III, Monk TH, Berman SR, Kupfer DJ. The Pittsburgh Sleep Quality Index: A new instrument for psychiatric practice and research. Psychiatry Res. 1989;28(2):193-213. 10.1016/0165-1781(89)90047-4

**Table 1 (abstract A16).** Differences in mental health and satisfaction with life in relation to sleep quality

Variable	Good sleepers (*N* = 104)	Poor sleepers (*N* = 117)			
	M±SD	M±SD	t	p	Cohen’s d
SF-36 Health Survey					
Role limitations due to emotional problems	85.26±30.39	72.93±37.12	2.68	*p* < 0.01	0.36
Vitality/Energy	69.95±14.38	58.76±14.83	5.68	*p* < 0.01	0.77
Emotional well-being	80.35±12.69	69.05±14.96	6.01	*p* < 0.01	0.81
Social functioning	84.01±20.12	74.46±20.32	3.50	*p* < 0.01	0.47
Satisfaction with life	5.57±1.14	4.93±1.14	4.11	*p* < 0.01	0.56

## A17 Does electromyographic biofeedback-assisted exercise improve outcomes in patients after total hip and knee arthroplasty? Secondary analysis of data from two randomized controlled trials

### Iva Šklempe Kokić^1^, Matko Vuksanić^2^, Tomislav Kokić^1,3^

#### ^1^Faculty of Kinesiology, “Josip Juraj Strossmayer” University of Osijek, Osijek, Croatia; ^2^Bizovačke Toplice Rehabilitation Hospital, Bizovac, Croatia; ^3^Faculty of Medicine, “Josip Juraj Strosssmayer” University of Osijek, Osijek, Croatia;

##### **Correspondence:** Iva Šklempe Kokić (iva.sklempe.kokic@kifos.hr)

*BMC Proceedings 2024*, **18(11)**: A17

**Background**

While the incidence of total hip and knee replacements is increasing worldwide there is still a significant number of patients with suboptimal results, and possibilities for the improvement of outcomes. This study aimed to investigate the effect of electromyographic biofeedback (EMG-BF) in rehabilitation after total hip (THA) and knee arthroplasty (TKA) on self-perceived health, pain levels and functional outcomes.

**Materials and methods**

This is a secondary analysis of data from two randomized controlled trials that examined the effects of EMG-BF on patients after THA and TKA (ACTRN12622001130752; ACTRN12618001782224) [1, 2]. A total of 200 patients were included, 117 after TKA, and 83 after THA. Participants were assigned to 2 groups: an experimental group (EG) (*n*=100; mean age 66.5 ± 8.2; 59% TKA, 42% males), and a control group (*n*=100; mean age 66.9 ± 7.9; 58% TKA; 49% males). All patients participated in 21 days of rehabilitation which included therapeutic exercise, electrotherapy, and education. EG received EMG-BF during a portion of therapeutic exercise. The subscale Physical Function of The Western Ontario and McMaster Universities Arthritis Index (WOMAC-PF), numeric rating scale (NRS), use of mobility aid, 30s Chair Stand Test (CST), Timed Up & Go test (TUG), and visual analogue scale of the EQ-5D-5L (EQ VAS) questionnaire were used to measure outcomes.

**Results**

A higher proportion of the participants in both groups did not need mobility aid after the rehabilitation (*p* < 0.001). Likewise, WOMAC-PF, NRS, 30s CST, TUG and EQ VAS scores significantly improved (*p* < 0.001) between baseline and final values. No significant differences between the groups were found except EQ VAS score which was higher in the EG after the intervention (*p* = 0.008) (Table 1). A two-way ANOVA was performed to evaluate the effects of surgery and group on outcomes. The results indicated significant main effect for surgery, F(1) = 8.8, *p* = 0.003, partial η2 = 0.04; significant main effect for group, F(1) = 7, *p* = 0.009, partial η2 = 0.04; and no significant interaction between surgery and group on EQ VAS after the rehabilitation.

**Conclusions**

EMG-BF did not provide additional benefits to the conventional rehabilitation after THA and TKA, except for the self-perception of health status.

**References**

1. Kokic T, Pavic R, Vuksanic M, Jelica S, Sumanovac A, Banic T, Ostović H, Sklempe Kokic I. Effects of electromyographic biofeedback-assisted exercise on functional recovery and quality of life in patients after total hip arthroplasty: A randomized controlled trial. J Pers Med. 2023;13(12):1716. 10.3390/jpm13121716
2. Sklempe Kokic I, Vuksanic M, Kokic T, Peric I, Duvnjak I. Effects of electromyographic biofeedback on functional recovery of patients two months after total knee arthroplasty: A randomized controlled trial. J Clin Med. 2022;11(11):3182. 10.3390/jcm11113182

**Table 1 (abstract A17).** Outcomes after the intervention – between-group analyses

Outcome measure	EG (N = 100)	CG (N = 100)	p
Use of mobility aid (N(%))			
Unilateral crutch	38(38)	36(36)	0.639
Two crutches	6(6)	9(9)	
Walker	0(0)	1(1)	
No walking aid	56(56)	54(54)	
WOMAC-PF (range 0-68; mean (SD))	8.6(8.1)	10.4(8.3)	0.128
NRS (0-10 scale; mean (SD))	0.9(1.3)	1.2(1.5)	0.155
30s CST (no. of stands; mean (SD))	13.3(3.8)	12.4(4.1)	0.118
TUG (seconds; mean (SD))	10.2(3.3)	10.8(3.9)	0.236
EQ-VAS (range 0-100; mean (SD))	83.4(12.7)	78.3(14.3)	0.008*

WOMAC-PF - Physical Function of The Western Ontario and McMaster Universities Arthritis Index; NRS – Numeric Rating Scale; 30s CST - 30s Chair Stand Test; TUG - Timed Up & Go test; EQ-VAS - Visual Analogue Scale of the EQ-5D-5L; *statistically significant

## A18 Students' perception of the influence in coordination abilities of physical activities on the development of an active process of self-regulated learning

### Gordan Drašinac^1^, Mirela Müller^2^, Damjan Jakšić^3^

#### ^1^Department of Professional Studies, University of Split, Split, Croatia; ^2^Faculty of Humanities and Social Sciences, University of Split, Split, Croatia; ^3^Faculty of Sport and Physical Education, University of Novi Sad, Novi Sad, Serbia

##### **Correspondence:** Gordan Drašinac (gordan.drasinac@oss.unist.hr)

*BMC Proceedings 2024*, **18(11)**: A18

**Background**

Contemporary, recent research implicitly indicates the importance of coordination abilities that can positively affect the learning process. While there are various scientific studies on the relationship between motor abilities and cognition, there is a lack of research regarding the influence of certain degrees of coordination abilities on the development of a self-regulated learning process. Given that the effect of different analyzers can have a decisive influence on the quality of coordination abilities, the subject of this paper will be the examination of students' perceptions of the degree of connection of motor or sports abilities to the development of the process of self-regulated learning. The paper starts from the main hypothesis of the research, whereby, by examining the student's perception, we will try to point out the importance of the connection between the degree of development of the coordination abilities of physical activities and the development of self-regulated learning.

**Materials and methods**

The online survey was conducted in the period from May 25 to June 15, 2023, among students of two faculties in Split and one from Novi Sad.

**Results**

The paper tries to point out some of the teaching strategies that are used in physical activities and practical exercises as part of regular higher education classes, which can encourage self-directed learning of movement and thus develop parallel degrees of coordination ability.

**Conclusions**

The research indicates possible differences and/or correlations among students with regard to dependent and independent variables.

## A19 Effects of combined exercise program on happiness and life satisfaction on physically active older adults: Systematic review

### Romina Herodek^1^, Mladen Živković^1^, Aleksandra Ilić^2^, Katarina Herodek^1^, and Aleksandra Catić Đorđević^2^

#### ^1^Faculty of Sport and Physical Education, University of Niš, Niš, Serbia; ^2^Faculty of Medicine, University of Niš, Niš, Serbia

##### **Correspondence:** Romina Herodek (romina.fsfv@gmail.com)

*BMC Proceedings 2024*, **18(11)**: A19

**Background**

Well-being is the assessment of individual experiences with pleasant feelings like happiness, joy, and life satisfaction. Combined training has been suggested for older adults to decrease age-related psychological changes and functional limitations.

**Materials and methods**

This systematic review was conducted to determine the effects of a combined exercise program on happiness and life satisfaction in physically active older adults. The electronic databases of Google Scholar, Web of Science, and PubMed were searched between 2000 and 2023 for available literature. The searched keywords were: older adults, training, exercises, happiness, and life satisfaction. After completing the investigation, 931 publications were found. Criteria for inclusion in further analysis were that the samples included physically active participants over 60 years old and that the studies were written in English. Studies in which participants had obesity, diabetes, and other non-communicable diseases were excluded from investigation. Finally, the qualitative analysis involved a total of eight studies. These studies ranged in publication date from 2008 to 2022.

**Results**

The participants in the studies were men and women aged from 60 to 79 years. The sample size was 1849 and ranged from 35 to 656 participants. Physical activity was a combination of aerobic training, strength training, stretching exercises, and balance exercises. The most commonly used questionnaire was the Satisfaction with Life Scale (SWLS). The findings have demonstrated that older adults of both genders who underwent combined training programs had higher levels of happiness and life satisfaction.

**Conclusions**

Research has shown that leading an active lifestyle enhances older persons' happiness and satisfaction with life. These results support the importance of maintaining an active lifestyle to improve the mental and emotional well-being of older adults.

## A20 Effects of resistance training on muscle potential in late adolescents

### Oliver Radenković^1^, Dušan Stanković^2^, Emilija Petković^2^, Nikola Aksović^3^, Saša Bubanj^2^

#### ^1^Department of Biochemical Science and Sport, State University of Novi Pazar, Novi Pazar, Serbia; ^2^Faculty of Sport and Physical Education, University of Niš, Niš, Serbia; ^3^Faculty of Sport and Physical Education, University of Priština, Leposavić, Serbia

##### **Correspondence:** Saša Bubanj (bubanjsale@gmail.com)

*BMC Proceedings 2024*, **18(11)**: A20

**Background**

The popularity of resistance training has surged, extending beyond bodybuilders to include adolescents, adults, seniors, and clinical populations. However, most research has focused on males, necessitating an examination of how females respond to resistance training. This study aimed to investigate the impact of resistance training on muscle potential in late adolescents/students.

**Materials and methods**

The sample comprised 127 students from the Department of Biomedical Sciences, State University of Novi Pazar, aged 20±1 years. An 8-week resistance training program was conducted, with participants training twice weekly. They were divided into an experimental group (*n*=87, including 39 males and 48 females) and a control group (*n*=40, including 24 males and 16 females). The experimental group followed the training, while the control group maintained their usual activities.

Muscle potential was assessed using the Myotest system, measuring explosive strength via bench press and squat tests. Parameters used for evaluating explosive strength included Power (W), Maximum Power (W), Force (N), and Velocity (cm/s). Statistical analysis involved descriptive measures, univariate and multivariate analysis of variance (ANOVA, MANOVA), and tests for normality.

**Results**

Results showed significant improvements in lower extremity explosive strength for all groups after training. For explosive strength of the upper extremities in the experimental group of males, there were no statistically significant changes at the multivariate level (*p*=0.146; *p*>.05). However, the experimental group of females demonstrated significant changes in explosive strength (*p*=.000; *p*<.05). This suggests that females respond more favorably to resistance training.

**Conclusions**

In conclusion, the study supports the safety and effectiveness of resistance training for late adolescents of both genders. It also emphasizes the need for more research involving female participants to dispel concerns about muscle hypertrophy. These findings advocate for the inclusion of resistance training in exercise programs for late adolescents, regardless of gender.

## A21 The sporting migrants and naturalization trajectory in red China

### Yiyong Liang, Wei Zhao

#### Hebei Sport University, Shijiazhuang, Hebei, China

##### **Correspondence:** Yiyong Liang (y.liang@hepec.edu.cn)

*BMC Proceedings 2024*, **18(11)**: A21

**Background**

The Chinese government openly adopted an approach that to naturalize athletes from abroad and to employ foreign sport talents in order to achieve a status of a global sport powerhouse and strengthening its international influence.

**Materials and methods**

This article employs the theoretical framework of Push-Pull Migration Law and World-system theory, it uses premier data from total 10 semi-structured interviews (five senior Chinese sport administrators - from the State Sport General Administration and five from relevant athletes and coaches); secondary data (focused on state mainstream media platforms, such as China Central Television, China Daily newspaper, sohu.com, State Council homepage and so on) to analyze the impact of China’s political and sport policies on its athletes’ international movement since the establishment of communist rule in 1949.

**Results**

In terms of Chinese sport development, authors saw the migration pattern has been evolving and showing different features which are affected by the push-pull factors of various dimensions at different times. Moreover, the two-way directions movement and even Chinese citizenship can be acquired through the ‘jus talenti’ model based on athletes’ talent rather than the ‘jus sanguinis’ model based on bloodline and heritage has been consolidated and such a trend is likely to continue in the foreseeable future.

**Conclusions**

The finding shows pragmatism of the Chinese state governance practice and policies over its sport development and the overall historical trajectory of sport talents movement. Indeed, it is two sides of the same coin, the big controversial issue created recently demonstrated that the government wants to keep enforcing the current Nationality Law by denying the dual-nationality chances of the ordinary people and at the same time it attempts to attract global talents for its projects of ‘China Dream’ by flagrantly violating its own law for the convenience of elite few. The article draws an evolutionary pattern of migrates movement in Chinese sport sector to provide a different angle for gaining a better understanding of sport development in red China.

## A22 Physical activity and physical functions of older adults residing in the community

### Maria Justine, Adrina Abd Rahim

#### Centre for Physiotherapy Studies, Faculty of Health Sciences, University Teknologi MARA Selangor, Puncak Alam Campus, Puncak Alam, Selangor Malaysia

##### **Correspondence:** Maria Justine (maria205@uitm.edu.my)

*BMC Proceedings 2024*, **18(11)**: A22

**Background**

Ageing presents multifaceted physiological challenges, exacerbating the need for physical activity (PA) to counteract declining physical functions. Despite extensive literature on PA and its association with quality of life, the interplay between modifiable factors like functional performance, muscle strength, exercise tolerance, and physical activity remains underexplored, especially in the Malaysian context. Hence, this study aimed to evaluate the physical functions of community-dwelling older adults and to ascertain how it relate to their PA levels.

**Materials and methods**

A cross-sectional study was conducted among community-dwelling older adults (*N*=241) in selected areas of Malaysia. Data collection encompassed sociodemographic details, clinical history, PA levels (low, moderate, high) using the Physical Activity Scale for the Elderly (PASE), anthropometric and body composition measurements, and various physical function tests (muscle strength, balance and mobility, exercise tolerance). The study employed the SPSS Version 26.0 for descriptive and inferential statistical analyses.

**Results**

The majority of participants were females (64.7%), predominantly Malays. Approximately 43% reported being inactive previously. Significant variations were observed in body composition, anthropometrics, and physical functions across PA levels. Notably, calf circumference differed significantly between low and high PA groups (*p*=0.028). Handgrip strength and lower limb strength showed pronounced variations across PA categories (*p*<0.001). Correlation analysis revealed a positive association between PA and muscle mass (*r*=0.214, *p*=0.001) and exercise tolerance (*r*=0.365, *p*<0.001). Conversely, balance and mobility displayed a negative correlation with PA (*r*=-0.239, *p*<0.001).

**Conclusions**

This study underscores the critical role of PA in mitigating age-related declines in physical functions among Malaysian older adults. The findings emphasize the potential benefits of targeted PA interventions, focusing on muscle strength and balance enhancement. Future research should explore tailored exercise regimens that address the unique challenges faced by older adults in Malaysia, promoting active and healthy ageing. (Funding: GERAN DANA UITM SELANGOR (DUCS Faculty: 600 UiTMSEL (PI. 5/4) (151/2022)).

## A23 The impact of an educational intervention on the physical activity of adolescents during the Covid-19 pandemic

### Mirela Šunda^1^, Barbara Gilić Škugor^2^, and Nataša Zenić^2^

#### ^1^Faculty of Kinesiology, “Josip Juraj Strossmayer” University of Osijek, Osijek, Croatia; ^2^Faculty of Kinesiology Split, University of Split, Split, Croatia

##### **Correspondence:** Mirela Šunda (sundamirela@gmail.com)

*BMC Proceedings 2024*, **18(11)**: A23

**Background**

A significant number of adolescents do not meet the recommended guidelines for physical activity and the period of adolescence is characterized by a significant decline in the level of physical activity. Physical literacy is considered as one of the key concepts for quality educational and sports programs aimed at promoting physical activity and health. Therefore, the aim of this study was to determine the effectiveness of the originally developed distance learning program in terms of the quantitative increase in physical activity of adolescents.

**Materials and methods**

The study included 544 (365 female, 179 male) adolescents aged 14 and 18, divided into intervention and control groups. For a period of 12 weeks, the intervention group received animated video materials intended to increase physical activity and physical literacy. Variables included Physical activity questionnaire for adolescents (PAQ-A) and demographic characteristics. The effects of the intervention were calculated using 2x2 (group x time) analysis of variance.

**Results**

There were no interaction effects for the PAQ-A questionnaire in the total sample (F-value= 1.17, *p*= 0.28.001, Effect size= 0.003) which is explained by the fact that the final testing was conducted at the end of the school holidays, which was conditioned by the COVID-19 pandemic.

**Conclusions**

The study gave a clear picture that in the Physical education curriculum in Croatia has been neglected the part related to the Physical literacy especially with theoretical knowledge about the importance and health benefits of physical activity.

## A24 What predicts flow experience in Serbian professional athletes? The role of motivation and psychobiosocial state

### Jovana Trbojević Jocić^1^, Jelica Petrović^2^

#### ^1^Study Program of Psychology, University of Kragujevac, Kragujevac, Serbia; ^2^Department of Psychology, Faculty of Philosophy, University of Novi Sad, Novi Sad, Serbia

##### **Correspondence:** Jovana Trbojević Jocić (jovana.trbojevic88@gmail.com)

*BMC Proceedings 2024*, **18(11)**: A24

**Background**

Flow is an internal, multidimensional state of optimal activation that has been shown to have positive relations to sports achievement, confidence, motivation, automaticity, improvement of skills, and psychological wellbeing. Individual Zones of Optimal Functioning (IZOF) model proposes that psychobiosocial states such as emotions, are related to sport performance, and can have functional or dysfunctional impact on sport experience such as flow. Having in mind that flow is composed of cognitive, physiological, and affective factors, the goal of this research is to examine predictive role of affective (psychobiosocial) and motivational factors on flow experience in professional athletes in Serbia.

**Materials and methods**

The sample consisted of 112 athletes (female 66.1%, Mage = 22.35, SD = 5.67), who play individual and collective sport in average of 11.80 years in Serbia. Questionnaires Motives for Physical Activities Measure – Revised (MPAM-R, Ryan et al., 1997); Core Flow (Jackson, 1996), and The Psychobiosocial States Scale (PBS-S, Ruiz et al., 2018) were applied.

**Results**

Two steps hierarchical linear regression analysis was applied. First block of five motives for physical activities (enjoyment, competence, appearance, fitness, social) is statistically significant (F(5,106) = 10.14, *p* = .001) and explains 32.4% of variance. In the second block psychobiosocial states (functional and dysfunctional) were entered too and this model represent significant predictive model of flow in athletes (F(2,104) = 14.52, *p* = 0.001) and explain 49.5% of variance. Motive Competence (β = 0.266, *p* = 0.019) and functional psychobiosocial state (β = 0.449, *p* = 0.001) are significant individual predictors of flow.

**Conclusions**

Athletes who train sports to gain competence, develop skills, and are in general task-oriented and have functional psychobiosocial state such as feeling of pleasure, happiness, and sense of meaning, are more likely to experience flow in sports.

## A25 Evaluation the amount of classroom physical activity integration into mathematics lessons

### Urška Čeklić

#### Faculty of Health Sciences, University of Primorska, Izola, Slovenia

##### **Correspondence:** Urška Čeklić (urska.ceklic@fvz.upr.si)

*BMC Proceedings 2024*, **18(11)**: A25

**Background**

Many studies have shown that incorporating physical activity (PA) into teaching and learning process has yielded positive academic outcomes, especially in mathematics. In addition, it is also a possible solution to reduce sedentary behavior and increase the amount of PA. The main objective of this study is to find out whether integrating PA into maths lessons can increase the amount of PA and improve academic outcomes.

**Materials and methods**

A total of 40 third-grade students from a elementary school in the Coast region were included in this pilot study. The experimental group (EG) consisted of 23 students (12 males and 11 females) and 17 similar-aged control peers (8 males and 9 females). These participants wore accelerometers for five consecutive days during the entire class period from 8.20 am to 12.55 pm. The exclusion criteria were time worn and not attend to maths exam. In the EG, the goal of each maths lesson was to learn units in an active way and to be as active as possible. We observed differences between groups in the amount of MVPA and percentage achievement in maths grades. Most of the observed variables were normally distributed.

**Results**

It can be concluded that after the intervention, the percentage of maths grades was statistically significantly higher in the EG than in the CG (*p* = < 0.001; ES = 0.83 and 1.79, respectively). In addition, the EG spent statistically significantly more time on MVPA variables (min and %) (*p* < 0.05) and increased CPM on Monday and Wednesday (*p* < 0.001) compared to the CG.

**Conclusions**

The intervention has shown that integrating PA into maths lessons increases academic achievement in maths and increases the amount of PA during the school days. A pilot study may serve for future investigation in the area of academic achievement in active learning.

## A26 Personality traits, motivation and self-concept as predictors of attitudes towards the teaching of physical education

### Andrea Keser

#### ES “Miloš Crnjanski” Potočani, Prnjavor, Bosnia and Herzegovina

##### **Correspondence:** Andrea Keser (andrea.stankovic27@gmail.com)

*BMC Proceedings 2024*, **18(11)**: A26

**Background**

Previous research in the field of psychology and medicine suggests that there is a trend of increasing negative attitudes towards physical education classes among high school students, which manifests itself through less participation in physical activities, more frequent absences and more frequent requests for exemption from physical education classes.

**Materials and methods**

The subject of the research was the influence of personality traits, motivation and self-concept on attitudes towards PE teaching. In total, 250 second and third grade high school students participated in the research. BFI, SRQ, SPQ and PEAS scales were used as instruments with appropriate levels of reliability (ranging from 0.706 to 0.936 Chronbach-alpha). In addition, the moderating influence of gender on the relationship between motivation and self-concept (as independent variables) and attitudes towards PE teaching (as dependent variable) was tested.

**Results**

The obtained results confirmed a strong positive correlation between the personality trait Conscientiousness and dimensions of motivation characterized by a high degree of self-regulation (Intrinsic motivation and Identified regulation) and attitudes toward PE. A positive correlation of moderate and lower intensity was determined for the relationship between the dimensions Extraversion, Agreeableness and Openness and attitudes towards PE teaching. A negative high correlation was established between the dimensions of Amotivation and attitudes towards PE teaching, and a negative correlation of moderate and low intensity was established for the dimensions of External regulation, Neuroticism and General self-concept. The moderating influence of gender on the relationship between motivation and self-concept and attitudes towards PE teaching was not been confirmed.

**Conclusions**

The conducted research gives a clearer picture of the mutual relationship between the psychological characteristics and the physical activity of students.

## A27 Older football players exhibit significant eccentric hamstring strength decreases following a football match-play

### Ersagun Kepir^1^, Gokhan Mehmet Karatay^2^, Bekir Car^3^, Esedullah Akaras^4^, Necmiye Un Yildirim^5^, Serdar Eler^6^, Gokhan Yagiz^7,8^, Julian Owen^1^

#### ^1^Institute for Applied Human Physiology, School of Psychology and Sport Science, Bangor University, Bangor, United Kingdom; ^2^Department of Physiotherapy and Rehabilitation, Faculty of Health Sciences, Gazi University, Ankara, Türkiye; ^3^Faculty of Sport Sciences, Bandirma Onyedi Eylul University, Balikesir, Türkiye; ^4^Department of Physiotherapy and Rehabilitation, Faculty of Health Sciences, Erzurum Technical University, Erzurum, Türkiye; ^5^Faculty of Physiotherapy and Rehabilitation, Health Sciences University, Ankara, Türkiye; ^6^Faculty of Sport Sciences, Gazi University, Ankara, Türkiye; ^7^Department of Kinesiology, College of Health and Human Performance, East Carolina University, Greenville, North Carolina, United States of America; ^8^Department of Physiotherapy and Rehabilitation, Faculty of Health Sciences, Amasya University, Amasya, Türkiye

##### **Correspondence:** Ersagun Kepir (pepa37@bangor.ac.uk)

*BMC Proceedings 2024*, **18(11)**: A27

**Background**

Hamstring strain injuries (HSI) constitute 24% of all injuries in football. HSI often happen close to the ends of the halves of a football match due to possible fatigue-leading causation. It is well-defined in the literature that the risk of HSI also increase with age. Hence, the present study aimed to compare the effects of football-specific fatigue on eccentric hamstring strength during a football match between older and younger professional football players.

**Materials and methods**

Changes in perceived muscle soreness and maximal eccentric hamstring strength were investigated immediately after a football match in younger (*n*=11, aged between 19 and 22 years) and older (*n*=10, aged 23 and over years) male football players. Before the study participation, participants read and signed a written informed consent according to the Declaration of Helsinki.

**Results**

Older players showed a significant decrease in their maximal eccentric hamstring strength (-20 N ± 38.5, *p* = 0.027), while there were no significant decrements in the younger players (-3 N ± 36.1, *p* = 0.773). Both the older (29 % ± 14.1, *p* < 0.001) and younger groups (40%, *p* < 0.001) declared significant increments in the perceived muscle soreness of the hamstring muscles. Younger players reported significantly higher soreness in their hamstrings in comparison to older players (11% ± 17.2, *p* = 0.025).

**Conclusions**

Older players showed a significant decrease in their maximal eccentric hamstring strength immediately after a football match. Sports professionals should focus on enhancing the hamstring eccentric strength sustainability in older players during a football match-play.

## A28 Changes in physical literacy levels among high school adolescents following a 12-week educational intervention

### Barbara Gilić^1^, Mirela Šunda^2^, Damir Sekulić^1^

#### ^1^Faculty of Kinesiology, University of Split, Split, Croatia; ^2^Faculty of Kinesiology, “Josip Juraj Strossmayer” University of Osijek, Osijek, Croatia

##### **Correspondence:** Barbara Gilić (barbaragilic@gmail.com)

*BMC Proceedings 2024*, **18(11)**: A28

**Background**

Physical literacy (PL) is an important concept for maintaining optimal levels of physical activity during the life course and is important among adolescents who are in the developmental life stage. This research aimed to investigate the effectiveness of educational materials applied as a part of physical education classes on the PL of Croatian adolescents.

**Materials and methods**

This research included 365 female and 179 male adolescents (544 in total) aged 14 to 18, who were divided into intervention and control groups. The intervention group received short video materials over 12 weeks intended to influence the improvement of PL and physical activity. Variables of this research included demographic characteristics and two PL questionnaires: Canadian Assessment of Physical Literacy Second Edition (CAPL-2) and the Physical Literacy Assessment of Youth (PLAYself). The effects of the intervention were calculated using a multifactorial analysis of variance (group x time).

**Results**

In the overall sample, there was a significant group x time interaction difference for PLAYself (F-value=11.3, *p*=0.001, ES=0.02), with the control group showing a bigger improvement and the intervention group slightly reducing the PLAYself score. In the CAPL-2 questionnaire, there were no interaction effects.

**Conclusions**

The findings of this study can be explained by the fact that the intervention group initially had significantly higher PLAYself scores than the control group; therefore, their development was naturally restricted. Furthermore, because PLAYself is a self-assessment instrument, students' perceptions of their abilities and self-efficacy for engaging in physical activity likely changed as a consequence of the intervention. Future intervention studies should look at different age groups (e.g., children and adults) and examine different PL dimensions, including physical competence and confidence.

## A29 Solution for medical rehabilitation by remote monitoring the patient’s implementation of exercises set

### Matvey Sevastyanov^1^, Ivan Chavychelov^1^, Aleksandr Khelvas^2^, Roman Pavlovich^2^

#### ^1^MIPT, Dolgoprudnii, Russia; ^2^AtFrame DOO, Novi Sad, Serbia

##### **Correspondence:** Roman Pavlovich (hel@cos.ru)

*BMC Proceedings 2024*, **18(11)**: A29

**Background**

The main goal of the project is to create a unified technological platform for medical rehabilitation by monitoring the implementation of sets of exercises remotely. In the proposed case, the user registers on the system portal and gets access to methodological booklet and video materials for performing a set of exercises. The patient, depending on the prescribed rehabilitation program, receives for a period of time (2-3 months) a software and hardware complex for monitoring the execution of the exercise set with high amplitude. The execution of exercise sets is performed with automatic control using the innovation system on the base of TOF (Time of Flight) camera. It is important that the patient’s video is not transferred to the cloud infrastructure. All processing is carried out on the side of the system located in the patient destination.

**Materials and methods**

Hardware base includes portal cloud solution for patient’s data processing and TOF camera with embedded GPU processing module on the client side. AtFrame software is used for TOF video processing. As a result, we get the number of repetitions and the metrics for correctness of the exercise. Also, we can estimate the progress of the rehabilitation process. Metrics and the vector model of exercise execution are transferred to the portal and used to improve exercise sets based on big data. Patient gets online voice commentary for the patient in online.

**Results**

As a result, for each session of exercises we get the realistic high-quality 3D animation and frame-by-frame deviation of each control point of the skeletal model from the exercise standard.

On the base of metrics set we generate voice recommendations for the correct execution of exercises by patient.

**Conclusions**

The results of our research lead us to the innovative rehabilitation solution for the third (home) stage of rehabilitation. Patient can get voice assistance on the base of AI LLM service.

## A30 Online soccer analytics and robotic video broadcasting system

### Aleksandr Khelvas, Roman Pavlovich

#### AtFrame DOO, Novi Sad, Serbia

##### **Correspondence:** Roman Pavlovich (hel@cos.ru)

*BMC Proceedings 2024*, **18(11)**: A30

**Background**

Generating statistics for each football match is an important part of the training process all over the world. It is impossible to imagine the work of the team staff that would not pay attention to these analytics.

**Materials and methods**

The key component of the platform is a set of high-resolution cameras, video equipment and special software for video recording and video broadcasting.

The solution provides AI-based video recording and broadcast of games with all the features inherent in football broadcasting-close-up and long-range shots, slow-motion and replays of key moments of the game. An important area of application of video analytics is the archive accumulation for each of the club players, from children’s teams to the winners of national and international competitions. Archives of video recordings of matches, fragments of games, and set of video analytics are maintained in all countries whose clubs are achieving international success. This opportunity allows clubs to search for young talents on the base of objective indicators. The third component of the project is built on the latest achievements of Large Language Model, which will allow to create automatic commentary on football matches that is almost indistinguishable from a human commentator. The basis for such a comment is the analytical log.

**Results**

The resulting solution creates the ecosystem, which will raise the issues of football video analytics, broadcasting and matches commentary to a new level. Implementation of the results will improve the level of football clubs, efficiency of the search for talented players, popularize football, and create new sources of income for the football ecosystem.

**Conclusions**

The results can be used in other sports (volleyball, handball etc.) It will lead to improve the results of athletes and teams and attract new fans. Online robotic commentary will increase the entertainment quality and profitability of sports competitions.

## A31 Two Olympic water polo national teams in Tokyo and the body composition of their players

### Jovan Gardašević, Ivan Vasiljević

#### Faculty for Sport and Physical Education, University of Montenegro, Nikšić, Montenegro

##### **Correspondence:** Jovan Gardašević (jovan@ucg.ac.me)

*BMC Proceedings 2024*, **18(11)**: A31

**Background**

The Olympic Games always offer great interest when it comes to water polo. Many selections have an approximate quality and details decide who will win. That is why the authors wanted to check the body composition and anthropometric characteristics before the tournament of players of the two national teams, USA and Montenegro. The purpose of this research was to determine the differences in body composition and anthropometric characteristics between the water polo players of the national team of USA, which was sixth at the Olympic Games in Tokyo 2021, and Montenegro, which was in eighth place.

**Materials and methods**

Body mass index, fat percentage and muscle mass were evaluated by Bioelectric Impedance type MC-980 and body height, body weight, triceps skinfold, biceps skinfold, skinfold of the back, abdominal skinfold, upper leg skinfold, and lower leg skinfold (other anthropometric characteristics) were evaluated by an anthropometer and caliper.

**Results**

The T-test showed statistically significant differences, it can be stated that water polo players from Montenegro (13.33 %), had a statistically significantly lower fat percentage than water polo players from the USA (16.67 %). Although the fat percentage is a disruptive factor with athletes, it had no effect on the result at the Olympic Games in Tokyo, because the USA obtained sixth place, and Montenegro eighth place.

**Conclusions**

Although USA won the sixth place at the Olympic Games in Tokyo, and Montenegro was in eighth place, it can be stated that there was a statistically significant difference in only one body composition variable (fat percentage) between water polo players of the USA and Montenegro. It means that some other abilities influenced the achievement of results at the Tokyo Olympics in water polo, for example tactical, psychological, technical etc., which is to be shown by some other research.

## A32 The effectiveness of a new and promising method for assessing the general physical condition of the adult population - European Fitness Badge

### Viorel Petru Ardelean^1^, Vlad Adrian Geantă^1^, Corina Dulceanu^1^, Claudiu Bulzan^1^, Denis Petran^1^, Vasile Emil Ursu^2^

#### ^1^Faculty of Physical Education and Sport, “Aurel Vlaicu” University of Arad, Arad, Romania; ^2^Department of Physical Education and Sport, Faculty of Law and Social Sciences, University “1 Decembrie 1918” of Alba Iulia, Alba Iulia, Romania

##### **Correspondence:** Viorel Petru Ardelean (viorel.ardelean@uav.ro)

*BMC Proceedings 2024*, **18(11)**: A32

**Background**

There are alarming statistics showing that in more than half of the EU countries, the level of weekly physical activity among adults is below 25%. The aim of this paper is to present a new comprehensive, objective test battery that has a number of advantages over other test batteries (wide applicability, the way of recording performance, automatic data processing, the creation of an personal profile for each participant).

**Materials and methods**

The EFB test battery provides individualized feedback on the fitness level of an adult (18 - 65 years), depending on the level of fitness of the subjects and taking into account certain motor skills such as (strength, endurance, speed, coordination) or some anthropometric measurements (height, weight, waist circumference, BMI, posture, ABSI). In this study participated students from Aurel Vlaicu University of Arad, enrolled in different study programs (e.g. engineering, economics, physical education and sport, psychology, etc.) *N* = 180 (M = 79; F = 101), with an average age: Mage = 23.59 ± 7.38.

**Results**

Following a physical activity questionnaire, the sample was divided into two groups according to the physical activity: basic (*N*=93) and advanced (*N*=87). Regarding the results obtained, we present here values for BMI: Bas F=24.03±4.55; Bas M=26.50±6.39; Adv F=21.80±2.75; Adv M=23.94±3.82 and the overall fitness score: Bas F=10.80±0.89; Bas M=11.07±0.85; Adv F=14.37±3.34, Adv M=11.84±3.56, and we get a clear picture of the physical and health condition of the subjects.

**Conclusions**

Using EFB, we identified students' poor physical condition in Arad. The fact that more than half of the students are in the basic group, is an alarming signal for us. A negative signal is also the fact that there were students who failed the test. But, receiving individual feedback and counselling immediately participants are able to act quickly to improve their physical and health condition.

## A33 Adverse childhood experiences and athlete mental health

### Uroš Perko^1^, Maša Černelič Bizjak^2^

#### ^1^New University, Ljubljana, Slovenia; ^2^Faculty of Health Science, University of Primorska, Izola, Slovenia

##### **Correspondence:** Uroš Perko (uros.perko@siol.net)

*BMC Proceedings 2024*, **18(11)**: A33

**Background**

Adverse childhood experiences, such as childhood trauma, have a negative impact on a range of health outcomes and can lead to long-term negative consequences, such as mental health problems and substance use in adulthood. However, as mental health problems in athletes have been increasingly reported in recent years, few studies based on a population sample of athletes have investigated whether early maltreatment may be the most important general historical factor associated with mental health problems in this population. The overall aim of the present study was to examine whether living in a dysfunctional family environment in childhood, measured as exposure to parental alcohol problems and trauma, is associated with other adversities in adulthood, reported and measured as psychological distress and harmful alcohol use.

**Materials and methods**

Fifty-seven elite athletes completed questionnaires. The mean age was 25 (SD = 10,04); 66% (*n* = 38) were female. Participants gave their consent by completing an online consent form and completing the survey, including the short version of the Screening Test for Children of Alcoholics (CAST-6), Childhood Trauma Questionnaire (CTQ), Alcohol Use Disorders Identification Test (only version AUDIT-C), and General Health Questionnaire (GHQ-12). This study was part of a larger study registered on Clinicaltrials.gov (NCT04259541).

**Results**

The sample reported moderate emotional neglect (44% of participants), emotional abuse (35% of participants), and physical neglect (25% of participants). 42% of athletes is adult children of alcoholics. Screening for risk of common mental disorders was found in 46% of athletes. 33% of athletes reported risky and harmful patterns of alcohol use.

**Conclusions**

Adverse experiences in early childhood have multiple, lifelong effects on stress responses and impaired psychological functioning that need to be considered in professional athletes and their functioning in order for them to receive support and treatment options.

## A34 Health improvement intervention at the workplace: Analysis of the perception of body technique as a physical activity within the work environment

### Ana-Marija Jagodić Rukavina, Karlo Šimanović

#### Kinesiology Academy of Body Technique, Zagreb, Croatia

##### **Correspondence:** Ana-Marija Jagodić Rukavina (anamarija@pbs.com.hr)

*BMC Proceedings 2024*, **18(11)**: A34

**Background**

Promoting physical activity at the workplace has become a key social determinant of health, given modern society's growing threat of inactivity and a sedentary lifestyle. In Croatia, 60% of the working population is inactive, and only 15% exercise regularly, confirming global trends (Barić, 2012). Despite this, there is a lack of education and training to create customized health programs within specific work environments.

**Materials and methods**

This research analyzes vitality (AMJR) and exercise behavior (BREQ-2) with validated questionnaires on 71 employees of the Kelteks factory, aged from 20 to 60 years, with an average of 38, divided into two groups – “office” and “manual” employees. The initial application of the questionnaires helped us identify differences in the perception of vitality and physical exercise among participants. It provided us a basis for assessing the current state before starting a three-month Body technique exercise intervention program for health improvement at the workplace. Monitoring of vitality and exercise behavior among the participants will be prominent for adjusting program methods, to preserve their health and work efficiency.

**Results**

Vitality questionnaire (AMJR) composed of seven categories indicated the effect of the three-month application of the Body technique. The questionnaire shows the level of vitality of the participants in both physical and mental domains. Final testing indicated improvements in five categories among employees, of which there was a significant improvement in the "posture" category (32%), and in the "breathing" category (44%), within “manual” employees.

**Conclusions**

This approach enables precise measurement of changes in attitudes and behaviors towards physical activity which could be crucial for tailoring future Body technique health programs and improving employee well-being at work. These results contribute to understanding the dynamics of physical activity and its connection with the work environment and will be presented and discussed in further research.

## A35 Gender differences in physical activity patterns among low-educational level people

### Vladimir Šipka^1^, Stefan Maričić^1^, Marko Kuridža^2^, Lidija Marković^1^, Damjan Jakšić^1^

#### ^1^Faculty of Sport and Physical Education, University of Novi Sad, Novi Sad, Serbia; ^2^Faculty of Sport and Psychology, Educons University, Sremska Kamenica, Serbia

##### **Correspondence:** Vladimir Šipka (vsipka14@gmail.com)

*BMC Proceedings 2024*, **18(11)**: A35

**Background**

Over the past few decades, Europe has witnessed notable growth in the development of policies and strategies promoting sports and physical activity. This survey focuses on the weekly engagement patterns in physical activities (frequency and level), encompassing sports, exercise, and everyday activities. Additionally, the survey explores the settings where individuals participate in physical activity, along with the motivators and barriers influencing sports involvement.

**Materials and methods**

The sample was consisted of 50 participants (25 men and 25 women) aged 26 and above with the lowest educational level, from Vršac. The Eurobarometer survey methodology was implemented in the study, addressing questions related to physical activity, settings, motivators, and barriers to engagement in physical activity.

**Results**

Statistically, there are no significant differences between men and women in the frequency of physical activities such as cycling, walking, dancing, or gardening. However, significant gender differences have been observed in some physically demanding activities, such as lifting weights, digging, aerobics, and fast cycling, favoring men (*p*=0.045). Additionally, men engage more frequently in vigorous physical activities compared to women (*p*=0.044). Regarding sedentary behavior, statistically significant gender differences have been noted (*p*=0.019), with men adopting a more sedentary lifestyle. Moreover, there are statistically significant differences in the choice of sports settings, such as sports centers and “at work”, favoring men (*p*=0.037 and *p*=0.002). There are no statistically significant gender differences in the reasons for engaging in or obstacles to physical activity, except among respondents who are already regularly involved in sports (*p*=0.047), also favoring men.

**Conclusions**

The conducted study reveals gender disparities in vigorous physical activities, sedentary behavior and certain choices of sports settings that favor men. Overall, the findings emphasize the necessity for nuanced approaches in promoting physical activity, particularly considering gender-specific preferences and behaviors within specific age and educational group.

## A36 Using Virtual Reality to test the anticipation skill of ice hockey players

### Anastasia Yakushina, Sergey Leonov

#### Department of Psychology, Lomonosov Moscow State University, Moscow, Russia

##### **Correspondence:** Anastasia Yakushina (anastasia.ya.au@yandex.ru)

*BMC Proceedings 2024*, **18(11)**: A36

**Background**

Nowadays virtual reality (VR) technologies have been applied in sports for testing and training certain skills. The purpose of this study was to develop and validate VR technology for diagnosing the skill of anticipation in hockey players.

**Materials and methods**

Twenty-seven athletes between the ages of 16 and 30 (Mage=18.3; SDage=2.9), 16 males and 11 females, participated in the study. The athletes needed to determine and fix in a virtual environment in which zone a flying puck would cross the goalie's zone.

**Results**

As a result, the most difficult to predict game patterns were identified. The most difficult for prediction were game situations in which the attacker made a shot at the puck from close distance from the side. The easiest were game situations in which the attacker made a hit from the center of the hockey arena. In addition, the results of mean comparison showed significant differences in the success rate of men and women (Mann-Whitney U-criterion=25.5; *p*=0.002, effect size=0.710). In this case, the average success rate of men in performing this task was 0.5944 (16 correct answers), while for women it was 0.3636 (10 correct answers). The obtained results may be related to the fact that, on the one hand, the level of training of men's and women's hockey teams may differ, and men are more likely to have experience of playing hockey in childhood and adolescence, unlike women. On the other hand, the task to be performed, in addition to hockey-specific skills, includes a skill related to reaction time, which can be developed, for example, in computer games.

**Conclusions**

Thus, the VR technology for diagnosing the anticipation skills in hockey players was tested, and the most difficult positions to anticipate, which require the most attention and training, were identified.

**Acknowledgment**

The work was supported by RSF grant No. 19-78-10134

## A37 Physical therapy following total knee arthroplasty: A case report

### Ana Kovač^1^, Ivan Perić^2^

#### ^1^Faculty of Dental Medicine and Health, “Josip Juraj Strossmayer” University of Osijek, Osijek, Croatia; ^2^Faculty of Kinesiology, “Josip Juraj Strossmayer” University of Osijek, Osijek, Croatia

##### **Correspondence:** van Perić (iperic@kifos.hr)

*BMC Proceedings 2024*, **18(11)**: A37

**Background**

Total Knee Arthroplasty represents a beneficial treatment for the advanced stages of arthritis and associated complications. The replacement of damaged bone parts with artificial implants contributes to the restoration of joint function, relieving patients from pain during the performance of basic life activities. This treatment is particularly recommended for individuals who have undergone unsuccessful non-operative, conservative interventions.

**Case report**

The patient involved in this case report is a 58-year-old male. Over the past 10 years, he has been complaining of pain in the left knee, and over time, the pain has progressed. Clinical and radiological findings confirmed advanced osteoarthritis of the left knee. The patient was admitted to the hospital on December 1, 2022, and stayed until December 8, 2022. During this period, a total knee arthroplasty was performed. Staples were removed 11 days post-operation, indicating successful wound healing. Rehabilitation started on January 17, 2023, completing it on February 6, 2023. The physiotherapy plans included hydrotherapy in a cooled pool, electromagnetic therapy on the knees, cryotherapy on the left knee, interferential currents on the right knee, electrostimulation on the quadriceps femoris muscle of the left knee, and therapeutic exercises. For the implementation of this case report, the ethics committee of Bizovačke Toplice granted its approval, and the patient himself signed an informed consent for their details to be published in an open-access journal.

**Conclusion**

During the three-week rehabilitation process, with the use of physical therapies, there have been visible improvements in the patient's physical condition and a positive subjective feeling reported by the patient. Therapeutic exercises have restored the patient's independence, increased confidence, balance, and stability. Thigh muscles have been strengthened, and the circumference of both thighs has increased by one centimeter compared to the beginning of the therapy. Swelling in the knee has decreased. Lower leg muscles in both legs have remained unchanged.

## A38 Conceptualization of sustainable development through civil and military sport

### Dubravko Marić^1^, Bojana Marić^2^

#### ^1^College of Sports and Health, Belgrade, Serbia; ^2^College of Vocational Studies for the Education of Preschool Teachers and Sports Trainers, Subotica, Serbia

##### **Correspondence:** Dubravko Marić (dubrasmaras@gmail.com)

*BMC Proceedings 2024*, **18(11)**: A38

**Background**

The growing interdisciplinary and multi-dimensionality of sustainable development and sports require significantly greater funds scientific and technical facts and information than before, which imposes the need for continuous scientific monitoring and study of various areas and phenomena. In the area of sustainable development and sport, there's less recognized boundary between military and civilian, national and international action and organization. The aim of the study is to establish an efficient conceptualization of the sustainable development through the civil and military sport.

**Materials and methods**

The study was carried out on the 202 respondents from 14 countries (military and civilians, active and former athletes and sports workers). They were surveyed using a questionnaire with 31 closed-ended questions. All of them were participants of the 25th Summer Universiade, the 55th World Military Cross Country Championship and the 13th Futsal Cup for Peace by International Military Sports Council (CISM).

**Results**

Eight factors were obtained with 64% of the variability that define the entire system of variables. The highest percentage of variability refers to benefits the army of Serbia membership in the CISM with about 28%. With 17% variability, the synergistic expression through the fourth, sixth, seventh and eighth factors determined sustainable development management. Civil-military cooperation determines the third and fifth factors with a common variability of about 11%. With 8% of the variability is isolated by the management of sports events factor.

**Conclusions**

A sport event is sustainable when it meets the needs of today's sport community and goes in the direction of future sport opportunities by improving the integrity of the natural and social environment on which it depends.

## A39 Effects of nine months developmental gymnastics exercise program on motor coordination in pre-school children

### Danilo Radanović^1^, Dragan Marinković^1^, Draženka Mačak^1^, Boris Popović^1^, Milan Cvetković^1^, Marko Gušić^1^, Slobodan Andrašić^2^, Milenko Janković^3^, Dejan M. Madić^1^

#### ^1^Faculty of Sport and Physical Education, University of Novi Sad, Novi Sad, Serbia; ^2^Faculty of Economics, University of Novi Sad, Subotica, Serbia; ^3^Preschool Teachers Training College, Novi Sad, Serbia

##### **Correspondence:** Danilo Radanović (danilo.radanovic@fsfvns.edu.rs)

*BMC Proceedings 2024*, **18(11)**: A39

**Background**

The established health advantages associated with physical activity (PA) and physical fitness (PF) are widely recognized. However, longitudinal investigations into the patterns of PA and PF during childhood have traditionally overlooked the contribution of motor coordination. This research aimed to examine the effects of developmental gymnastics exercise program on motor coordination in preschool children.

**Materials and methods**

The study cohort comprised 108 preschool children (74 boys; mean age 5.98 ± 0.48 years). Within this, the experimental group, engaging in a structured developmental gymnastics exercise program at a sports school, included 61 children (48 boys; mean age 5.96 ± 0.52 years), undergoing training twice weekly for one hour per session. The control group, drawn from the Preschool Institution "Radosno detinjstvo" in Novi Sad, Serbia, comprised 47 children (26 boys; mean age 6.02 ± 0.43 years). Only children in good health with parental consent were included in the study. The assessment focused on four items (walking backward, sideways jumping, shifting platforms, hopping on one leg) utilizing the Körperkoordinationstest für Kinder (KTK).

**Results**

The findings suggest a statistically significant and greater improvement (*p* < 0.05) in all four coordination tests (walking backward, hopping on one leg, sideways jumping, and shifting platforms) for the experimental group. The improvements were notably higher, with increases of 12.7%, 22.2%, 17.6%, and 10.5%, respectively, as compared to the control group.

**Conclusions**

Over 9 months, the implemented experimental intervention facilitated the attainment of elevated proficiency levels in all four specified skills among children in the gymnastics group. Such assessments contribute to an enhanced comprehension among researchers regarding the interplay between motor coordination and various variables, with a particular emphasis on its correlation with health-related outcomes.

## A40 Accuracy of CoM variability assessment using inertial motion sensors - a study case on martial arts

### Vlad Adrian Geantă^1^, Viorel Petru Ardelean^2^, Pierre Joseph de Hillerin^34^, Iosif Ilia^25^, Gyongy Osser^2^, Andrei Bitang^2^, Dana Rad^6^

#### ^1^Faculty of Physical Education and Sport, “Aurel Vlaicu” University of Arad, Arad, Romania; ^2^Faculty of Physical Education and Sport, Physical Activities Research Center, “Aurel Vlaicu” University of Arad, Arad, Romania; ^3^Doctoral School of Sport Science and Physical Education, National University of Science and Technology Politehnica Bucharest, Pitesti University Centre, Bucharest, Romania; ^4^Information for Sport and Human Performance Ltd., Bucharest, Romania; ^5^Doctoral school of “Victor Babes”, Faculty of Medicine, University of Medicine and Pharmacy, Timisoara, Romania; ^6^Centre of Research Development and Innovation in Psychology, Faculty of Psychology and Educational Sciences, Aurel Vlaicu University of Arad, Arad, Romania

##### **Correspondence:** Viorel Petru Ardelean (viorel.ardelean@uav.ro)

*BMC Proceedings 2024*, **18(11)**: A40

**Background**

Two athletes, participated in this study - S1: sex - F, age - 21 years, weight - 54 kg, height - 1.70 m, black belt, 12 years’ experience in karate, specialized in kata, and S2: sex - M, age - 18 years, weight - 60 kg, height - 1.80 m, brown belt, 6 years’ experience in karate, specialized in kumite. Athletes were tested using kinematic motion analysis equipment, MVN Biomech from Xsens. We analyzed: the oscillation and height variability of the CoM.

**Materials and methods**

The subjects performed 12 repetitions of Heian Nidan kata sequence. The results obtained were for S1: Mean Trajectory M.T.min = 0.795±0.005 m and M.T.max = 0.935±0.014 m; CVmax = 1.851%; and for S2: M.T.min = 0.790±0.002 m and M.T.max = 0.989±0.016 m; CVmax = 2.085%.

**Results**

Two athletes, participated in this study - S1: sex - F, age - 21 years, weight - 54 kg, height - 1.70 m, black belt, 12 years’ experience in karate, specialized in kata, and S2: sex - M, age - 18 years, weight - 60 kg, height - 1.80 m, brown belt, 6 years’ experience in karate, specialized in kumite. Athletes were tested using kinematic motion analysis equipment, MVN Biomech from Xsens. We analyzed: the oscillation and height variability of the CoM. The subjects performed 12 repetitions of Heian Nidan kata sequence. The results obtained were for S1: Mean Trajectory M.T.min = 0.795±0.005 m and M.T.max = 0.935±0.014 m; CVmax = 1.851%; and for S2: M.T.min = 0.790±0.002 m and M.T.max = 0.989±0.016 m; CVmax = 2.085%.

**Conclusions**

Analyzing the differences, we observe that the values recorded by S1 are smaller than S2, at CoM variation, which shows stability and precision in the execution of the technique. We can justify these positive results by the fact that S1 is older, is at a higher grade, but is also specialized in kata. With this technology we could highlight that a better control of the CoM, can be an indicator of correctness of execution especially in kata competitions.

## A41 Physiological profile of elite Serbian kumite athletes

### Sandra Vujkov, Bojana Marić

#### College of Vocational Studies for the Education of Preschool Teachers and Sports Trainers, Subotica, Serbia

##### **Correspondence:** Sandra Vujkov (vujkovsandra@gmail.com)

*BMC Proceedings 2024*, **18(11)**: A41

**Background**

Competitive Kumite is a short-duration fight that requires a high fitness level for athletes, especially motor and functional skills like speed, agility, muscle strength, flexibility, coordination, and balance. The purpose of this study was to determine the physiological profiles of senior male Kumite athletes of the Serbian national team.

**Materials and methods**

Nineteen senior male members of the Serbian Karate National Team (Age 21.68±3.02year; Body height 179.9±6.62cm; body weight 74.69±7.89kg) participated in this study. All participants were internationally ranked kumite athletes and participated in official WKF competitions, seven of whom were gold medalists in the senior category competing at the World Championship 2010. They all had at least eight years of training experience. Measures for body composition (BMI, Muscle mass, Fat mass), aerobic abilities (Forced Vital Capacity, VO2max absolute and relative), agility (T-test), maximal strength (1RM Bench Press and Back Squat), explosive power (concentric contraction - Squat Jump; eccentric-concentric contraction - Counter Movement Jump) flexibility (Sit and Reach) and endurance (Continuous Jump 15 sec) were obtained.

**Results**

Serbian elite Kumite athletes are characterized by relatively low body fat % (12.64±2.39), higher % of muscle mass (43.33±3.49), and normal BMI values (22.7±1.45kg/m2). Results of aerobic capacity: FVC (5.736±0.74), VO2max (3.43±0.64 l/min), VO2max (45.96±5.98 ml/kg/min), agility t-test (9.6558±0.26 sec) maximal strength 1RMBP (85.05 ±15.69 kg), 1RMBS (134.47±25.49 kg); explosive strength: SJ (45.737±3.98 cm), CMJ (47.65±6.04 cm); flexibility: Sit and Reach (10.39±7.22cm); and endurance CJ (42.85±4.11 cm) suggest that moderately high aerobic and lower-body explosive power, great muscular strength and good flexibility seem to be advantageous features for Kumite athletes.

**Conclusions**

The fitness profile of elite Serbian Kumite athletes could be used as a norm for talent identification. It could be of importance to sports scientists and coaches in optimal training planning and programming for monitoring training load and improving athletes’ performance.

## A42 Correlation between acute muscle damage and oxidative protection enzymes during different aerobic exercises

### Bojana Marić, Sandra Vujkov

#### College of Vocational Studies for the Education of Preschool Teachers and Sports Trainers, Subotica, Serbia

##### **Correspondence:** Bojana Marić (bokili2004@yahoo.com)

*BMC Proceedings 2024*, **18(11)**: A42

**Background**

Different types of aerobic exercise can cause different disorders of homeostasis. The objective of this study was to compare the effects of three different aerobic-type exercises (low-intensity continuous, high-intensity continuous, and high-intensity interval training) on muscle damage markers and antioxidative protection.

**Materials and methods**

Twelve female basketball players (age 17.7±4.3 years; weight 67.3±9.8kg; height 178.0±7.4cm) voluntarily participated in this study. A wash-out period of 7 days between single sessions of different training was provided to prevent the residual effect of interventions across study periods. Venous blood was drawn right before and immediately after each exercise session. Overall, 7 parameters of muscle damage markers were taken (creatine kinase (CK), creatine, creatinine, GAA, myoglobin (Mb), troponin, and lactate), while the oxidative protection was determined through 3 enzymes (SOD, CAT, GSH).

**Results**

As a marker of muscle damage, myoglobin (F=2.884; *p*=0.065) and lactates (F=5.254; *p*=0.008) make clear differences between training types. Creatinine, as a surrogate marker of creatine utilization and kidney function, shows statistical significance at the 0.05 level (F=4.053; *p*=0.022). The enzyme activity for oxidative protection shows statistically significant differences between groups for CAT (F=5.811; *p*=0.005) with different types of training intervention. The parameters of acute muscle damage that are high and the inclusion of antioxidant protection enzymes can change during the season due to different types of aerobic training and adaptation of the body to exercise-induced stress.

**Conclusions**

Training leads to the maintenance of physiological balance in the body and oxidative stress is not a necessary phenomenon of a high aerobic training load.

## A43 Evaluating the correlation between physical literacy and physical fitness in children aged 9-10 years

### Petra Rajković Vuletić^1,2^, Antea Šipalo Lilić^1^, Vladimir Pavlinović^1^

#### ^1^Faculty of Kinesiology, University of Split, Split, Croatia; ^2^Faculty of Kinesiology, University of Zagreb, Zagreb, Croatia

##### **Correspondence:** Petra Rajković Vuletić (petra.rajkovic@kifst.eu)

*BMC Proceedings 2024*, **18(11)**: A43

**Background**

Having good physical fitness (PF) is crucial for maintaining a healthy lifestyle. Physical literacy (PL) is described as the motivation, confidence, physical competence, knowledge and understanding to value and engage in a physically active health lifestyle. Previous research established a positive association between PL and PF, but little is known about it in early school-age children. The aim of this research was to determine the possible associations between PL and PF in children aged 9 and 10 years.

**Materials and methods**

The sample comprised children from Split, Croatia (*n* = 97, 48 girls, 9-10 years of age). Participants were tested on PF variables (standing long jump, curl up, trunk lift, push up 90°, sit and reach, and shuttle run 15m). The PL was evaluated by PLAYself questionnaire.

**Results**

Gender-stratified analyses showed weak correlations between PF and PL (Pearson's r: 0.03 – 0.23; *p* > 0.05). Biological maturity is known to be one of the most important determinants of PF in children, with those advanced in maturity having better performance on various fitness tests, it probably influenced even our result. Therefore, it is probable that the PF of our participants was strongly influenced by their biological maturity, which could reduce even the eventual influence of PL on PF.

**Conclusions**

In contrast to previous studies that confirmed a positive relationship between PL and PF, our results showed a weak relationship between PL and PF in children aged 9 and 10 years. Although not expected, results can be attributed to the influence of biological maturity on PF.

## A44 Integrating life skills in youth sports through critical pedagogy, virtue ethics, and ethics of care

### Milan Hosta

#### Faculty of Health Sciences, University of Primorska, Izola, Slovenia

##### **Correspondence:** Milan Hosta (milan.hosta@fvz.upr.si)

*BMC Proceedings 2024*, **18(11)**: A44

**Background**

In the realm of youth sports, a prevailing focus on physical skill and competitive triumph often overshadows the profound potential for holistic life skill development. This oversight has led to a significant underutilization of sports as a medium for comprehensive youth education. The prevailing notion that life skills in sports are 'caught, not taught,' stems from a lack of effective tools, awareness, and interest in integrating these skills systematically into sports training.

**Materials and methods**

Rooted in critical pedagogy, our approach calls for an educational framework in sports that is not only reflective and dialogical but also transformative. This framework challenges the traditional, coach-centric models, advocating for a participative environment where young athletes critically engage with their experiences, nurturing self-awareness and reflective thinking. Complementary to this is the application of virtue ethics, which underpins the development of character in sports, highlighting virtues such as integrity, respect, and fairness – crucial not only for sportsmanship but also for overall character development. The ethics of care adds a relational dimension, emphasizing empathy, caring for others, and building interpersonal relationships, vital for team sports and community involvement.

**Results**

Our findings indicate that Sportikus method significantly enhances self-awareness, ethical decision-making, empathetic understanding, and social responsibility among its users. Its user-friendly design transcends traditional teaching methods, making it not merely a tool for education but a catalyst for comprehensive development, ensuring that life skills are actively taught and caught.

**Conclusions**

In summary, the integration of critical pedagogy, virtue ethics, and ethics of care offers a groundbreaking approach to youth sports education. This approach redefines the role of sports as a multifaceted educational experience, preparing young athletes for diverse life challenges. Sportikus emerges as a crucial instrument in transforming the landscape of sports education, reemphasizing its role as a powerful agent for holistic youth development.

## A45 Physical activity as possible coping strategy for music performance anxiety

### Neda Aleksić^1^, Marina Đelić^2^, Jadranka Vlašić^1^

#### ^1^Faculty of Kinesiology, University of Zagreb, Zagreb, Croatia; ^2^Faculty of Medicine, University of Belgrade, Belgrade, Serbia

##### **Correspondence:** Neda Aleksić (neda.aleksic@student.kif.unizg.hr)

*BMC Proceedings 2024*, **18(11)**: A45

**Background**

Studies have shown that prevalence of anxiety, panic attacks and depression is significantly higher in musicians than in general population. One of promising strategies that is used in preventing and reducing general symptoms of anxiety is physical activity. Aim of this research was to examine level and relationship of physical activity (PA) and music performance anxiety (MPA) in music students who attend music high schools and music academies.

**Materials and methods**

The sample consisted of 81 participants (26 males and 55 females, 16 to 30 years old) who attend music high schools and music academies in Serbia, Republic Srpska and Bosnia and Herzegovina. Data were collected online, through Godin- Shephard leisure-time physical activity questionnaire and Kenny music performance anxiety inventory (K-MPAI). Descriptive and nonparametric statistic was used for processing of data.

**Results**

Overall score for PA and Health related score for PA were statistically significantly higher (*p*<0.001) in active students compared with moderate and insufficient active students. Obtained results for MPA indicate high overall level. According to cut-off of K-MPAI for clinical population, participants had a clinically significant level of MPA. Results showed that there is small but no significant difference in level of MPA according to level of PA at young musicians. Students who are active and moderately active showed lower level of music performance anxiety than students who are insufficiently active.

**Conclusions**

Our results didn’t show statistically significant difference in level of MPA according to level of PA. This could be due to small groups according to level of PA and larger sample could provide more reliable results. Also, more detailed and specific evaluation of PA is needed to determine which type and duration of PA affects level of MPA. Overall, our results indicate that PA should be examined more as a possible coping strategy for MPA.

## A46 The Sport Without Stereotypes (SWOST): An overview of the project development and outputs

### Bojan Mašanović^1,2^, Balša Kasćelan^3^

#### ^1^Faculty for Sport and Physical Education, University of Montenegro, Nikšić, Montenegro; ^2^Faculty of Law, University of Montenegro, Podgorica, Montenegro; ^3^Faculty of Law, University of Montenegro, Podgorica, Montenegro

##### **Correspondence:** Bojan Mašanović (bojanma@ucg.ac.me)

*BMC Proceedings 2024*, **18(11)**: A46

**Background**

Gender Equality is one of the leading indicators of Social Development. Despite the fact that the EU has made significant progress in gender equality over the past decades, inequalities still exist. The Sport without Stereotypes (SWOST) project aimed to promote equal access and participation for women and men at all levels and in all fields of sport and to encourage and increase the access and the participation of male and female in sport.

**Materials and methods**

It was focuses on the European priority encouraging social inclusion and equal opportunities in sport through addressing gender based sport with a specific focus on young people (10-18 years old). The SWOST project had a three-year duration (January/2021-December/2023), resulting in six main outputs: 1) online Assessment tool, 2) database and guidance towards existing best practices, 3) guideline of Quality Label, 4) memorandum of understanding of testing SWOST self-assessment tool, 5) collection of data about testing activities, and 6) the one scientific article, published on 1 international review.

**Results**

The creation of a SWOST online Assessment tool aimed to providing a qualitative self-assessment tool to discover how much the approach of sport associations is affected and deviated by gender-based stereotype. To support the implementation of the SWOST online Assessment tool, all other documents were created. Wide-spread and consistent adoption of the SWOST online Assessment tool, will provide self-assessment for clubs and sport associations to improve their policies and awareness.

**Conclusions**

This tool will enable sport association to measure progress in tackling the effects that gender stereotyping still has on athletes, young people and families, in relation with sport practice and clubs sporting choices.

**Acknowledgment**

This overview has been done within an ERASMUS + Sport project under the title SWOST - Sport Without Stereotypes that was approved by the EACEA (No. 622774-EPP-1-2020-1-ITSPO-SCP from 1 January 2021).

## A47 Numerical analysis of knee joint during jump among volleyball players

### Aleksandra Vulović^1,2^, Radivoje Radaković^2,3^, Nenad Filipović^1,2^

#### ^1^Faculty of Engineering, University of Kragujevac, Kragujevac, Serbia; ^2^Bioengineering Research and Development Center (BioIRC), Kragujevac, Serbia; ^3^Institute for Information Technologies, University of Kragujevac, Kragujevac, Serbia

##### **Correspondence:** Aleksandra Vulović (aleksandra.vulovic@kg.ac.rs)

*BMC Proceedings 2024*, **18(11)**: A47

**Background**

Volleyball is a dynamic sport that involves explosive movements and rapid changes in direction. These movements put a lot of biomechanical stress on the lower extremities, especially the knee joint. The main objective of this study was to analyze the knee joint using finite element methods and to investigate how the height and weight of volleyball players affect the stress distribution in the joint during jumping.

**Materials and methods**

This study involved three professional volleyball players with different physical traits. The numerical analysis required data on their height, weight, and jumping characteristics. A previously developed 3D model of the human knee joint was used to analyze the knee joint and obtain relevant information. The material properties were taken from the literature, while the loading conditions were derived from experimental measurements of the players’ jumps.

**Results**

The knee joint stress distribution during a jump depends on the interaction of different joint segments. The data of three volleyball players was used to calculate the stress distribution in their knee joints during jumping. The focus was on the meniscus, which plays an important role in load-bearing and shock absorption during jumping. The regions of menisci with the highest stress were identified, revealing potential injury sites.

**Conclusions**

Numerical methods, enable us to gain new insights and better understand the stress distribution in different segments of the knee joint. By using realistic models, we can obtain valuable information that can help us devise strategies to prevent injuries in volleyball players.

**Acknowledgement**

This research is supported by Ministry of Science, Technological Development and Innovation of the Republic of Serbia, contract numbers [451-03-47/2023-01/200107 (Faculty of Engineering, University of Kragujevac) and 451-03-47/2023 01/200378 (Institute for Information Technologies, University of Kragujevac)].

## A48 Development of basic and specific fitness skills in School of Football “Antunovac”

### Ivana Klaričić, Karlo Čorić

#### Faculty of Kinesiology, “Josip Juraj Strossmayer” University of Osijek, Osijek, Croatia

##### **Correspondence:** Ivana Klaričić (ivanak@kifos.hr)

*BMC Proceedings 2024*, **18(11)**: A48

**Background**

Previous research suggests that primary aim of the training process of the young school aged children is proper growth and development. The acquisition of technical and tactical skills is the secondary aim. The aim of this research was to determine the differences between the initial and final assessments of selected basic and specific fitness skills.

**Materials and methods**

Participants were 18 boys aged 8 and 9. They conducted a football training program in School of Football “Antunovac”, Croatia. The training program lasted for 12 weeks (3 times/week). Most of the program content were various games so the participants would embrace football and wouldn’t quit. The acquisition of technical and tactical skills was important but not the primary aim. Eight tests were used to assess fitness skills: sidestep (agility), sit and reach (flexibility), plank (static strength), 20m sprint (speed), dribbling 90° (specific agility), 6m shot (specific precision), juggling (specific coordination), 3 min. run (aerobic fitness). The paired t-test and the Wilcoxon test were used to determine the improvement.

**Results**

Statistically significant differences were determined in all conducted tests (*p* = 0.001 – 0.017). The highest improvement was determined in the 20m sprint and 3 min. run and the lowest in plank. The average program attendance was 75 %.

**Conclusions**

The highest improvement was determined in speed and aerobic fitness that are known to be in a sensitive stage of development at the age of 8 and 9. The lowest improvement was in static strength that is also known that is difficult to improve before the prepuberty. Although the main aim of the program wasn't the skill and abilities improvement, the significant improvement was achieved. Conclusion is that game based training program manages to accomplish both aims: the acquisition of technical and tactical skills and high rate of attendance.

## A49 Effects of resistance program exercise for the improvement of physical form, health biomarkers and quality of life of institutionalized older adults

### Jovan Vuković, Zoran Milošević, Marko Stojanović, Bojan Rašković

#### Faculty of Sport and Physical Education, University of Novi Sad, Novi Sad, Serbia

##### **Correspondence:** Jovan Vuković (jovan.vukovic@fsfvns.edu.rs)

*BMC Proceedings 2024*, **18(11)**: A49

**Background**

A growing number of scientific evidence suggests that resistance exercise has significant effects in reducing the deficits in strength, muscle mass, and functional capacity that occur as a result of aging. The subject of this research is the analysis of physical form, biomarkers of health, and quality of life of institutionalized older adults as well as whether there are effects of applying resistance exercise and what they are.

**Materials and methods**

The study included 22 older adults, who were divided into two groups: experimental (*n*=13) and control (*n*=9). The experimental group was subjected to resistance training for 12 weeks, while the control group had a normal lifestyle without programmed physical activity. All subjects were healthy and voluntarily participated in the research.

**Results**

The results of this study indicate resistance training leads to a significant increase in all parameters of physical form (which is contained in the battery of the Senior Fitness Test), all parameters of health biomarkers (lipid panel and blood glucose) and six of eight parameters of quality of life (which is contained in the SF-36 questionnaire).

**Conclusions**

After the performed analyses, in which the effects of resistance training on the improvement of physical form, health biomarkers and quality of life of institutionalized older adults were tested, it can be concluded that the effects of the training significantly influenced the improvement of the results in favor of the experimental group compared to the control group, which did not exercise.

## A50 Dribble Deficit in youth basketball players

### Mladen Mikić^1,2^, Boris Karasek^1^

#### ^1^Faculty of Sport and Physical Education, University of Novi Sad, Novi Sad, Serbia; ^2^Advanced Rehab & Conditioning Lab, Faculty of Sport and Physical Education, University of Novi Sad, Novi Sad, Serbia

##### **Correspondence:** Mladen Mikić (mladen.mikic@fsfvns.edu.rs)

*BMC Proceedings 2024*, **18(11)**: A50

**Background**

Dribble Deficit is a novel approach for evaluating dribble speed and ball control in basketball, as the time difference between sprinting with and without the ball. The aim of this study is to identify Dribble Deficit in youth male basketball players.

**Materials and methods**

The cross-sectional study was conducted on 27 male basketball players (9 players aged 14 years, 9 players aged 15 years, and 9 players aged 16 years). They completed two trials for each of the three linear sprint tests (20-m linear sprint, 20-m linear dribble sprint with stronger hand, and 20-m linear dribble sprint with weaker hand), and two change-of-direction sprint tests (Illinois agility test with and without ball) with 3-minute rest between every trial. The better results of the two trials to complete each test were recorded. The Dribble Deficit was calculated as a time difference between sprinting and dribbling speed for the corresponding test.

**Results**

Results of the ANOVA showed that there were significant differences (p≤0.05) between age categories for all five tests, with older groups exhibiting better results for every test except the Illinois agility test. T-test showed that the Dribble Deficit was significant for every corresponding dribbling vs. non-dribbling test in every age category, as well as for the entire sample regardless of participants' age (3.68±0.24 seconds for linear sprint test; 3.88±0.22 seconds for 20-m linear dribble sprint with a stronger hand; 4.03±0.23 seconds for 20-m linear dribble sprint with weaker hand; 17.52±0.97 seconds for Illinois agility test; and 18.64±1.13 for Illinois agility dribble test).

**Conclusions**

Dribble Deficit could be a valuable tool for evaluating dribble speed independently of sprinting speed in youth basketball players, and as a useful tool for assessing ball control during high-speed movement on the basketball court.

## A51 Differences in statistics between winning and losing U-17 men's and women's basketball teams

### Boris Karasek^1^, Igor Vučković^2^, Mladen Mikić^1,3^

#### ^1^Faculty of Sport and Physical Education, University of Novi Sad, Novi Sad, Serbia; ^2^Faculty of Physical Education and Sport, University of Banja Luka, Banja Luka, Bosnia and Herzegovina; ^3^Advanced Rehab & Conditioning Lab, Faculty of Sport and Physical Education, University of Novi Sad, Novi Sad, Serbia

##### **Correspondence:** Boris Karasek (karasek@uns.ac.rs)

*BMC Proceedings 2024*, **18(11)**: A51

**Background**

Quantitative analysis of game-related basketball statistics is a widely used tool by coaches and experts to develop team strategies, analyze opponents, and define areas for improvement. The aim of the present study was to identify game-related statistics that discriminate between winning and losing teams for both men and women under-17 (U-17) basketball teams of the Cadet League of Serbia.

**Materials and methods**

Games with a final score difference greater than 30 points were excluded from the analysis, so the final sample consists of 196 games (109 male games and 87 female games) from the 2022/2023 season of the Triglav Cadet League of Serbia. The following game-related statistics were gathered from the official box scores of the Basketball Federation of Serbia: 2- and 3-point field-goals (successful and unsuccessful), free throws (successful and unsuccessful), defensive and offensive rebounds, assists, steals, turnovers, blocks (committed and received), and fouls (committed and received). The sample was divided into three groups according to the final score difference: 76 close games (≤10 points), 72 balanced games (11 to 20 points), and 48 unbalanced games (≥21 points).

**Results**

Discriminant analysis results showed that in close games, both men's and women's winning teams differed from losing teams in successful 2-point field-goals (men, SC=0.407; women, SC=0.459), defensive rebounds (men, SC=0.449; women, SC=0.309), and assists (men, SC=0.563; women, SC=0.496), and also offensive rebounds in men's games (SC=0.312). In balanced games, both genders differed in assists (men, SC=0.366; women, SC=0.382) and women also differed in successful 2-point field-goals (SC=0.625). In unbalanced games, both genders differed in assists (men, SC=0.332; women, SC=0.306), men also differed in defensive rebounds (SC=0.342), and women differed in successful 2-point field-goals (SC=0.412).

**Conclusions**

These findings show that there are differences in game-related statistics for men's and women's U-17 basketball teams. This data could be valuable to coaches for developing training and game plans.

## A52 Acute effects of different “exercise snacking” interventions on glycemic control in patients with type 2 Diabetes Mellitus (T2DM): Study protocol for a randomized controlled trial

### Anja Lazić, Nebojša Trajković

#### Faculty of Sport and Physical Education, University of Niš, Niš, Serbia

##### **Correspondence:** Nebojša Trajković (nele_trajce@yahoo.com)

*BMC Proceedings 2024*, **18(11)**: A52

**Background**

Regular exercise has been shown to improve glycemic control, conventional exercise recommendations are not always feasible for patients due to time constraints. Additionally, the current guidelines are too general and provide unclear information on the intensity, duration, and frequency of activities. Thus, "exercise snacking” involving short bouts throughout the day, is a potential alternative. However, the real effects, intensity, volume, frequency and type for individuals with T2DM still need to be investigated. The aim of this study will be to examine the acute effects of “exercise snacking” modalities on glycemic control. Moreover, data on individual’s perceptions of each exercise intervention using measures of rate of perceived exertion (RPE), enjoyment, affect, blood pressure and adverse events will be collected.

**Materials and methods**

Ten sedentary patients diagnosed with T2DM without additional diseases will be recruited. The participants will receive all the following interventions in a random order: (1) three short sessions of 6×1 min of cycling at 90% of maximal heart rate (HR max) (2) three short sessions of 1 × 20 s of “all out” sprints at cycle ergometer and (3) no intervention. Glycemic parameters (the mean 24-h blood glucose level [mmol/L], time spent in hyperglycemia [% of the day spent above 10 mmol/L], incremental area under the curve [AUC] glycemic variability, and fasting blood glucose [FBG] will be monitored using continuous glucose monitoring system (iCGM) for each 24h period (baseline day, exercise day, and day following exercise). Additionally, Borg-10 scale, The Physical Activity Enjoyment Scale (PACES), Positive and Negative Affect Scale (PANAS), blood pressure and adverse events, will be conducted before and after each intervention.

**Results**

The results of this acute study have great potential to inform future public health efforts designed to improve glycemic control, increase exercise rates and affect overall health in individuals with T2DM**.**

## A53 The effect of a sports program on the development of motor abilities in preschool children

### Hrvoje Ajman, Zoran Špoljarić, Luka Sambol

#### Faculty of Kinesiology, “Josip Juraj Strossmayer” University of Osijek, Osijek, Croatia

##### **Correspondence:** Hrvoje Ajman (hajman@kifos.hr)

*BMC Proceedings 2024*, **18(11)**: A53

**Background**

Participation in various sports activities contributes to the improvement of intelligence, leadership skills, strength, endurance, and coordination in children (Demiral, 2011). The main goal of this study was to compare the motor abilities of kindergarten-age children who participate in multisport program in comparison with children who implement soccer training.

**Materials and methods**

The sample of respondents consisted of girls and boys from kindergarten Osijek (Kg group) (*N*=13), and boys members of the football clubs “FC Bedem Ivankovo” and “FC Frankopan Rokovci-Andrijaševci” (Fc group) (*N*=16), all are age group of four to six years. During the research, data on morphological characteristics (body height Mean= 109.71cm and body weight Mean= 24.17kg) were collected. Motor abilities were measured with tests: standing long jump, forward bend, shuttle run 5x10 meters and speed of four-legged backward walking. By analyzing the collected data, descriptive statistical indicators were calculated, arithmetic mean, dominant value, standard deviation, skewness, kurtosis, and minimum and maximum result value. A regular distribution of all variables was established. In further data analysis, the T-test for independent samples was used.

**Results**

Boys who play soccer achieve significantly better results in most motor abilities, especially those that measure speed-explosive properties. However, they performed lower flexibility compared to kindergarten children.

**Conclusions**

The children who play football performed better results in almost all motor abilities except flexibility. The reason can be found in the greater tonus of muscles of football players which negatively affects muscle flexibility. In further studies, it would be recommended to involve a greater number of participants in the study sample.

## A54 The intensity analysis during a male wheelchair tennis match-play between winners and losers of the Thai national team

### Khaothin Thawichai, Rachnavy Pornthep

#### School of Sports Science, Institute of Science, Suranaree University of Technology, Nakhon Ratchasima, Thailand

##### **Correspondence:** Rachnavy Pornthep (rachnavy@sut.ac.th)

*BMC Proceedings 2024*, **18(11)**: A54

**Background**

There was a lack of previous research investigating intensity analysis during a male wheelchair tennis match-play. The objective of this research study was to compare the intensity analysis during a male wheelchair tennis match-play between winners and losers of the Thai national team.

**Materials and methods**

Participants of this study were 8 male wheelchair tennis players of the Thai national team with an average age of 31.42±11.31 years. Before collecting data, the participants were tested for physical characteristics including weight, height, body fat percentage, and the Yo-Yo Intermittent Recovery Test (Level 1). During data collection, a round-robin competition was simulated. Best of 3 matches, the winner had to win 2 out of 3 games. Wireless heart rate monitor was installed to assess the heart rates during the competitions, record the results of the competition, and classify the winners and losers.

**Results**

Results of this study show that the maximum heart rates (HRmax) of the winner and loser groups during the competitions were no significantly different at 0.05 level, as well as heart rate variables in the intensity of winner and loser groups at 61-70 percent of HRmax.

**Conclusions**

This study suggested that the maximum heart rates of the winner and loser groups during the competitions were not significantly different. However, the maximum heart rates of loser groups at 71-80 and 81-90 percent of HRmax there is a higher rate than winner groups. This research can be used to improve training programs to be consistent and specific for male wheelchair tennis of the Thai national team in the future.

## A55 Rowing strategy of Thailand National Team in Asian Games 2023

### Rachnavy Pornthep, Khaothin Thawichai

#### School of Sports Science, Institute of Science, Suranaree University of Technology, Nakhon Ratchasima, Thailand

##### **Correspondence:** Khaothin Thawichai (thawichai.khaothin@gmail.com)

*BMC Proceedings 2024*, **18(11)**: A55

**Background**

Pacing strategy is the management of energy consumption during sporting events. This will affect athletic ability. Athletes used a variety of strategies during the competition, according to a study on rowing. The purpose of this research was to study the rowing strategies of the 19th Asian Games for the Thai national rowing team.

**Materials and methods**

The sample group consisted of 36 Thai national men's rowing athletes, with an average age of 24.69 years. The analysis of the racing strategy was performed using the official results of the ASIANGAMES 2023 regatta. Analyzed were the split times for each 500 m segment. The velocity of the boat was determined for each quarter of the event. According to Valery Kleshnev, the ratio of boat speed to "Gold Time" was calculated to facilitate comparisons across several boat types, including LM2X, M1X, M2X, M4X, M8+, W1X, LW2X, W2X, W4-, and W8+.

**Results**

According to the study, the boat's velocity varied during each quarter of each event. The following are the percentages of performance for each rowing category: W1X (105.73%, 100.53%, 97.74%, 96.49%), LM2X (103.10%, 99.28%, 97.85%, 99.92%), LW2X (104.97%, 99.75%, 97.83%, 97.79%), M2X (112.91%, 96.48%, 96.48%, 96.02%), W2X (104.10%, 99.46%, 98.60%, 98.06%), W4- (106.01%, 100.00%, 97.19%, 97.29%), M4X (106.26%, 100.49%, 98.89%, 95.01%), M8+ (104.33%, 102.02%, 98.28%, 95.81%), W8+ (106.28%, 101.45%, 96.51%, 96.42%). At the Asian Games 2023, the 2000m tactics (split 500m) for the Thai national rowing team have decreased by 12.25%.

**Conclusions**

The Thai national rowing strategy has been analyzed as guidelines for training and enhancing competitive strategies to maximize effectiveness.

## A56 Pre-post 3x3 basketball tournament changes in vertical jump force-time metrics

### Dimitrije Čabarkapa, Damjana V. Čabarkapa, Andrew C. Fry

#### Jayhawk Athletic Performance Laboratory – Wu Tsai Human Performance Alliance, University of Kansas, Lawrence, KS, USA

##### **Correspondence:** Dimitrije Čabarkapa (dcabarkapa@ku.edu)

*BMC Proceedings 2024*, **18(11)**: A56

**Background**

With being a relatively new research topic, the data pertaining to the physical performance characteristics of 3x3 basketball players is limited, especially at the elite level of competition. Thus, the purpose of the present study was to examine changes in neuromuscular performance characteristics pre-post a simulated 3x3 basketball tournament.

**Materials and methods**

Seven professional male basketball players (age= 19.2±1.1 years; weight= 84.6±9.5 kg; height= 193.3±7.2 cm) volunteered to participate in this study. Following completion of the warm-up protocol, before the start of the two-game tournament, each athlete completed three countermovement vertical jumps (CVJ) with hands on the hips while standing on a uni-axial force plate system sampling at 1000 Hz (Force Deck, Brisbane, Australia). Each jump trial was separated by a 15 sec rest interval and the mean value was used for performance analysis purposes. The identical testing procedures were conducted immediately following the completion of the second game. The force-time metrics examined in this study were mean eccentric (ECC) and concentric (CON) force and power and vertical jump height (VJH). Paired sample t-tests were used to examine pre-post changes in each dependent variable and Hedges g to depict the measure of the effect size.

**Results**

No statistically significant pre-post changes were observed in ECC mean force (834.3±94.6 vs. 829.4±92.4 N), CON mean force (1885.3±172.6 vs. 1944.6±161.4 N), ECC mean power (471.0±171.1 vs. 512.4±162.9 W), CON mean power (2992.9±285.3 vs. 3071.7±328.8), and VJH (44.4±4.0 vs. 43.5±6.3 cm). In addition, all effect sizes were small in magnitude (g=0.052-0.354).

**Conclusions**

The findings of the present study indicate that CVJ force-time metrics tend to remain relatively unchanged pre-post a simulated 3x3 basketball tournament. While providing sports practitioners with additional insight into sport-specific demands, these data also suggest that this cohort of athletes was adequately trained to sustain on-court competitive demands.

## A57 3D biomechanical analysis of the high jump technique of Greek female adolescent athletes

### Vassilios Panoutsakopoulos

#### Biomechanics Laboratory, School of Physical Education and Sport Science at Thessaloniki, Aristotle University of Thessaloniki, Thessaloniki, Greece

##### **Correspondence:** Vassilios Panoutsakopoulos (bpanouts@phed.auth.gr)

*BMC Proceedings 2024*, **18(11)**: A57

**Background**

The adolescent jumpers had significantly (*p*<.05) lower values in the take-off parameters (height, angle, horizontal and vertical velocity at take-off) and higher range of motion of the knee joint angle at the take-off leg, as well as more upright torso and less inclined placement of the take-off leg.

**Materials and methods**

Twelve Greek female adolescent athletes (15.8±1.1 yrs.) were recorded during competition with two cameras (sampling frequency: 60fps). A 3D-DLT analysis software (APAS v.14.1.0.5, Ariel Dynamics Inc., Trabuco Canyon, CA) was used for the kinematical analysis. Data from 7 elite females were retrieved from the Laboratory’s database. The Independent Samples T-test was used to detect significant (*p*<.05) differences between groups.

**Results**

The adolescent jumpers had significantly (*p*<.05) lower values in the take-off parameters (height, angle, horizontal and vertical velocity at take-off) and higher range of motion of the knee joint angle at the take-off leg, as well as more upright torso and less inclined placement of the take-off leg.

**Conclusions**

The results revealed that differences existed between the female adolescent and elite high jumpers in the parameters reflecting technique and physical conditioning. Thus, coaches should apply age-specific training programs to optimize high jump performance in female adolescent athletes.

**Acknowledgement**

The study is part of Research Project #74975 of the Research Committee of the Aristotle University of Thessaloniki (ethical approval no.: 260574/2022/06-10-2022), which is funded by the “Kostas Chimonides’ Athletes and Friends Club”. The funder had no role in the design of the study, the data analysis, and the decision to present the results.

## A58 Functional stability and mobility of middle-aged women with different occupations

### Miloslav Marković^1^, Mila Vukadinović Jurišić^2^, Anja Obradović^2^, Aleksandra Aleksić-Veljković^3^, Andrea Marković^3^, Jelena Obradović^2^

#### ^1^Academy of Applied Preschool Teaching and Health Studies, Kruševac; ^2^Faculty of Sport and Physical Education, University of Novi Sad, Novi Sad, Serbia; ^3^Faculty of Sport and Physical Education, University of Niš, Niš, Serbia

##### **Correspondence:** Mila Vukadinović Jurišić (mila.vukadinovic@fsfvns.edu.rs)

*BMC Proceedings 2024*, **18(11)**: A58

**Background**

The aim of this study was to determine the differences in functional stability and mobility of middle-aged women with different occupations.

**Materials and methods**

The participants comprised 30 middle-aged women divided into two groups according to occupation. The first group is the sewing workers (*N*=15; age: 51.67±6.32 years; body height 168.80±3.87 cm; body mass 70.87±8.63 kg), and the other group was kindergarten teachers (*N*=15; age 46.60±5.97 years; body height 168.80±3.88 cm; body mass 70.87±8.63 kg). The functional movement screen (FMS) was used to evaluate their functional stability and mobility. Kruskal-Wallis comparison tests were used to compare individual FMS scores between groups. Additionally, univariate analysis of variance (ANOVA) was used to determine the differences in FMS Total Score across groups.

**Results**

The results show differences (p≤0.05) between the groups in the tests: Deep Squat, Hurdle Step, In-Line Lunge, Shoulder Mobility, and Trunk Stability Push-Up in favor of the kindergarten teacher. Meanwhile, no statistically significant differences (p≥0.05) were observed between kindergarten teachers and sewing workers in Active Strength Leg Raise and Rotary Stability. In FMS Total Score achieved statistically significant difference (p≤0.05) between groups (p≤0.05). The kindergarten teachers achieved higher results (17.33±2.22) than sewing workers (13.27±3.06).

**Conclusions**

The research findings of this study indicate that kindergarten teachers had good functional mobility of the shoulder, hip, knees, and ankles joints. In addition, they also had excellent functional stability of the trunk. The sewing workers had impaired functional mobility of the shoulders, hips, knees, and ankles joints. Additionally, sewing workers had weak functional stability of the trunk. Based on that, sewing workers should engage in exercises that would improve the stability of the trunk but also the mobility of the shoulder, hips, knees, and ankles joints. Middle-aged women with sedentary occupations should engage in physical activity to avoid functional immobility and improve work productivity.

## A59 Descriptive biomechanical parameters of winning performance in men's artistic gymnastics high bar discipline: A national championship: Case study

### Sara Aščić

#### Faculty of Kinesiology, University of Zagreb, Zagreb, Croatia

##### **Correspondence:** Sara Aščić (ascic.sara@gmail.com)

*BMC Proceedings 2024*, **18(11)**: A59

**Background**

The High bar, a men's discipline in artistic gymnastics, involves athletes executing aerial stunts on a horizontal bar. The enhancement of techniques and, consequently, performance outcomes rely heavily on comprehensive biomechanical and notation data. The analysis of kinematics in elite athletes is particularly valuable for trainers, enabling them to compare and assess the performance of their athletes against benchmarks set by top performers. The objective of this case study is to ascertain the descriptive parameters of the winning performance in the high bar discipline during its execution at the national championship.

**Materials and methods**

The performance of the national championship winner in the horizontal bar discipline was examined in this study. GoPro 360 max was used to collect video materials of performance. The recording resolution was set at 3840 x 2160 pixels with 50 frames per second and the camera is positioned laterally relative to the horizontal bar, with the focal point aligned along the extension of the bar. Kinovea software was utilized to obtain 2D biomechanical parameters of feet during performance on high bar and for notation analysis.

**Results**

The average speed of the athlete's feet during the performance registered at 21.25 km/h (Min: 11.00 - Max: 55.00). The overall performance duration was 43.76 seconds, with the athlete spending 40.33% of the time below the bar, 56.83% above the bar, and 2.83% dedicated to the landing phase. The athlete-maintained contact with the bar for 91.93% of the routine, spending 8.07% of the time airborne.

**Conclusions**

This study delineates critical biomechanical metrics, illuminating the nuanced performance dynamics of elite-level high bar routines in men's artistic gymnastics.

## A60 Differences in motives for exercise between participants of different group exercise programs

### Ana Kraml, Danijela Kuna, Klara Findrik

#### Faculty of Kinesiology, “Josip Juraj Strossmayer” University of Osijek, Osijek, Croatia

##### **Correspondence:** Danijela Kuna (danijela.kuna@gmail.com)

*BMC Proceedings 2024*, **18(11)**: A60

**Background**

The subject of numerous studies is examining the motives that make people engage in various exercise programs. This research was conducted with the aim of determining the differences in the motives of exercisers under the influence of different group exercise programs.

**Materials and methods**

The research included a total of 40 female subjects between the ages of 27 and 57 who participated in different models of group exercise programs three times a week for a period of 12 weeks. The first group consisted of 22 test subjects, and the second group consisted of 18 subjects. After the end of the treatment, an online questionnaire was conducted among the respondents. The respondents chose how moderately, very, and extremely important the motives for participating in group exercise programs are to them. The statistical significance of differences between groups was examined using the Chi square test and Fisher's exact test.

**Results**

Statistically significant differences between the groups in the observed exercise motives were found when examining the importance of improving and preserving health (*p*<0.001) and increasing muscle mass (*p*<0.05). Reducing stress proved to be an equally important motive for coming to workouts in both groups, while the importance of reducing subcutaneous fat tissue was much greater for the first group's exercisers by 16.7%. A good appearance turned out to be the least important reason why female exercisers work out, especially among female exercisers in the first group (59%). In general, the primary goals of the first group were to improve and preserve health and reduce subcutaneous fat, while the primary goals of the second group were to improve and preserve health and increase muscle mass.

**Conclusions**

It can be concluded that female exercisers have a developed awareness of the numerous positive effects of physical exercise on improving health and on weight control, thus improving overall psychophysical health.

## A61 Comparative Analysis of morphological characteristics between female and male second-year students at the Faculty of Kinesiology Osijek

### Iva Macan^1,2^, Josip Cvenić^1^, Marin Marinović^1,2^

#### ^1^Faculty of Kinesiology, “Josip Juraj Strossmayer” University of Osijek, Osijek, Croatia; ^2^Faculty of Kinesiology, University of Zagreb, Zagreb, Croatia

##### **Correspondence:** Iva Macan (imacan@kifos.hr)

*BMC Proceedings 2024*, **18(11)**: A61

**Background**

In the contemporary kinesiological context, the significance of studying the morphological characteristics of students is crucial for adapting and optimizing physical activity programs. This study is focused on analyzing differences in morphological parameters between second-year students at the Faculty of Kinesiology Osijek, aiming to gain a deeper insight into variations in body composition within this population.

**Materials and methods**

The sample consisted of 47 students (35 males and 12 females). The students are enrolled in the Faculty of Kinesiology Osijek in their second year of study. The anthropometric variables utilized in this research include body weight (TM), height (TV), measurement of 7 skinfolds, and the calculation of Body Mass Index (BMI). The normality of the distribution was tested using the K-S test, which showed that the data is normally distributed. In further analysis, an independent samples t-test was used.

**Results**

The results indicate a statistically significant difference in BMI between male and female students (*p*=0.00). The average height for second-year female students is 167.88 cm, with a body weight of 61.74 kg, while male students have an average height of 182.75 cm and a body weight of 81.29 kg. Although differences exist in most anthropometric variables, the statistically most significant disparities in skinfold measurements are observed in variables SUPKN (*p*=0.00) and KNN (*p*=0.00).

**Conclusions**

Disparities in skinfold measurements at the supra iliac and thigh regions between genders can be attributed to biological differences in the distribution of adipose tissue, hormonal effects, and genetic factors. Consequently, observed statistical variances in these anthropometric measures underscore biological distinctions among male and female students. Furthermore, the diverse sporting backgrounds of the students are likely to exert an impact on their morphological structures.

## A62 Isokinetic and isotonic quadriceps exercise after knee surgery: A retrospective study

### Siniša Nikolić^1,2^, Borislav Obradović^3^, Vanja Dimitrijević^3^, Bojan Rašković^3^, Dragana Dragičević - Cvjetković^1,4^

#### **Correspondence:** Bojan Rašković

*BMC Proceedings 2024*, **18(11)**: A62

https://www.frontiersin.org/articles/10.3389/fresc.2024.1336847/full

## A63 Factors for choosing glamping as a way to spend a physically active holiday

### Irena Kleibencetl^1^, Miha Lesjak^2^, Klemen Širok^1^, Matej Plevnik^1^

#### ^1^Faculty of Health Sciences, University of Primorska, Izola, Slovenia; ^2^Faculty of Tourism Studies – Turistica, University of Primorska, Portorož, Slovenia

##### **Correspondence:** Irena Kleibencetl (irena.kleibencetl@fvz.upr.si)

*BMC Proceedings 2024*, **18(11)**: A63

**Background**

Glamping, short for glamorous camping, is a form of outdoor accommodation that combines the experience of camping with luxurious amenities and comfort. The aim of this research is to provide camping managers with valuable insights so that they can adapt to the changing needs of their guests and align their strategic management accordingly.

**Materials and methods**

Data was collected through an online survey via all social networks of University of Primorska. The questionnaires were completed by 413 people aged 33.8 ± 12.92 years of both genders (130 males and 283 females). The data was collected as part of the project “Placement of urban glamping in innovative forms of tourist products of the Istrian hinterland”.

**Results**

The results show that glamping, is becoming a popular trend and represents a new opportunity for campsite operators to attract new and younger guests who previously preferred to stay in hotels and apartments. Glamping is also a type of accommodation that promotes a physically active form of holiday. Guests choose glamping because they are looking for peace and relaxation (4,43), to get in touch with nature (4,36) and to have a different experience at least once in their lives (4,23). Despite this, they are not prepared to give up water (4,45), their own bathroom (4,10) and electricity (3,93). At the same time, they expect a wide range of services at the glamping site itself and a rich and varied offer at the destination itself.

**Conclusions**

The results of the study provide some interesting clues for managers of this type of tourism business, both for product development and for increasing communication efficiency. Guests opt for glamping because they like the idea of hidden places and glamour, but many do not opt for this experience because of the high price and for once, the limited offer.

## A64 Anthropometric characteristics of students at the Faculty of Kinesiology

### Klara Findrik^1,2^, Iva Macan^1,2^, Danijela Kuna^1^

#### ^1^Faculty of Kinesiology, “Josip Juraj Strossmayer” University of Osijek, Osijek, Croatia; ^2^Faculty of Kinesiology, University of Zagreb, Zagreb, Croatia

##### **Correspondence:** Klara Findrik (kfindrik@kifos.hr)

*BMC Proceedings 2024*, **18(11)**: A64

**Background**

Studying the anthropometric characteristics of students at the Faculty of Kinesiology increases understanding and potential variations in the physical condition among students at the Faculty of Kinesiology, which can contribute to a broader understanding of physical performance in an academic environment.

**Materials and methods**

The sample included 58 students from the second and third years of the Undergraduate University Study Program of Kinesiology at the Faculty of Kinesiology Osijek, consisting of 30 students from the part-time undergraduate program and 28 students from the regular undergraduate program. The variables measured were body height (TV) and body weight (TM), from which the body mass index (ITM) was calculated.

**Results**

The normality of the distribution was tested using the K-S test, which showed that the data is normally distributed. In further analysis, an independent samples t-test was used. A statistically significant difference was found in the variables of body weight (*p*=0.00) and body mass index (*p*=0.00). Students from the part-time study program had an average of 79.75 kg of body weight, while students from the regular study program had an average of 70.20 kg. Additionally, the body mass index for students in the part-time study program was 36.01, compared to 28.35 for students in the regular study program.

**Conclusions**

The difference in body mass index between regular students at the Faculty of Kinesiology and those who study part-time may stem from a combination of stressful work environments, limited time for physical activity, accessibility to resources, and disparities in work and dietary habits. These factors, along with potential genetic predispositions, can contribute to changes in body weight and body mass index, underscoring the need for further research to better understand the specific causes of this variation.

## A65 Physical fitness status in middle-aged women: A preliminary examination of the long-term effect of the participation in aquatic fitness programs

### Mariana C. Kotzamanidou^1,2^, Alexandra Stampouli-Drossopoulou^1^, Vassilios Panoutsakopoulos^3^, Panagiotis Siaperas^2,4^, George A. Tsalis^5^, Victoria Misailidou^1^

#### ^1^Faculty of Health Sciences, Metropolitan College of Thessaloniki, Thessaloniki, Greece; ^2^Institute of Occupational Science & Rehabilitation, Metropolitan College, Athens, Greece; ^3^Biomechanics Laboratory, School of Physical Education and Sport Science at Thessaloniki, Aristotle University of Thessaloniki, Thessaloniki, Greece; ^4^Occupational Therapy Department, Metropolitan College, Athens, Greece; ^5^School of Physical Education and Sport Science at Serres, Aristotle University of Thessaloniki, Thessaloniki, Greece

##### **Correspondence:** Mariana C. Kotzamanidou (mkotzamanidou@mitropolitiko.edu.gr)

*BMC Proceedings 2024*, **18(11)**: A65

**Background**

Women's health is of importance to cope with life span demands. During the lifespan, women experience hormonal, vasomotor, and psychological mood alterations, along with cognitive and body composition changes. Mental and physical resilience is the balance key to wellbeing. Water-based fitness programs are considered an effective alternative to therapeutic exercise regarding the physical ability of middle-aged women. The purpose of the study was to determine the differences in physical fitness parameters in middle-aged women with long-term participation in aquatic fitness programs compared to sedentary age-matched individuals.

**Materials and methods**

Middle-aged women were recruited and regularly evaluated from a local community in the suburbs of Thessaloniki, Greece. Participants formed the long-term aquatic fitness group (*n*=12, 58.8±6.3yrs, 163.5± 5.4cm, 71.6±13.9kg) with systematic participation (two sessions/week for 7.8±4.0yrs) in aerobic water exercise programs, and the sedentary group (*n*=7, 58.7±4.9yrs, 162.6± 5.7cm, 71.5±11.4kg) comprised of women who reported no systematic activity. All the participants experienced musculoskeletal pain in the neck, lumbar, and hip. The evaluation of physical fitness included balance tests, muscular strength tests, tests to assess trunk mobility and the flexibility of the lower extremities, and tests to assess aerobic capacity. Following the results of the Shapiro-Wilk test for normality, the Independent Samples T-test and the Mann-Whitney U Test were used to detect significant (*p*<.05) differences between groups.

**Results**

The aquatic fitness group scored significantly (*p*<.05) better in the Romberg-Tandem balance test with closed eyes. Significantly (*p*<.05) lower inter-limb asymmetry in the 5-s isometric hand-grip strength test was observed in the sedentary group. No differences were revealed for the heart rate and oxygen saturation in the 6-min walking test, the mobility/flexibility tests, and the strength tests.

**Conclusions**

Results revealed that the middle-aged women involved in long-term aquatic fitness programs exhibited better proprioception compared to sedentary peers. As balance is essential for fall prevention, aquatic exercise is recommended.

## A66 Plantar pressure and force asymmetry in elite gymnast

### Dragan Marinković, Danilo Radanović, Aleksandra Ilić, Aleksandra Rajčić, Dejan M. Madić

#### Faculty of Sport and Physical Education, University of Novi Sad, Novi Sad, Serbia

##### **Correspondence:** Dragan Marinković, dragan.marinkovic@fsfvns.edu.rs

*BMC Proceedings 2024*, **18(11)**: A66

**Background**

Plantar pressure and force analysis play a important role in understanding foot biomechanics and assessing the risk of lower extremity complications and conditions. In gymnastics, plantar parameters are influenced by various factors such as foot anatomy, motor abilities, and the characteristics of the competition discipline. This study aimed to determine asymmetry in plantar pressure and force characteristics between the left and right legs in elite gymnasts.

**Materials and methods**

Eleven male and female elite gymnasts (age: 20.08±5.57 years; height: 160.5±14.58 cm; weight: 56.8±18.04 kg) participated in this cross-sectional study. Plantar pressure and force measurements were conducted during a three-step gait analysis using the Foot Work Pro system's footplate and Auto Mask function in ten plantar regions for both legs in a barefoot condition.

**Results**

The Paired Samples T-test showed no asymmetry in step duration, plantar peak pressure, contact area, center of force speed peak, center of force mean speed, center of force acceleration, or center of force distance. Nevertheless, asymmetry was identified in the pressure (kPa) of the Hallux plantar region (*t*=-2.7; *p*=0.02) and the Internal Heel region (*t*=2.69; *p*=0.02), whereas no significant asymmetry was observed in the remaining eight-foot regions.

**Conclusions**

The observed asymmetry in plantar pressure and force characteristics, particularly in the Hallux and Internal Heel regions among elite gymnasts, underscores the importance of considering these factors in training and injury prevention strategies. While no significant asymmetry was found in overall gait plantar parameters, the localized differences in pressure and force distribution suggest specific biomechanical challenges that gymnasts may face. Continuous monitoring of plantar pressure and force characteristics can contribute to the development of personalized training plans, enhancing the overall biomechanical efficiency of gymnasts and promoting their musculoskeletal abilities.

## A67 Aerobic and cognitive training during hemodialysis: A randomized controlled trial

### Špela Bogataj^1,2^, Maja Pajek^2^, Aljaž Kren^2^, Katja Kurnik Mesarič^1^, Jernej Pajek^1^

#### ^1^Department of Nephrology, University Medical Centre Ljubljana, Ljubljana, Slovenia; ^2^Faculty of Sport, University of Ljubljana, Ljubljana, Slovenia

##### **Correspondence:** Špela Bogataj (spela.bogataj@kclj.si)

*BMC Proceedings 2024*, **18(11)**: A67

https://www.sciencedirect.com/science/article/pii/S2468024924016528?via%3Dihub

## A68 Anthropological characteristics of Osijek basketball referees

### Mihovil Psihistal, Zvonimir Tomac, Tvrtko Galić

#### Faculty of Kinesiology, “Josip Juraj Strossmayer” University of Osijek, Osijek, Croatia

##### **Correspondence:** Zvonimir Tomac (ztomac@kifos.hr)

*BMC Proceedings 2024*, **18(11)**: A68

**Background**

Basketball referees, along with the players, are an integral part of the basketball game, and their role in the outcome of the game is very significant, sometimes even decisive. Testing of them consists of a weigh-in where judges under the age of 30 must have a body weight less than the value measured in the calculation (height in centimeters minus 105). For judges over 30 years old, the principle is the same, only 100 is subtracted from the height.

**Materials and methods**

This research aimed to determine the anthropological profile of Osijek basketball referees (*N*=12). Their morphological characteristics and functional abilities are measured. Data on body composition and morphological characteristics were collected with an Omron BF 511 digital scale, and body height was measured using a SECA stadiometer. Functional ability was assessed by the BEEP test

**Results**

The results showed that the referees have an adequate anthropological profile. Referees have a BMI of 25.39, a percentage of fat of 20.34% and a percentage of muscle of 38.74%. The BEEP test result showed that referees have VO2max of 49.58 (ml/kg/min). It can be concluded that Osijek basketball referees have an above-average anthropological profile compared to the general population. They also have a high level of aerobic capacity in which they do not lag behind even the most elite referees in the world.

**Conclusions**

In the category of elite Croatian judges, Osijek judges of all categories had a higher percentage of fat. Despite the higher percentage of fat, they do not lag behind in aerobic capacity, which may be a consequence of the fact that there are many young referees in Osijek with a high aerobic capacity and a higher percentage of fat.

## A69 Exploring differences in postural parameters between elderly individuals engaged in specialized training for one year or more and those without specialized program participation

### Marin Marinović^12^, Sara Aščić^2^, Iva Macan^1,2^

#### ^1^Faculty of Kinesiology, “Josip Juraj Strossmayer” University of Osijek, Osijek, Croatia; ^2^Faculty of Kinesiology, University of Zagreb, Zagreb, Croatia

##### **Correspondence:** Sara Aščić (ascic.sara@gmail.com)

*BMC Proceedings 2024*, **18(11)**: A69

**Background**

Postural imbalance affects body stability, leading to a shifted center of mass beyond support limits, increasing the risk of falls and contributing to the development of muscular imbalances and asymmetries, particularly detrimental to elderly individuals. This study aims to determine differences in selected posture parameters between active elderly individuals engaged in specialized programs for over one year and those who have recently initiated training in selected posture parameters.

**Materials and methods**

Twenty-two elderly individuals (mean age 67.10±5.23 years; height 164.90±10.41 cm; weight 74.43±15.59 kg) were included in this study. Twelve participants were engaged in specialized programs for the elderly for more than one year, while ten individuals had recently joined these programs. The PhysioMaster mobile application, a widely used tool for posture assessment, was employed to compare postural parameters between two groups. This study analyzed various variables, including head tilt, shoulder alignment, pelvic tilt, patella position, knee Q angle, tibia position, foot rotation, vertical alignment, sagittal head tilt, craniovertebral position, head-shoulder position, pelvic tilt, hip alignment, knee alignment, knee flexion, scapular alignment, and foot pronation.

**Results**

Our findings indicate that individuals participating for a longer duration in specialized programs exhibited a greater outward rotation of the right leg (*p*=0.04), increased backward vertical alignment (*p*=0.03), and lower anterior pelvic tilt (*p*=0.04) compared to participants who recently joined these programs.

**Conclusions**

Active elderly individuals engaged in specialized programs exhibit distinct postural parameters compared to those who do not participate, with particular differences observed in outward rotation of the right leg, backward vertical alignment, and anterior pelvic tilt.

## A70 Efficiency of physical education classes among the students: A longitudinal study

### Milan Cvetković^1^, Vladimir Miljković^1^, Nikola Manolopoulos^1^, Miloš Kojić^1^, Slobodan Andrašić^2^

#### ^1^Faculty of Sport and Physical Education, University of Novi Sad, Novi Sad, Serbia; ^2^Faculty of Economics, University of Novi Sad, Subotica, Serbia

##### **Correspondence:** Milan Cvetković (milan.cvetkovic@fsfvns.edu.rs)

*BMC Proceedings 2024*, **18(11)**: A70

**Background**

Motor skills play a crucial role in an individual's daily functioning and overall physical well-being, and physical activity engagement significantly contributes to the development of different motor abilities. This longitudinal study aimed to examine the effects of physical education classes on students' motor skills over a period of 23 months.

**Materials and methods**

The study involved 18 participants aged between 20 and 23 years from the University of Novi Sad. The experimental design included two physical education classes per week over four academic semesters (23 months). Motor abilities were evaluated using standardized tests, including the standing long jump, sit-and-reach test, 60-second sit-up test, push-ups, and hand grip strength test on initial and final testing, with two control checks.

**Results**

Repeated measures ANOVA indicated significant differences, particularly in the number of push-ups (F = 10.28; *p* = 0.00) during the experimental period. Bonferroni post-hoc tests revealed that students performed significantly more push-ups from initial to final testing. In the Standing long jump (F = 3.20; *p* = 0.06), Sit-and-reach test (F = 1.80; *p* = 0.20), 60-second sit-up test (F = 2.96; *p* = 0.07), and Hand grip strength test (F = 1.80; *p* = 0.20), there were some increasement but no significant effects of physical education classes.

**Conclusions**

This longitudinal research suggests that practical classes in physical education positively influence the development of some motor skills in university students. Physical education classes could be beneficial for general physical components related to well-being and health. However, future research should explore the potential benefits of increased frequency in physical education classes at the university level.

## A71 Triple lumbar disc herniation – improving the quality of life through physical exercising: longitudinal case study

### Kristina Malečkar^1^, Marko Kapeleti^1^, Vuk Stevanović^2^, Vladimir Mrdaković^1^, Marija Macura^1^

#### ^1^Faculty of Sport and Physical Education, University of Belgrade, Belgrade, Serbia; ^2^Institute for Medical Research, University of Belgrade, Belgrade, Serbia

##### **Correspondence:** Kristina Malečkar (kristinamaleckar@yahoo.com)

*BMC Proceedings 2024*, **18(11)**: A71

**Background**

Lumbar disc herniation is an increasingly common phenomenon, significantly impacting work productivity, mental health, and quality of life. It alters functional movement patterns, and conditions incorrect compensatory body positioning, causing muscle dysfunction. Physical exercising, particularly strengthening deep back, rectus and oblique abdominal, and thigh muscles, is a main treatment for chronic cases, aiding spinal stabilization and pain reduction.

**Materials and methods**

A case of a 37-year-old male diagnosed with triple lumbar disc herniation of moderate severity and degenerative disc changes at the spinal levels of L3-L4, L4-L5, and L5-S1, identified by magnetic resonance imaging, was examined. The subject conducted a 10-week exercise program, 4 to 5 times a week, 45 minutes each. The program was conducted at home, included variations of body-weight exercises with additional equipment, and gradually increased in intensity and volume. The exercises focused on increasing hip joint mobility and strengthening deep back, rectus and oblique abdominal, and thigh muscles. Initial and final testing was conducted to compose an individualized exercise program and evaluate its effects. The testing consisted of a pain questionnaire, a 36-item short-form survey on quality of life, Manual Muscle Testing (MMT), Functional Movement Screen (FMS), and body composition analysis – InBody770.

**Results**

Questionnaires qualitatively showed significant pain reduction and improved physical and psycho-social condition. Tests showed significant improvements in MMT (before – 4, after – 5), FMS (before – 2, after – 3), and body composition: weight (+1.5%), body fat (-6.6%), body water (+3.4%), skeletal muscle (+2.8%).

**Conclusions**

Subject reduced pain, improved physical and psycho-social condition, muscle strength, movement patterns, and body composition. Case study confirms exercise program efficacy for treating lumbar disc herniation and enhancing quality of life. Tests are practical, accessible. Program suitable for preventing lumbar disc herniation and improving general population's quality of life.

The patients provided informed consent for their details to be published in an open-access journal.

## A72 Exploring the relationship between on-ice testing, spatial attention, and game performance in elite junior ice hockey players

### Margarita M. Tcepelevich, Viktor V. Bolshakov

#### Sirius University of Science and Technology, Sirius, Krasnodar region, Russia

##### **Correspondence:** Margarita M. Tcepelevich (riks00022@gmail.com)

*BMC Proceedings 2024*, **18(11)**: A72

**Background**

Research consistently shows that both cognitive and physical abilities are crucial in ice hockey. However, the association between an athlete's skills and on-field performance in competitive junior hockey is poorly understood. The purpose of this study is to evaluate the effects of skating skills and spatial attention on match performance in elite junior hockey.

**Materials and methods**

A total of 195 participants of the Russian National Hockey Championship U15 (male defensemen, age: 14.76 ± 0.34 years) completed on-ice Forward and Backward sprint tests, Transition agility test, Weave agility test and performed Multiple Object Tracking (MOT) task to assess attention. Match performance metrics (Goals against, Giveaways, passing accuracy) were collected using Iceberg Sports Analytics tool throughout all matches of the Championship. The relationships between match performance and test scores were modeled using multiple linear regression, taking into account team ranking. Informed consent was obtained from participants’ parents before the experiment. The study was approved by the Sirius University Ethics Board.

**Results**

The study found significant associations between Giveaways and both the accuracy of the MOT task and the speed of the Transition agility test (ps < 0.05, R2 = 0.45). For Passing accuracy, MOT and Backward sprint were found to be significant predictors (ps < 0.05, R2 = 0.40). Additionally, the comprehensive measure of defensive actions (Goals against), was significantly associated with MOT accuracy (*p* = 0.02, R2 = 59.76) but not with any of the ice tests.

**Conclusions**

Spatial attention plays a crucial role in both puck possession and passing, thereby contributing to the overall defensive performance. The role of specific skating skills requires further investigation due to the established role in the possession and passing, but not overall efficiency of defensemen.

**Acknowledgment**

This work was supported by the Ministry of Science and Higher Education of the Russian Federation, (Agreement 075-10-2021-093, Project ISR-RND-2252).

## A73 Does “traditional” sport have a future or is our future e-sport?

### Tvrtko Galić

#### Faculty of Kinesiology, “Josip Juraj Strossmayer” University of Osijek, Osijek, Croatia

##### **Correspondence:** Tvrtko Galić (tvrtko.galic@gmail.com)

*BMC Proceedings 2024*, **18(11)**: A73

**Background**

When sports are mentioned, the first thing that comes to mind is the traditional forms of sports that have been present in society for decades. However, with the increasing development of technology, there are also new forms of sports, the most popular of which is the so-called e-sport, whose popularity we witness every day.

**Materials and methods**

The published data are the result of secondary research of publicly available data from different sources, and the same were then compared to show the differences between individual "traditional" sports and e-sports.

**Results**

Comparing the numbers of individual competitions in "traditional" sports and e-sports will show the extent of the expansion of e-sports. For example, the biggest tournament in e-sports "The International 2021" had a prize fund of 40 million dollars, and Wimbledon, as the most famous tennis tournament, had a prize fund of 35 million dollars. In the same year, the EHF Handball Champions League had a prize fund of around 12 million dollars. In the long term, the numbers are not in favor of “traditional” sports even when comparing the size of the market, the number of fans and the like. The men's final of Wimbledon 2023 was a record and was watched by 11 million people via different platforms, while the final in the League of Legends game was watched by 6.4 million people.

**Conclusions**

Due to the ever-increasing income generated by this type of sport, the increased number of participants and fans, numerous countries have begun to recognize professional e-sports players as athletes, which forces the conclusion that e-sports cannot be ignored in the general context of sports. The task of all people in the sports industry is to find the necessary models of cooperation between "traditional" sports and e-sports, the sport of new generations, in the coming years.

## A74 Effect of integrate training method on core strength and stability in young adult males and females

### Miloš Kojić, Tijana Šćepanović, Borislav Obradović, Vanja Dimitrijević

#### Faculty of Sport and Physical Education, University in Novi Sad, Novi Sad, Serbia

##### **Correspondence:** Miloš Kojić (milos.kojic@fsfvns.edu.rs)

*BMC Proceedings 2024*, **18(11)**: A74

**Background**

This research explores the effects of the Integrative Training Method (IM) over six weeks on the strength and stability of core muscles. Recognized for its comprehensive exercise approach, IM emphasizes overall core muscle strengthening and coordination to enhance body stability. Participants in the study underwent a structured 6-week IM program, with parameters of strength and control assessed for both anterior and posterior core muscles.

**Materials and methods**

This study involved 60 participants, evenly split between 30 males and 30 females, to investigate the effects of a six-week Integrative Training Method (IM) intervention on core strength and control. Initial assessments included anthropometric measurements (height, weight), trunk flexor test, trunk extensor test, right and left side plank tests, and the Double Leg Lowering (DLL) test. Participants underwent a structured six-week IM program, comprising four phases: Inhibition, Stretching, Activation, and Integration. The IM intervention targeted comprehensive core muscle strengthening and coordination. Following the intervention, participants underwent final assessments to evaluate changes in the measured parameters.

**Results**

The study outcomes displayed significant effects across all examined parameters following a six-week Integrative Training Method (IM) program. A notable increase in core strength was observed, as assessed through the trunk flexor test, along with improvements in trunk extensor test. Participants demonstrated substantial progress in both right and left side plank tests, while the DLL test indicated a decrease in the average angle, suggesting enhanced outcomes. It is noteworthy that the results are uniformly favorable for both male and female participants, without statistically significant difference observed between genders. These findings underscore the universal efficacy of the IM program in enhancing core strength and control, irrespective of participants gender.

**Conclusions**

This conclusion highlights the holistic approach of the IM, yielding measurable enhancements in core performance and contributing to a comprehensive understanding of its applicability in training.

## A75 Effects of structured exercise program on the general coordination development of 5–6-year-old children

### Nikola Manolopoulos, Tamara Matijević, Snežana Damjanović

#### Faculty od Sport and Physical Education, University in Novi Sad, Novi Sad, Serbia

##### **Correspondence:** Nikola Manolopoulos (mani1905@yahoo.com)

*BMC Proceedings 2024*, **18(11)**: A75

**Background**

In the context of early childhood education, the initial years of life play a crucial role in the development of children. This study aims to explore the effects of structured exercise programs on enhancing general coordination skills in 5-6-year-olds, promoting physical and cognitive-emotional development for future academic success and the overall well-being of children.

**Materials and methods**

The study assessed coordination quality in 68 participants in the experimental group and 61 in the control group (aged 5-6). The control group followed standard preschool programs, while the experimental group engaged in organized physical activities at the "Feniks" sports school alongside regular preschool attendance. Anthropometric measures, including body height, mass, and BMI, were recorded. Coordination quality was evaluated using the Körperkoordinationstest für Kinder (KTK) test battery, involving tasks like walking backward, moving sideways, hopping for height, and jumping sideways.

**Results**

Repeated measurements analysis revealed significant differences in Jumping sideways (F(1,127)=9.359, *p*<0.01, partial η2 = 0.069) and moving sideways (F(1,127)=4.251, p=0.04, partial η2 = 0.032). Most variables within groups showed progress, except for Walking backward (*p*=0.06) and Moving sideways (*p*=0.08) in the experimental group, attributed to high baseline scores. Despite lacking statistical significance, the experimental group demonstrated noteworthy progress in both tests (*p*=0.06 and *p*=0.08), indicating improvement despite starting with elevated skill levels.

**Conclusions**

The fundamental conclusion of this research is reflected in the fact that a regular physical exercise program, focused on improving basic motor skills and coordination, had an exceptionally favorable impact on this aspect of development in preschool children. Interventions effectively enhanced performance across, although statistical significance was not reached in all cases.

## A76 The importance of communication skills for enhancing the quality of physical education teaching

### Tamara Matijević

#### Faculty of Sport and Physical Education, University in Novi Sad, Novi Sad, Serbia

##### **Correspondence:** Tamara Matijević (tamaramatijevic99@gmail.com)

*BMC Proceedings 2024*, **18(11)**: A76

**Background**

Education shapes comprehensive growth, emphasizing the critical role of teacher-student communication. Effective interaction fosters critical thinking, social skills, and mentorship. In physical education, clear teacher-student communication is pivotal for success, enhancing motivation, goal-setting, and overall instruction quality. Despite its significance, the exploration of communication's impact on physical education remains underexplored, warranting further research to deepen understanding and improve educational processes.

**Materials and methods**

This research employs a descriptive methodology to examine physical education and communication skills. The analysis encompasses primary and secondary sources, utilizing internet search engines such as "Web of Science," "Google Scholar," and "PubMed," with keywords "physical education and communication skills." Selection of works focuses on the period from 2015 to 2023, excluding review articles and meta-analyses to maintain relevance.

**Results**

Students recognize their role in communication quality and value skill improvement. Research emphasizes subtle differences in teacher-student perspectives, showcasing high communication skills among physical education students. Effective communication is crucial in physical education for knowledge transfer, critical thinking, and overall student development.

**Conclusions**

In summary, the research underscores the pivotal role of effective communication skills in education and, specifically, in physical education. Students acknowledging their responsibility and valuing skill improvement, along with the subtle differences in teacher-student perspectives, highlight the importance of fostering robust communication. Recognizing high communication skills among physical education students and emphasizing the critical role of effective communication in knowledge transfer and overall student development. The results highlight the ongoing importance of focusing on communication skills

## A77 Examination of the effects of 8-week two different concurrent training programs on athlete’s performance and physiological parameters

### Birgül Arslan^1^, Salih Pinar^2^, Figen Çiloğlu^3^

#### ^1^Sport Science Faculty, Selcuk University, Konya, Türkiye; ^2^Sport Science Faculty, Fenerbahce University, İstanbul, Türkiye; ^3^Sport Science Faculty, Rumeli University, İstanbul, Türkiye

##### **Correspondence:** Birgül Arslan, bbirgularslan@gmail.com

*BMC Proceedings 2024*, **18(11)**: A77

**Background**

The study examined the effects both of the concurrent training models, which included an eight-week High-Intensity Interval Training (HIIT) and High-Intensity Functional Training (HIFT) with additional strength training (K), on physiological parameters (blood lactate concentration and heart rate), athlete's performance (maximal strength, explosive power, aerobic endurance, 20 m sprint), and biochemical outputs.

**Materials and methods**

The study involved a total of 21 young football players with intermediate level of activity. Participants underwent pre- and post-tests and eight-week training patterns. The participants underwent measurements of body composition, adaptation studies, trial measurements, biochemical blood and urine analyses, aerobic endurance measurements, sprint tests, and strength measurements. The post-training assessments were administered to each participant in the same manner. Only pre- and post-tests were administered to the control group. The data analysis was conducted using SPSS 24. The Mixed Design ANOVA analysis was utilized to examine the performance, physiological, and biochemical data of the participants before and after the experiment. Bonferroni correction was used for multiple comparisons. The significance level was set at *p*<0.05.

**Results**

In male soccer players, VO2max, CMJ, 1-TM bench press and Yo-Yo test were effective in increasing athletic performance with physiological outcomes in recovery processes (HR, LA, AZD) compared to the control group (traditional training). There was no significant difference between the biochemical changes in the K+HIIT, K+HIFT and control groups during the trial period.

**Conclusions**

In summary, both of the concurrent training models had positive effects on sports performance and physiological outcomes, but not on biochemical outcomes.

## A78 Low- and high-resistance training have similar effects on muscle strength and hypertrophy in young adults

### Abdullah Kayhan^1^, Salih Karaman^2^, Halil Özer^3^, Ömer Faruk Topaloglu^3^, İnci Kara^3^, Zübeyde Aslankeser^4^

#### ^1^Faculty of Sports Science, Akdeniz University, Antalya, Türkiye; ^2^Faculty of Sports Science, Hatay Mustafa Kemal University, Hatay, Türkiye; ^3^Faculty of Medicine, Selcuk University, Konya, Türkiye; ^4^Faculty of Sports Science, Selcuk University, Konya, Türkiye

##### **Correspondence:** Zübeyde Aslankeser (zaslankeser@gmail.com)

*BMC Proceedings 2024*, **18(11)**: A78

**Background**

It is well known that high resistance exercise at 65-80% of one repetition maximum (1-RM) should generally be used for muscle hypertrophy and strength development. However, many people who lack the motivation or time to exercise find high resistance strength training impractical. The aim of this study was to investigate the effects of low and high resistance strength training on muscle strength and hypertrophy in healthy young men.

**Materials and methods**

Twenty-two healthy young male volunteers who had training experience before were randomly divided into low (*n*=11) and high (*n*=11) resistance groups. Muscle strength was determined by 1 RM, and muscle cross-sectional area and thickness were measured by ultrasonography (USG). Low and high resistance groups continued 6 weeks of resistance training as 3 days/wk. The load was 30% in the low resistance group and 75% in the high resistance group. Mixed design two-factor analysis of variance with repeated measures was used for all variables of the groups.

**Results**

After training, 1-RM increased significantly in both groups (*p*<0.05). The diameter and area of the rectus femoris (RF) and vastus intermedius (VI) muscles increased in both groups (*p*<0.05). Despite different training intensities, both low and high resistance groups showed similar increases in strength, muscle cross-sectional area.

**Conclusions**

These results have shown that low loads with near-exhaustive repetitions can produce similar gains in strength and muscle cross-sectional area as high loads, thanks to the mechanical and physiological responses they produce in the muscle. Therefore, low-load resistance training can be used to improve strength and muscle hypertrophy.

## A79 Effect of beta alanine supplementation on carnosine and histidine level tissue in rats performing high-intensity interval training

### Duygu Çamkarten^1^, Erbil Harbili^2^, Güzin Özkurt^3^, Ferhan Bölükbaş^4^

#### ^1^Konya Metropolitan Municipality, Konya, Türkiye; ^2^Faculty of Sport Sciences, Selçuk University, Konya, Türkiye; ^3^Faculty of Veterinary Medicine, Aksaray University, Department of Biochemistry, Aksaray, Türkiye; ^4^Faculty of Veterinary Medicine, Aksaray University, Department of Histology and Embryology, Aksaray, Türkiye

##### **Correspondence:** Erbil Harbili (eharbili@selcuk.edu.tr)

*BMC Proceedings 2024*, **18(11)**: A79

**Background**

The primary role of carnosine is to buffer the increased hydrogen ion in the muscle, helping to maintain the acid-base balance. The aim of this study was to investigate the effect of beta-Alanine supplementation on carnosine and histidine levels in rats undergoing high-intensity interval swimming training.

**Materials and methods**

In the study, a 12-week-old Wistar Albino male rat (*n*=30) was used. Rats were divided into training (TG), beta-alanine (BAG), training+beta-alanine (TBAG), and control group (CG), including 6 in the control group and 8 in the research groups. The training groups were given high-intensity interval swimming training 5 days a week for 4 weeks. Beta-alanine supplementation of 250 mg Beta-Alanine per kilogram of body weight was given to the groups taking Beta-alanine by daily gavage method. At the end of 4 weeks, all rats were performed a swimming test until they were exhausted. Blood samples were taken from the animals' hearts immediately after the swimming test. Carnosine, L-histidine, Lactic acid, Bicarbonate and pH levels were measured from the blood samples taken.

**Results**

In this study, it was determined that the swimming test performance of the beta-alanine + training group was higher than the other groups, and the swimming test performance of the training group was higher than the beta-alanine and control groups. It was found that carnosine, L-Histidine and bicarbonate levels did not show significant differences between the groups, while levels of pH and lactic acid showed significant differences between the groups. It was determined that the pH level was lower in the beta-alanine, training and beta-alanine + training groups compared to the control group, and that the lactic acid level was higher in the training group than in the control group.

**Conclusions**

It was observed that beta alanine supplementation had an effect on the pH level, but did not increase carnosine, L-histidine and bicarbonate levels.

## A80 The association between rapid weight loss and dietary habits of former combat sports athletes

### Stephanie Mić, Saša Krstulović, Goran Kuvačić

#### Faculty of Kinesiology, University of Split, Split, Croatia

##### **Correspondence:** Goran Kuvačić (goran.kuvacic@kifst.eu)

*BMC Proceedings 2024*, **18(11)**: A80

**Background**

Weight reduction is common among athletes with the goal of improving the strength-to-mass ratio, locomotor efficiency, or aesthetic appearance. Athletes typically reduce food and fluid intake during periods of weight reduction but sometimes employ aggressive methods of rapid weight loss (RWL) that can be harmful to health. This research aimed to determine the association between RWL and the dietary habits of former combat sports athletes.

**Materials and methods**

A modified version of the Combat Sports Post-Career Health Questionnaire (CSPCHQ) was applied, isolating variables to identify RWL methods used by participants during their sports careers and their dietary habits after completing their sports careers. Dietary habits were assessed using the Starting the Conversation (STC) scale, Healthy Eating Assessment (HEA), and Disinhibition scale from the Mindful Eating Questionnaire (MEQd). The total sample consisted of 70 former male combat sports athletes with an average age of 41.6±9.1 years. Pearson's correlation coefficient and coefficient of determination (R2) were used to establish the relationship between variables.

**Results**

The results indicate that the surveyed athletes moderately reduced body weight on average during their sports careers. It was also observed that, after retirement, they mainly consumed a healthy diet. Correlation analysis did not reveal a statistically significant association between RWL methods and any scale assessing dietary habits (*p* > 0.05). These findings align with some of the previous research, where frequent weight reduction during sports careers did not have a specific impact on the body mass index (BMI) in retirement for elite athletes, regardless of the RWL methods practiced during their careers.

**Conclusions**

In the analyzed sample, no association was found between the RWL methods used during sports careers and dietary habits after retirement. Future research should encompass a larger number of athletes and analyze differences among individual sports.

## A81 Effects of vibrational treatment on body composition and weight loss: A systematic review

### Maja Radovanović, Frane Žuvela, Goran Kuvačić

#### Faculty of Kinesiology, University of Split, Split, Croatia

##### **Correspondence:** Goran Kuvačić (goran.kuvacic@kifst.eu)

*BMC Proceedings 2024*, **18(11)**: A81

**Background**

Vibration as an exercise modality has garnered considerable interest in health and sports science due to its potential therapeutic benefits. With its growing popularity in health and sports centers, vibrational therapy presents an innovative approach to health and fitness. This study used systematic literature search methods to summarize existing research on the effects of whole-body vibration training (WBVT) on body composition and weight loss.

**Materials and methods**

A systematic review followed PRISMA guidelines, involving a detailed search in Web of Science and Scopus databases. The focus was on evaluating the effects of whole-body vibration on body composition and weight loss. The searches were performed using the following strings: (“whole-body vibration” OR “vibration training” OR “vibration exercise”) AND (“body composition” OR “weight loss”); (“whole-body vibration” AND “body composition”); (“whole-body vibration” AND “weight loss”).” Studies were selected based on the PICO framework, ensuring a comprehensive and relevant analysis.

**Results**

From an initial pool of 277 studies, 12 met the inclusion criteria. The analysis revealed that vibrational training significantly influences body composition while showing limited efficacy in weight loss. Key findings include a reduction in body fat percentage and an increase in lean muscle mass in certain cases, highlighting the potential metabolic health benefits. Moreover, a significant and consistent increase in muscle strength was observed across various age groups and fitness levels, indicating the wide applicability of vibrational training as a strength-building intervention.

**Conclusions**

Although not markedly effective in reducing overall body weight, vibration training significantly improves muscle strength across diverse population samples. This training modality is particularly beneficial for older adults, offering a viable exercise option without the need for conventional dynamic exercises. The findings highlight vibrational training's potential as a supplementary modality in fitness and therapy, emphasizing the need for more research on its effects in diverse demographics.

## A82 The effect of group exercise training on physical performance in young healthy women

### Josip Cvenić^1^, Stefan Mijalković^2^, Nera Ivković^1^

#### ^1^Faculty of Kinesiology, “Josip Juraj Strossmayer” University of Osijek, Osijek, Croatia; ^2^Faculty of Sport and Physical Education, University of Niš, Niš, Serbia

##### **Correspondence:** Josip Cvenić (jcvenic@kifos.hr)

*BMC Proceedings 2024*, **18(11)**: A82

**Background**

Physical activity has a positive effect on a number of health outcomes. It is a concept that provides protection or improvement in physical fitness parameters such as repetitive, personalized, muscle strength, endurance, flexibility, and agility. The main objective of this study is to determine changes in morphological characteristics and motor abilities of young women after participating in group fitness programs over a period of 12 weeks.

**Materials and methods**

The sample consisted of 20 young women with an average age of 25 years and 6 months, who have been engaged in the fitness training for an average of 2.5 years. After obtaining general demographic information from participants, the physical performances of the individuals were measured at the beginning and end of period. The information recorded for the individuals participating in the study included age, height, weight, seven girth measurements (waist 1 and 2, arm flexed and relaxed, forearm, thigh, calf) and four strength fitness tests (sit-ups, squat test, push-ups, pull-ups).

**Results**

Results obtained statistically significant differences (*p*<0.05) in variables such as waist circumference, thigh circumference, and arm circumference during flexion and contraction. Additionally, significant statistical differences were observed in all tests that represents repetitive strength of upper body, core, and lower body. These results indicate a positive impact of group fitness training on the physical characteristics and abilities of the participants in a very short period.

**Conclusions**

This study examined the effect of group exercise training on morphological circumferences and physical performance in healthy young women. Exercise training in 12 weeks increased physical performance parameters such as repetitive strength and decreased girth measurements that can be used in determining body size and composition.

## A83 The effect of different rest intervals on repeated sprint performance and hormones in young football players

### Hacı Servet Karaca^1^, Sultan Harbili^2^, Erbil Harbili^2^

#### ^1^Provincial Directorate of Youth and Sports, Konya, Türkiye; ^2^Selçuk University, Faculty of Sport Sciences, Konya, Türkiye

##### **Correspondence:** Sultan Harbili (sharbili@selcuk.edu.tr)

*BMC Proceedings 2024*, **18(11)**: A83

**Background**

This study aimed to investigate the effects of repeated sprints applied at different rest intervals on sprint performance, growth hormone (GH), total testosterone (TT), cortisol, and insulin hormones in young football players.

**Materials and methods**

Twelve young male soccer players between the ages of 14-16 participated in the study. The 6x35 m sprint test with rest intervals of 10 and 30 seconds was applied with intervals of 48 hours. Repeated sprint times and anaerobic power outputs were calculated in both rest intervals. Heart rate (HR) and blood lactate (LA) were measured before the test, at the end of the test, at 5 and 15 minutes after the test, and hormone measurements were measured before the test, at the end of the test, and 1 hour after the test.

**Results**

The 30-second rest interval repeated sprint performance was higher than the 10-second rest interval performance (*p*<0,01). Anaerobic power output in the 30-second rest interval was higher than that of a 10-second rest interval (*p*<0.05), however, the fatigue index was lower (*p*<0,01). While the effect of the time factor was significant on LA and HR (*p*<0,05), the effect of the rest interval on LA and HR was not significant (*p*>0,05). Rest interval did not affect growth and insulin hormones (*p*>0,05), and time-dependent changes were observed in these hormones (*p*<0,01). The effect of both rest interval and time factors was significant on testosterone hormone (*p*<0,05). It was observed that both the rest interval and the time factor had no significant effect on the cortisol hormone (*p*>0,05).

**Conclusions**

It was observed that different rest intervals were effective on repeated sprint test times and anaerobic power outputs but did not affect LA and HR. The rest interval was effective on the testosterone hormone level.

## A84 The relation between physical activity and the quality of life in elderly people

### Stefan Mijalković, Anđela Đošić, Danijela Živković, Mladen Živković, Saša Pantelić

#### Faculty of Sport and Physical Education, University of Niš, Niš, Serbia

##### **Correspondence:** Stefan Mijalković (stefimijalkovic@gmail.com)

*BMC Proceedings 2024*, **18(11)**: A84

**Background**

United Nations data indicates a 2% increase in the number of elderly people between 1950 and 2000, with projections estimating that the elderly will make up 22% of the total population by the year 2050. Simultaneously, the rise in elderly population reduces overall physical activity, impacting quality of life parameters. The aim of this study was to determine whether the level of physical activity was related to the quality of life in elderly men and women.

**Materials and methods**

An assessment of physical activity and quality of life was conducted on a sample of 614 elderly women and 666 elderly men (65+ years), using a set of 8 variables for physical activity and 4 variables for quality of life. The level of physical activity was assessed using the IPAQ questionnaire, while the quality of life was evaluated using the World Health Organization questionnaire (WHOQoL-bref). Canonical correlation analysis was conducted to identify relations, separately for elderly men and elderly women (*p*< 0.01).

**Results**

The study’s results indicated that significant relations were found between leisure-time physical activity and overall moderate physical activity with the environmental domain (*p* = 0.000). Additionally, relations were observed between transportation-related physical activity and overall walking activity with physical and mental health, as well as social relations (*p* = 0.003). Significant canonical relations were identified in elderly women between overall walking, overall moderate physical activity and overall physical activity with mental health, social relationships, and the environment (*p* = 0.000). Additionally, relations were found between occupational physical activity, household physical activity, overall moderate physical activity, and physical health (*p* = 0.011).

**Conclusions**

This study confirmed that different domains of physical activity were related to the quality of life in elderly people. It found a direct relation between quality of life and physical activity levels, with variations between genders.

## A85 The effects of different modern dance programs on development of physical fitness related to health in younger school aged girls

### Aleksandra Ilić^1^, Romina Herodek^2^, Saša Jovanović^3^

#### ^1^Faculty of Sport and Physical Education, University of Novi Sad, Novi Sad, Serbia; ^2^Faculty of Sport and Physical Education, University of Niš, Niš, Serbia; ^3^Faculty of Sport and Physical Education, University of Banja Luka, Banja Luka, Bosnia and Herzegovina

##### **Correspondence:** Aleksandra Ilić (play.dance.studio@gmail.com)

*BMC Proceedings 2024*, **18(11)**: A85

**Background**

The aim of this study was to determine the effects of experimental modern dance programs on the development of physical fitness related to health in young school-aged girls.

**Materials and methods**

The total sample included 100 female subjects aged 9 to 11 years divided into three groups. The control group (*N*=50) included female pupils of the elementary school, the experimental group 1 (*N*=25) included girls who have been involved in the modern dance program for no longer than one year, the experimental group 2 (*N*=25) included girls who have been involved in the modern dance program for at least two years. The experimental treatment was longitudinal in nature, lasting six months, 3 times a week for the experimental group 1 and 5 times a week for the experimental group 2. The extended ALPHA battery of tests was utilized in this investigation: Body Mass Index (BMI), Waist circumference, Skinfold thickness (triceps and subscapular skinfold), Handgrip strength, Standing long jump, 4x10m shuttle run test, 20-meter shuttle run test.

**Results**

The results of the multivariate analysis of covariance indicates that there are statistically significant differences (*P*=0.00) between the participants of different groups in the tests of physical fitness related to health. By applying the univariate analysis of covariance, it was observed that these differences are present in the variables Standing long jump test, 4x10m shuttle run and 20-meter shuttle run test on the level of statistical significance of *p*=0.00. The best effect was achieved by experimental group 1 in the Standing long jump test, and by experimental group 2 in the 4x10m shuttle run and 20-meter shuttle run test.

**Conclusions**

Based on the evaluation of the results, we can conclude that modern dance treatment significantly influences the improvement of physical fitness related to health in children of younger school age.

## A86 Examination of physical activity and the use of smart devices among Teacher Training Students in Vojvodina

### Anita Štajer

#### Hungarian Language Teacher Training Faculty, University of Novi Sad, Subotica, Serbia

##### **Correspondence:** Anita Štajer (stajerani@gmail.com)

*BMC Proceedings 2024*, **18(11)**: A86

**Background**

Physical activity and the use of smart devices have become a very timely research topic since the quality and the quantity of these two variables greatly influence body composition which affects health and performance.

**Materials and methods**

The aim of this research was to investigate the prevalence and patterns of smart device activity and physical activity among Teacher Training students in Vojvodina. 111 university students (average age: 21,4 years ± 3,4; women: *n* = 108, men: *n* = 3) from Subotica participated in the survey on a voluntary basis and filled in the questionnaire related to the assessment of physical activity and smart device use.

**Results**

Average weight is 63.21 kg, and the average BMI is 23.46 kg/m^2^. The correlation results show a reverse and negative association (*r*=-2.49; *p*=0.009) between physical activity and BMI. Students with normal BMI data move significantly more than their peers with higher BMI data. There was no correlation between smart device use and BMI values, although the data shows that students with higher BMI values spend slightly more time in front of the screen. The test results show that 61.3% of them are not engaged in any type of physical activity. 37.8% use their phone and 27% use computer for more than 10 hours during the week.

**Conclusions**

Students should be encouraged to exercise and lead a healthier lifestyle. After all, they are not only responsible for their own health. They are also going to be important role models for their future students. Therefore, the task to become a positive role model is one that all educators should take seriously.

## A87 mRNA gene expression of healthy young participant who consumed functionally enriched food

### Petar Šušnjara^1,2,3^, Nikolina Kolobarić^1,2^, Ana Stupin^1,2^, Zrinka Mihaljević^1,2^, Ines Drenjančević^1,2^

#### ^1^Institute and Department of Physiology and Immunology, Faculty of Medicine Osijek, “Josip Juraj Strosssmayer” University of Osijek, Osijek, Croatia; ^2^Scientific Center of Excellence for Personalized Health Care, “Josip Juraj Strossmayer” University Osijek, Osijek, Croatia; ^3^Faculty of Kinesiology Osijek, “Josip Juraj Strosssmayer” University of Osijek, Osijek, Croatia

##### **Correspondence:** Petar Šušnjara (psusnjara1@gmail.com)

*BMC Proceedings 2024*, **18(11)**: A87

**Background**

Dietary supplementation with compounds that possess antioxidant and anti-inflammatory properties (n-3 polyunsaturated fatty acids (PUFAs), selenium, vitamin E, lutein), has been shown to positively correlate with improvements in chronic conditions, although understanding of these combined effects in healthy humans is limited. We hypothesized that a three-week diet containing enriched eggs can change the relative gene expression of mRNA.

**Materials and methods**

This was a randomized, double-blind, placebo-controlled interventional study (part of ID: NCT04564690). Control group (*n*=14) consumed regular hen eggs (1.785 mg of vitamin E, 0.330 mg of lutein, 0.054 mg of selenium and 438 mg of n-3 PUFAs daily), while Nutri4 group (*n*=20) consumed enriched eggs (3.29 mg of vitamin E, 1.85 mg of lutein, 0.06 mg of selenium and 1026 mg of n-3 PUFAs daily), 3 eggs/day for 3 weeks. mRNA expression of GPx1, GPx4, SOD2, iNOS, nNOS and eNOS was determined with real time PCR (BioRad, CFX96 Real – Time System) from stored peripheral blood mononuclear cells (PBMC) samples of participants before and after dietary protocol. Results were normalized to GAPDH housekeeping gene. Oneway ANOVA was conducted for statistical analysis with Tukey post hoc test; *p*<0.05 was considered statistically significant. Statistical analysis: paired t-test with Tukey's post hoc test; *p*<0.05 was considered statistically significant.

**Results**

nNOS of mRNA gene expression are increased in Nutri4 group after diet, while other parameters remained unchanged.

**Conclusions**

Increased intake of certain nutrients through functionally enriched foods, such as n-3 PUFA, vitamin E, lutein and selenium can have various beneficial effects such as increased mRNA gene expression of nNOS in PBMC after a three-day dietary protocol in Nutr4 group

## A88 Match running performance preceding scoring and conceding a goal in professional soccer

### Marek Konefał^1^, Błażej Szmigiel^1^, Bogdan Bochenek^2^, Ryland Morgans^3^, Piotr Żmijewski^4^

#### ^1^Department of Human Motor Skills, Wrocław University of Health and Sport Sciences, Wrocław, Poland; ^2^Institute of Meteorology and Water Management, National Research Institute, Warszawa, Poland; ^3^School of Sport and Health Sciences, Cardiff Metropolitan University, Cardiff, United Kingdom; ^4^Department of Biomedical Sciences, Józef Piłsudski University of Physical Education in Warsaw, Warszawa, Poland

##### **Correspondence:** Marek Konefał (marek.konefal@awf.wroc.pl)

*BMC Proceedings 2024*, **18(11)**: A88

**Background**

The final result of the match depends on short moments of the game in which the team scores or concedes a goal. These moments are contextually extremely important and worthy of detailed analysis. Therefore, this study aimed to investigate the potential differences in the match running performance of professional soccer players five minutes before scoring and conceding a goal in the Polish Ekstraklasa.

**Materials and methods**

The sample consisted of 278 matches with 570 goals scored during official matches of the 2022/23 Polish Ekstraklasa season. All data was collected utilizing the computerized multiple-camera optical tracking system TRACAB. Total distance covered (TD), standing distance (StD; < 0.72 km∙h-1), walking distance (WD; 0.73-7.2 km∙h-1), jogging distance (JG; 7.21-14.4 km∙h-1), running distance (RD; 14.41-19.8 km∙h-1), high-speed running distance (HSR; 19.81-25.2 km∙h-1) and sprinting distance (SprD; >25.2 km∙h-1) were analyzed in 5-min intervals prior to a goal scored for both competing teams.

**Results**

The employed linear mixed models showed that all examined metrics of match-running performances were higher in teams that scored goals compared to teams that conceded goals. Within five minutes before scoring a goal in Polish Ekstraklasa matches, the scoring team had significantly greater TD (∆ 95%CI: 256.8-300.4 m; *p*=0.001), WD (∆ 95%CI: 52.3-95.8 m; *p*=0.001), JG (∆ 95%CI: 100.5-144.1 m; *p*=0.001) and RD (∆ 95%CI: 16.2-59.8 m; *p*=0.001) compared to the opponent, although no differences were found for HSR and SprD.

**Conclusions**

These results demonstrate the enhanced identification potential of key physical factors influencing match outcome in the Polish Ekstraklasa, thereby optimizing the training process, and improving overall performance. To enhance the effectiveness of soccer training, coaching and performance staff should consider the study's findings, that indicate an increase in the volume of medium- and low-intensity running efforts preceding a goal.

## A89 The influence of developmental gymnastics on the motor coordination of preschool children

### Zoran Milić^1^, Anita Štajer^2^, Milan Šolaja^3^, Darijan Ujsasi^4^, Vladan Pelemiš^5^

#### ^1^College for Vocational Education of Preschool Teachers and Coaches, Subotica, Serbia; ^2^Hungarian Language Teacher Training Faculty, University of Novi Sad, Subotica; ^3^Faculty of Sports and Psychology, Educons University, Novi Sad, Serbia; ^4^High School for Police, Sremska Kamenica, Serbia; ^5^Faculty of Education, University of Belgrade, Belgrade, Serbia

##### **Correspondence:** Zoran Milić (zoranmilic@yahoo.com)

*BMC Proceedings 2024*, **18(11)**: A89

**Background**

Developmental gymnastics contributes to the improvement of the motor development of preschool children. Sensitive periods in child development are extremely important for the development of motor abilities.

**Materials and methods**

We compared the distribution of frequencies of body mass index and motor competence levels (“severe motor disorder” (MQ 56-70,), “moderate motor disorder” (MQ 71-85,), “normal” (MQ 86-115,), “good” (MQ 116-130,) and “high” (MQ 131-145,) among the groups. Mixed-model ANCOVA was used to evaluate the intervention effects on the KTK test outcomes while controlling for baseline age (mean=6.28) and body mass index (mean=15.32) effects. Eta squared and partial eta squared are reported as the measures of effect size for the simple and main effects, and interaction effects, respectively, and defined as small (0.01), medium (0.06), and large (0.14). The level of significance was set at p≤0.05. The purpose of the study was to investigate and analyze the impact of developmental gymnastics on motor coordination, through two intervention modules. The sample included 176 children divided into three sub-groups: experimental group E1-45, experimental group with additional exercises E2-45, and control group K-86. A 10-week experimental exercise treatment was applied to two groups of participants, one of which had one additional exercise at home with their parents.

**Results**

The results indicate that additional exercise at home had greater effect only on the Jumping sideways (F(1,84)=6.47, *p*=0.01, ŋ_*p*^2=0.07) as compared to the intervention alone. Nevertheless, additional exercise at home did not elicit effects to a greater extent on the Single-lever jumps (F(1,84)=2.52, *p*=0.12, ŋ_*p*^2=0.03), Moving sideways (F(1, 84)=0.59, *p*=0.44, ŋ_*p*^2=0.01), and MQ total (F(1, 84)=0.17, *p*=0.68, ŋ_*p*^2=0.00).

**Conclusions**

It can be concluded that developmental gymnastics applied to children of the mentioned age had positive effects on improving coordination, and that the intervention gave positive and expected results.

## A90 Does reaction time influence countermovement jump performance in basketball players? Pilot study

### Jelena Aleksić^1^, Lucija Faj^2^, Branislav Božović^13^, Anastasija Kočić^1^, Olivera M. Knežević^1^, Dimitrije Čabarkapa^3^, Damjana V. Čabarkapa^3^, Dragan M. Mirkov^1^

#### ^1^Faculty of Sport and Physical Education, University of Belgrade, Belgrade, Serbia; ^2^Faculty of Kinesiology Osijek, “Josip Juraj Strosssmayer” University of Osijek, Osijek, Croatia; ^3^Jayhawk Athletic Performance Laboratory – Wu Tsai Human Performance Alliance, Department of Health, Sport and Exercise Sciences, University of Kansas, Lawrence, Kansas, United States of America

##### **Correspondence:** Jelena Aleksić (jelena.aleksic@fsfv.bg.ac.rs)

*BMC Proceedings 2024*, **18(11)**: A90

**Background**

The countermovement vertical jump (CMJ) plays a fundamental role in differentiating various neuromuscular performance parameters of athletes. This pilot study examined some of the metric characteristics of a modified test, which, in addition to jump height and take-off time, includes reaction time metrics. Given the satisfactory reliability in the preliminary results of a larger study, this study aims to explore whether implementing this modified test will affect CMJ performance in basketball players.

**Materials and methods**

The sample included 10 healthy basketball players who performed 3 CMJ trials without and with reaction time. Visual stimulus was randomly displayed on a computer screen 2m away from the subject. Subsequently, video analysis was conducted using Kinovea (0.9.5) to obtain CMJ metrics during take-off and landing phases of the jump. Paired-samples t-tests were used to compare the differences in all CMJ metrics without and with reaction time.

**Results**

Significant differences were observed during take-off (*p*<0.05) in time of eccentric (*t*= 4.214) and concentric (*t*= 2.514) phases, total time to take-off (*t*= 4.379), and hip, knee, and ankle flexion angles (*t*= -3.048, *t*= -3.167, *t*= -2.449, respectively), as well as in reactive strength index modified (*t*= -2.845, *p*=0.019).

**Conclusions**

Despite no significant differences observed in jump height as a measured outcome, the introduction of a visual cue altered the execution strategy, with basketball players achieving comparable heights by shortening both eccentric and concentric phases and adjusting their kinematic pattern. This study substantiates findings from prior research, emphasizing the adaptability of control mechanisms for generating power output under different conditions. Integrating an additional task in CMJ evaluation could refine the assessment of sport-specific jumping abilities and enhance testing protocols.

## A91 Application of sport psychology methodology to optimise the self-confidence of a 19-year-old athlete

### Levente Szantai, Josip Lepeš

#### Gál Ferenc University, Szeged, Hungary

##### **Correspondence:** Levente Szantai (szantaipszichologus@gmail.com)

*BMC Proceedings 2024*, **18(11)**: A91

**Background**

The aim of my case is to treat a 19-year-old athletze's self-confidence problem using sport psychology methodology. The football player was regularly criticized by his coach during the first period of the sports psychology counselling process, so the focus was on solving the related difficulties, optimizing his self-confidence. His coach then dropped him from the first team squad and he was transferred to the second team. The process of sports psychology counselling then focused on how to function effectively in the new team and on future orientation.

**Case report**

Different psychodiagnostics tools have also shown the negative effect of the athlete's conflict with the coach and its negative impact on self-confidence. During this period, he was described as having a rigid style of play on the field, as a result of his performance anxiety. Accordingly, the first stage of the sport psychology process was focused on filtering out developmental information, solving the anxiety caused by the presence of the coach and exploring and reinforcing the skills necessary for effective performance. Mental mistake correction after a player's move to the second team has helped to improve effective sport performance and strengthen the athlete's identity. In the final stage of the process, the athlete learned goal setting and assertive communication to concretize future orientation.

**Conclusion**

In summary, the process of sports psychology counselling has proven to be effective in optimizing and then developing the athlete's self-confidence. Furthermore, the client has experienced a significant emotional development, experiencing the challenges of adult football, learning new coping strategies, supporting healthy personality development. The client gave informed consent to have his details published in an open access journal.

## A92 Educational, upbringing and health-improving significance of Kazakh national games

### Yerlan Seisenbekov, Rysgul Kokebayeva

#### Department of Physical Education and Sport, Abai Kazakh National Pedagogical University, Almaty, Kazakhstan

##### **Correspondence:** Yerlan Seisenbekov (yerlan_fks@mail.ru)

*BMC Proceedings 2024*, **18(11)**: A92

**Background**

The life experience demonstrates the significant role of Kazakh national games in the upbringing of the younger generation within general education institutions. When school-age youth engage in lessons related to these games, they learn the art of play, refine their skills through practice, and internalize them through repetition. This fosters a sense of citizenship and patriotism, instilling a love for their nation as they transition into adulthood. However, to effectively integrate Kazakh national games into physical education classes, it is imperative to tailor teaching methodologies to align with local and regional peculiarities, ensuring the games remain engaging for middle school students. Innovative approaches and instructional materials, such as visually appealing aids adorned with national motifs, can captivate children's attention and facilitate learning. Moreover, disseminating educational literature glorifying national games and organizing open classes using modern technology can promote Kazakh cultural values, particularly among urban populations undergoing cultural transitions.

**Case report**

Academic institutions can establish a normative practice over time by incorporating national games into educational curricula and adapting them to students' developmental stages. Considering the sensitivity of developmental periods in physical attributes, selecting games and exercises that align with the peaks of motor skill acquisition to solidify and refine the curriculum effectively is essential. Kazakh national games, imbued with moral values and cultural essence, serve as a conduit for holistic character development, enriching the educational experience.

**Conclusion**

Harmoniously integrating these games into education requires a nuanced understanding of students' psychological predispositions and the quest for knowledge during each lesson. Therefore, a pedagogical approach grounded in psychological insights is indispensable for fostering a deep appreciation and mastery of Kazakh national games among students.

## A93 Rapid weight loss and its impact on skeletal and cardiac muscle biochemical markers in wrestlers: Implications for combat sports

### Carlo Rossi^1^, Roberto Roklicer^2^, Antonino Bianco^1^

#### ^1^Sport and Exercise Sciences Research Unit, University of Palermo, Palermo, Italy; ^2^Faculty of Sport and Physical Education, University of Novi Sad, Novi Sad, Serbia

##### **Correspondence:** Carlo Rossi (carlo.rossi@unipa.it)

*BMC Proceedings 2024*, **18(11)**: A93

**Background**

Restrictive diets, forced fasting, and voluntary reduction of body mass have attracted the attention of scientists, health professionals, and athletes. Reducing weight too quickly is unsustainable and can lead to numerous complications. Short-term body weight regulation is prevalent in combat sports, where rapid weight loss (RWL) methods are employed by athletes in the week leading up to competition, resulting in an average loss of 5% of body weight. This study aimed to examine the effects of rapid weight reduction on changes in biochemical markers of skeletal and cardiac muscle in wrestlers.

**Materials and methods**

The study involved an initial measurement (IM) and two experimental phases. The experimental treatment, following the initial measurement, occurred in two phases to investigate the acute effects of weight reduction on biochemical markers when 5% of body weight was reduced in conjunction with sport-specific training (Phase 1 - P1) compared to changes caused solely by high-intensity sport-specific training without weight reduction (Phase 2 - P2).

**Results**

The application of rapid weight loss together with specific high-intensity training caused statistically significant changes in the markers examined compared to IM. However, when only high-intensity sport-specific training was applied, a change in the activity of the tested markers was still observed, although to a lesser extent than when combined with RWL.

**Conclusions**

For the well-being and safety of athletes, it is advisable to consider interventions such as introducing modifications to competition rules in combat sports categorized by weight classes. This would contribute to addressing the potential risks associated with rapid weight loss strategies in the context of combat sports, emphasizing the need for a balanced approach to athlete health and performance.

## A94 A comparative study on optimizing strength training protocols in young females: Velocity-based vs. percentage-based programs

### Marko Manojlović^1^, Isidora Vasiljević^1^, Carlo Rossi^2^, Tatjana Trivić^1^, Antonino Bianco^2^

#### ^1^Faculty of Sport and Physical Education, University of Novi Sad, Novi Sad, Serbia; ^2^Sport and Exercise Sciences Research Unit, University of Palermo, Palermo, Italy

##### **Correspondence:** Isidora Vasiljević (isidora.vasiljevic@outlook.com)

*BMC Proceedings 2024*, **18(11)**: A94

**Background**

The purpose of this study was to compare the effects of velocity-based strength training (VBT) and percentage-based strength training (PBT) on absolute strength, explosive power, speed, and agility, as well as markers of muscle damage after 6 weeks of exercise programs and a total of 12 training sessions.

**Materials and methods**

The study included 30 subjects divided into three groups of 10 respondents: VBT, PBT, and control. Before and after the experimental treatment, the subjects performed various neuromuscular tests, including one-repetition maximum (1RM) squat exercise and 1RM bench press for absolute strength, squat jump (SJ) and countermovement jump (CMJ) for explosive strength, 5-meter and 20-meter run tests for speed, as well as 505L and 505D tests for agility. Creatine kinase and creatine kinase isoenzyme were analyzed as markers of muscle damage before and after the first training session and 24 hours after the last training session.

**Results**

All implemented neuromuscular performance tests revealed no statistically significant differences between groups. Furthermore, no significant differences were found between the groups regarding the examined markers of muscle damage across all phases. The velocity-based and percentage-based strength training groups showed significant improvement in 1RM squat exercise and 1RM bench press, but a greater enhancement was observed in the VBT group. Furthermore, VBT induced substantial changes in SJ and CMJ height, while PBT only improved in the SJ test. In the control group, no differences between baseline and follow-up measurements were observed in all tests.

**Conclusions**

Although no difference was found between groups, VBT induced greater absolute strength and explosiveness improvements than traditional training and can be considered more effective.

## A95 Quality of life of Montenegrin female students

### Milena Mitrović

#### Faculty for Sport and Physical Education, University of Montenegro, Nikšić, Montenegro

##### **Correspondence:** Milena Mitrović (milenam1054@gmail.com)

*BMC Proceedings 2024*, **18(11)**: A95

**Background**

Quality of life represents an individual's perception of his own position in life in the context of the culture and value system in which he lives, as well as in relation to his own goals, expectations, standards and interests. Quality of life is a multidimensional concept that includes physical, mental, social and behavioral components and functions.

**Materials and methods**

The sample of respondents consisted of 128 female students with an average age of 20.9 years of basic and master's studies from the Faculty of Philosophy and Philology from Niksic. The main instrument was the WHOQOL BREF assessment scale for the assessment of quality of life in the last two weeks. Descriptive statistics were used for data processing.

**Results**

The results showed that female students are moderately satisfied with the quality of life, while they are very satisfied with their physical health. When it comes to mental health, the answers show that female students are satisfied with it on average or slightly above average, except when it comes to negative feelings, which were at a relatively low level in the previous two weeks. They are also moderately satisfied with their personal social relationships.

**Conclusions**

From the above, it can be seen that female students from Montenegro are generally satisfied with the quality of life. This can be justified by the fact that other factors (academic stress, economic status), can play an important role in satisfaction with the quality of life. It’s necessary to additionally determine factors that affect satisfaction with the quality of life, and to improve those that are dependent on system, in order to provide young people with the best possible life.

## A96 Indoor athletic performance evaluation through real-time wireless heart rate monitoring

### Ahmet Aydın^1^, Kerem Tuncay Özgünen^2^, Cemil Keskinoğlu^1^, Emine Nur Talib^1^, Nazlı Yaren Dağ^1^, Ertuğrul Gezgin^2^

#### ^1^Department of Biomedical Engineering, Çukurova University, Adana, Türkiye; ^2^Department of Physiology, Çukurova University, Adana, Türkiye

##### **Correspondence:** Ahmet Aydın (aaydin@cu.edu.tr)

*BMC Proceedings 2024*, **18(11)**: A96

**Background**

The Conconi test is one of the most popular methods to determine aerobic and anaerobic thresholds. This test has limitations, such as the need for a broad measurement space and being able to evaluate only a limited number of athletes simultaneously. To overcome these limitations, a system that can be used to assess athletic performance in an indoor environment was designed. With the developed system, simultaneous and real-time heart rate recordings can be obtained from many athletes in an indoor environment.

**Materials and methods**

In this study, the performance evaluation of 6 participants with different fitness levels (age: 31.3 ± 8.2 years, height: 178.2 ± 5.0 cm, and body weight: 77.3 ± 8.4 kg) was performed on a treadmill (LifeFitness CLST, USA). The test started at 8 km/h and increased by 1 km/h for every 200 meters. The athletes' heart rates were collected from the chest strap with the designed microcontroller-based (ESP32) system and wirelessly transmitted to the PC using ESP-Now. The transmitted data is monitored and recorded in real-time with the developed software. Finally, the anaerobic threshold values were calculated from the recorded data as described with the Conconi protocol.

**Results**

The performance of six athletes with different fitness levels is evaluated with the designed system. The anaerobic speed and heart rate thresholds are obtained as 16 km/h and 196 bpm and 13 km/h and 189 bmp for the best-performing and worst-performing athletes, respectively.

**Conclusions**

In this study, a system that can be used for indoor performance evaluation of athletes were designed. With this system, simultaneous heart rate is collected from multiple athletes wirelessly. The anaerobic thresholds of athletes are calculated, and their performance is evaluated easily in a small indoor area.

**Acknowledgment**

This work is supported by the Scientific and Technological Research Council of Türkiye under Grant 121E648.

## A97 The differences in anthropometric characteristics of young football players based on their positions

### Stefan Maričić, Vladimir Šipka, Filip Sadri, Milorad Jakšić

#### Faculty of Sport and Physical Education, University of Novi Sad, Novi Sad, Serbia

##### **Correspondence:** Stefan Maričić (stefan.maricic@fsfvns.edu.rs)

*BMC Proceedings 2024*, **18(11)**: A97

**Background**

Anthropometric characteristics may be very good predictors and indicators of success and future playing level in football, especially in youth. The present study aims to investigate how anthropometric characteristics and body mass index (BMI) differ from the playing positions of young football players.

**Materials and methods**

The research involved the participation of football players from the youth category of the football club Vojvodina from Novi Sad. The sample consisted of a total of 147 young football players (age: 16.12 ± 1.29 years). They were divided into four positional categories: offensive players (*n* = 33), midfielders (*n* = 33), defensive players (*n* = 46), and goalkeepers (*n* = 13). All subjects were healthy and voluntarily participated in the research. The anthropometric characteristics were assessed through the following measurements according to the recommendations: body height, body mass, various skinfolds (triceps, subscapular, supraspinal, lower leg), elbow and knee diameter, calf, and upper arm muscle circumference. BMI is calculated through the standardized formula.

**Results**

Significant findings emerged in the multivariate analysis of variance (*P*=0.04), particularly in the supraspinal skinfold (*p*=0.03), knee diameter (*p*=0.03), and calf muscle circumference (*p*=0.04) when assessing the different positional categories and their corresponding anthropometric measurements. Goalkeepers obtained higher results in supraspinal skinfold in comparison with midfielders. Offensive players have greater knee diameter and calf muscle circumference compared to midfielders. However, it is important to note that statistical significance was not observed (*p*>0.05) in the remaining variables.

**Conclusions**

Football players of youth categories, in terms of player position specifics, presented significant differences in some of the measured anthropometric characteristics. Thus, these findings can be important for coaches and fitness trainers involved in football training in the context of physical fitness assessment and talent identification.

## A98 The physical aspects of Tiqui-Taca: One of the keys to success in modern football?

### Iason Vasileiadis

#### Department of Physical Education and Sport Science, Democritus University of Thrace, Komotini, Greece

##### **Correspondence:** Iason Vasileiadis (jvasiliadis20@gmail.com)

*BMC Proceedings 2024*, **18(11)**: A98

**Background**

Soccer is maybe the most popular sport in the world and at the same time one of the most physically demanded ones as players are obliged to play a great number of games in one season, with recovery breaks being short and most times insufficient. While this case is already a difficult case for most coaches and participants, the game style and the tactical plan of a team can always make things more complicated and difficult, as teams who aim to dominate inside the pitch have to keep possession of the ball with many passes (tiqui taca) and as a result of that deal with a great number of explosive actions to keep and regain ball possession. The purpose of this study was to review the physical needs of a team that wants to have increased ball possession to improve its chances of winning a game and to analyze how improved repeated sprint ability levels can support this tactical plan from a performance perspective.

**Materials and methods**

Standard systematic review methodology was modified and adopted for this review and electronic-searching tools were used to locate the papers needed. A total number of 35 studies were analyzed. Repeated Sprint Ability training strategies were evaluated based on the phase of a football season. We evaluated these strategies about their scientific substrate and their applicability in the training programs introduced by sports scientists as well as strength and conditioning coaches on the pitch.

**Results**

Our present systematic review revealed that these training methods are considered to be the most effective and popular ones today to support possession and all-around press game style.

**Conclusions**

More research is required to analyze the physical demands of the tiqui taca game style and how repeated sprint ability can be improved to assist all players corresponding to this kind of tactical plan.

## A99 Effects of volleyball sport and age factor on proprioceptive sense

### Emre Burak Gürlek^1^, Selcuk Akpinar^2^

#### ^1^Kozaklı Physical Therapy and Rehabilitation Hospital, Nevşehir, Türkiye; ^2^Faculty of Sport Science, Nevşehir Hacı Bektaş Veli University, Nevşehir, Türkiye

##### **Correspondence:** Selcuk Akpinar (selcukbos@gmail.com)

*BMC Proceedings 2024*, **18(11)**: A99

**Background**

In many different sports, the motor performance levels of athletes can vary significantly based on the preferences of athletes for a particular sport, depending on the skills they possess. This diversity indicates that the athletes’ skills and abilities can vary according to specific qualities. Therefore, evaluating athletes' performance levels and comparing them with non-athletes of the same age group is crucial for understanding and researching the effects of a sport on individuals.

**Materials and methods**

In this context, within the scope of this research, the motor performances of a group of female volleyball players aged 12-16 were compared with sedentary (non-athlete) individuals of the same age group during a proprioception-based reaching movement. This test aimed to measure participants' proprioceptive perception and motor performance levels. In this comparison, right-left arm performances (lateralization) were also examined. Motor performance measurements of 30 female volleyball players and 30 sedentary females were subjected to a comparative analysis considering both group and arms. Motor performance parameters such as accuracy, linearity, reaction time, and speed were statistically evaluated. A 2 groups (sedentary x volleyball) x 2 arms (right x left) mixed-model ANOVA was used as the statistical analysis method.

**Results**

The findings indicated that the volleyball players performed better than the sedentary group in terms of reaction time and linearity.

**Conclusions**

These results demonstrate a significant increase in motor performance parameters in volleyball players aged 12-16 compared to sedentary individuals. This was discussed in detail, taking into account the specific contents of sports and volleyball.

This study is derived from Emre Burak Gürlek's master's thesis.

## A100 Talent identification in youth football categories: A systematic review

### Bojan Rašković^1^, Dejan Javorac^1^, Rade Milić^1^, Marko Ubović^1^, Miloš Kojić^1^, Dušan Đorđević^2^, Slavko Molnar^1^, Borislav Obradović^1^

#### ^1^Faculty of Sport and Physical Education, University of Novi Sad, Novi Sad, Serbia; ^2^Faculty of Sport and Physical Education, University of Niš, Niš, Serbia

##### **Correspondence:** Bojan Rašković (bojan.raskovic@fsfvns.edu.rs)

*BMC Proceedings 2024*, **18(11)**: A100

**Background**

Many questions arise regarding this topic: when is the best period for identification, what are the indicators, how much influence does genetics have, does talent always become a top player, and how much does the work of experts affect the development of talented children? The paper aims to describe the practical and theoretical views of talent identification and look at talent development by synthesizing previous research.

**Materials and methods**

We developed a systematic review following PRISMA (2020) guidelines. We performed searches of the following electronic databases: Google Scholar, PubMed, and Web of Science. Keywords “talent identification”, “youth categories”, “football”, and “elite performance” were used for search in English, following the “PICOS” question model.

**Results**

Of the 388 studies initially identified, 14 met the inclusion criteria for the review. We analyzed the mentioned studies and created a discussion emphasizing five segments: 1) psychological factors 2) technical-tactical skills 3) anthropometric and physiological factors 4) biological age, and 5) environmental factors. The results show statistically significant differences in anthropometric characteristics, body mass, Intermittent Fitness Test, Illinois test, 20-meter sprint, and jump performance between elite and non-elite players, including different positions and levels. Several studies have highlighted the importance of biological and chronological age and technical and tactical skills for talent identification and football development.

**Conclusions**

Based on the reviewed papers, we can conclude that some identification tests can provide current information about the characteristics of players, but there is no single most important parameter for predicting talent. The proposal is to allow as many players as possible to be involved in the process, combining a scientific and practical approach with forming a base of top players who are identified at a younger age and eventually become top players.

## A101 Spinal mechanisms underlying the reduction of the H-reflex after short maximal isometric contractions

### Miloš Kalc^1^, Matej Kramberger^2^, Jakob Škarabot^3^, Aleš Holobar^2^

#### ^1^Science and Research Center Koper, Institute for Kinesiology Research, Koper, Slovenia; ^2^System Software Laboratory, Faculty of Electrical Engineering and Computer Science, University of Maribor, Maribor, Slovenia; ^3^School of Sport, Exercise and Health Sciences, Loughborough University, Loughborough, United Kingdom

##### **Correspondence:** Miloš Kalc (milos.kalc@zrs-kp.si)

*BMC Proceedings 2024*, **18(11)**: A101

**Background**

Maximal isometric contractions of short duration are known to enhance motor performance, a phenomenon referred to as post-activation potentiation (PAP). The increase in muscle force due to PAP is linked to various neural and contractile mechanisms. However, there is extensive research on the contractile elements of PAP, and the neural aspects, especially those involving spinal processes, are not as clearly understood. Therefore, this study focuses on exploring the impact of brief, maximal isometric contractions of the plantar flexors on the spinal elements that contribute to PAP.

**Materials and methods**

Fourteen males (21.9 ± 4.8 years) participated. They were instructed to either perform a 10-second maximal isometric contraction of their plantar flexors (PAP) or rest for the same duration (REST). These two conditions were alternated in a single visit, with a 20-minute washout period intervention. The Soleus H-reflex, D1 presynaptic inhibition, and Homonymous Ia facilitation (HF) were assessed in nine time periods after the contraction. The soleus muscle's EMG signals were collected using a 64-channel matrix electrode (GR08MM1305, OT Bioelettronica, Italy), and peak-to-peak amplitudes as well as single MUs firings from the electrically evoked responses were determined. The data were analyzed using repeated measures nested linear mixed effect models.

**Results**

Our study revealed that following a maximal voluntary contraction, the H-reflex amplitude significantly decreased (*p*<0.001), contrasting with an increase in heteronymous Ia facilitation (*p*<0.001) and unchanged D1 presynaptic inhibition. This finding suggests a complex interaction between neural and muscular responses post-contraction. The decrease in H-reflex might reflect a neural strategy to manage enhanced muscle performance or a concurrent occurrence of central fatigue and peripheral potentiation.

**Conclusions**

The results highlight a nuanced understanding of post-activation potentiation, suggesting intricate spinal and possibly corticospinal modulations, which necessitate further investigation to comprehend their roles in motor performance fully.

## A102 Digital football curriculum at Szeged-Csanád Grosics Akadémia

### Gábor Herczeg, Levente Szántai, Josip Lepeš, Ferenc Győri

#### Gál Ferenc University, Szeged, Hungary

##### **Correspondence:** Gábor Herczeg (herczeg.gabor@gfe.hu)

*BMC Proceedings 2024*, **18(11)**: A102

**Background**

The world of professional football is developing at an ever-faster pace. Consequently, young athletes wishing to become professional footballers at any level must acquire a skillset that is broader than ever before. How can they do that in a modern digital society where physical activity levels are decreasing? How can we (coaches, educators, and researchers) turn the tide and increase volume of deliberate practice as opposed to time spent on computers, smartphones, and other gadgets?

**Case report**

At Szeged-Csanád Grosics Akadémia we initiated a new project in 2022 aimed at collecting, organizing, and sharing our knowledge in a way that is sustainable, current, and engaging to our youth players and coaches. Our Digital Football Curriculum is on display at our Academy entrance and available to all our players, parents, and coaches through their smart devices. In this way, we can provide material for individual practice for hundreds of players which we believe will hugely contribute to their football development. In our case report we would like to share display three main areas of interest:

1. How to structure a Digital Football Curriculum? 2. How to utilize a Digital Football Curriculum in coach education and staff development? 3. How to apply a Digital Football Curriculum in individual player development?

**Conclusion**

As an educational institute we firmly believe that sharing good practice is key in developing young athletes. Adopting the concept of the Digital Curriculum should help sports clubs to optimize their development process and maximize the efficiency of their time spent on the pitch / court / field, etc. As the research unit of Gál Ferenc University we believe that this concept can be a firm starting point for empirical research.

## A103 Prevalence of chronic noncommunicable diseases in relation to quality of life and physical activity in Colombian university students.

### Brahian Steven Castrillón Rendon^1^, Carlos Federico Ayala Zuluaga^2^, Maria Valentina Suarez Leon^1^, Javier Eduardo Castrillón Escudero^1^

#### ^1^National University of Colombia, Manizales, Colombia; ^2^Caldas University, Manizales, Colombia

##### **Correspondence:** Brahian Steven Castrillón Rendon (bscastrillonr@unal.edu.co)

*BMC Proceedings 2024*, **18(11)**: A103

**Background**

The university student's lifestyle is characterized by a large number of hours of daily sedentary behaviors, high academic stress, irregular sleep habits, unhealthy habits and lifestyles, tobacco and alcohol consumption and high-risk sexual practices. All these behaviors constitute risk factors for developing chronic noncommunicable diseases, which are responsible for more than one-third of the world's annual deaths. The objective of this study is to determine the prevalence of chronic noncommunicable diseases in relation to quality of life and physical activity in university students.

**Materials and methods**

The sample consisted of 352 students (210 men and 142 women), a quantitative, observational, prospective, descriptive and relational cross-sectional study; a sample of students from the National University of Colombia will be evaluated. Information was collected using (IPAQ and WHOQOL BREF), and statistical analysis was performed with SPSS v.29. The study has been approved by the Bioethics Committee of the Universidad Nacional.

**Results**

When comparing the quality of life using the WHOQOL-BREF, university students showed a significantly higher score than students classified as inactive, both in global QoL (*p*=0.002; d=0.67) and in health QoL (*p*=0.003; d=0.63). When comparing QoL between men and women, there were no significant differences in all the dimensions evaluated (*p*<0.05).

**Conclusions**

University students with high or moderate levels of physical activity show a significant improvement in their general quality of life and specific health-related aspects compared to those with low levels of physical activity. The implementation of actions aimed at promoting physical activity in universities important.

## A104 Adherence and effects of the neuromuscular training for physical activity related injuries in adolescent basketball players

### Boštjan Šimunič^1^, Katarina Puš^1,2,3^, Kaja Teraž^1,2^, Miloš Kalc^1^, Manca Peskar^1,4^

#### ^1^Science and Research Center Koper, Institute for Kinesiology Research, Koper, Slovenia; ^2^Faculty of Sport, University of Ljubljana, Ljubljana, Slovenia; ^3^Department of Health Sciences, Alma Mater Europaea – ECM, Maribor, Slovenia; ^4^Biological Psychology and Neuroergonomics, Technische Universität Berlin, Berlin, Germany

##### **Correspondence:** Boštjan Šimunič (bostjan.simunic@zrs-kp.si)

*BMC Proceedings 2024*, **18(11)**: A104

**Background**

Neuromuscular training (NMT) is widely recognized as an effective physical activity and sport-related injuries (PARI) preventive program. NMT programs are typically coach-led programs that are designed to improve balance, strength, agility, coordination, and movement control. The aim was to evaluate the effects, adherence, maintenance, and acceptance of 3-month NMT with 3-month follow-up in adolescent basketball teams.

**Materials and methods**

Twenty adolescent male basketball teams (275 players, aged 12-15 years) were divided into control (CG, *N*=146) and experimental groups (EG; *N*=129). For the next three months, CG maintained their regular basketball training, while EG performed a 15-minute NMT instead of a classical warm-up. After the 3-month intervention period, all teams were informed about the effectiveness of NMT intervention and were left to perform NMT intervention as they like, until the end of the next 3-month follow-up period. Pre, and post 3-month intervention and post-follow-up periods anthropometry, morphology (bioimpedance), skeletal muscle Tensiomyography (TMG), NMT adherence, acceptance, and maintenance (interviews) and cognition (Trail-making test, CORSI block tapping task, simple and choice reaction time) were assessed.

**Results**

The prevalence of PARI was lower in EG than in CG in the intervention period (10% vs. 14%) and in the follow-up period (1% vs 9%). TMG delay time declined (VL) or stayed the same in EG while increasing in the CG. Similarly, the contraction time remained the same in EG while increasing in CG. During the intervention period, the EG achieved higher adherence to NMT, 91% and 23% during training and match sessions, respectively. Meanwhile, CG achieved 41% during the follow-up period in training sessions and 0 % during match sessions. In the cognitive domain the two groups did not differ.

**Conclusions**

We have provided evidence of NMT's effectiveness as an intervention for PARI. We also reported high adherence to NMT of PARI prevention programs.

## A105 Prevalence of sarcopenia in Slovenia

### Katarina Puš^1,2,3^, Miloš Kalc^1^, Peter Kokol^4^, Helena Blažun Vošner^5^, Jernej Završnik^5^, Boštjan Šimunič^1^

#### ^1^Institute for Kinesiology Research, Science and Research Centre Koper, Koper, Slovenia; ^2^Department of Health Sciences, Alma Mater Europaea – ECM, Maribor, Slovenia; ^3^Faculty of Sport, University of Ljubljana, Ljubljana, Slovenia; ^4^Faculty of Electrical Engineering and Computer Science, University of Maribor, Maribor, Slovenia; ^5^Community Healthcare Center Dr. Adolf Drolc Maribor, Maribor, Slovenia

##### **Correspondence:** Katarina Puš (katarina.pus@zrs-kp.si)

*BMC Proceedings 2024*, **18(11)**: A105

**Background**

Sarcopenia, a musculoskeletal disease, affects between 10 and 27% of the aged population, depending on the used classification protocol. Despite the burden of the disease, there is no unified classification protocol for sarcopenia to be used in clinical practice. There are different protocols in use; for European population most used are two (2010 and 2019) from the European Working Group on Sarcopenia in Older People (EWGSOP). However, there are modifications in the preliminary tests which were taken into account in this study. Our study aims to present agreements between five sarcopenia classification protocols in Slovenian older adults.

**Materials and methods**

Our sample included 658 participants (>60 years, 70% female) from Slovenia. Of these, data from 611 had complete parameters for classification by all five protocols. Data was gathered on body height, body mass, average grip strength, 5 sit-to-stand test performance, gait speed, timed up-and-go test, and muscle mass using electrical bioimpedance. The prevalence of sarcopenia was calculated from five different classification protocols (SDOC, EWGSOP, EWGSOP2, EWGSOP2 with SarCalF and EWGSOP2 without Sarc-F).

**Results**

The prevalence of sarcopenia was 15.2 %, 11.5 %, 3.03 %, 3.34 % and 6.5 % for SDOC, EWGSOP, EWGSOP2, EWGSOP2 with SarCALF and EWGSOP2 without Sarc-F, respectively. Overall agreement between protocols was low (Cohenκbetween .200 and .696).

**Conclusions**

The wide range of reported prevalence between protocols sheds light on the lack of universal consensus in defining and diagnosing sarcopenia. The discrepancy in prevalence could be attributed to variations in measurement methods employed by each protocol, making it more difficult to appropriately classify sarcopenic individuals. As EWGSOP2 has been the most used protocol in recent years, we can say that it largely underestimates the prevalence of sarcopenia. Achieving consensus in the diagnostic guidelines would enhance the accuracy and reliability of sarcopenia diagnoses, ensuring improved patient care.

## A106 Does foot alignment affect balance of elementary school gymnasts?

### Tatjana Trošt Bobić, Sven Lukić, Lidija Petrinović, Lara Juriša

#### Faculty of Kinesiology, University of Zagreb, Zagreb, Croatia

##### **Correspondence:** Tatjana Trošt Bobić (tatjana.trost.bobic@kif.unizg.hr)

*BMC Proceedings 2024*, **18(11)**: A106

**Background**

Due to sedentary lifestyle, our foot alignments are changing. Poor foot condition could negatively affect the ability of beginner gymnasts to maintain balance and impede their mastery of specific sports elements, although this has not been investigated yet. This research aimed to determine the relationship between foot alignment and balance in schoolchildren training gymnastics.

**Materials and methods**

The research included 18 female 8±1.4 years old gymnasts, with 1 year of gymnastics training. Their body mass, body height, foot alignment and balance were measured. The dominant leg was defined as the leg subjects preferred to maintain balance on. Foot alignment was assessed with a digital foot scanner, and Clarke’s angle was determined on the obtained plantogram. Balance was measured using the Y balance test and the 40-second unilateral stance on a moving platform test. The total distances reached in three directions (in cm) from the Y test were taken for further analysis. A custom-made platform, equipped with the Gyko Microgate electronic device, was used for the unilateral stance test. Data were processed in the Statistica software (version: 14.0.0.15) through descriptive statistics and correlation analysis.

**Results**

A strong correlation has been established between foot alignment and the results in the Y balance test (*r*=0.65) on the dominant leg. Additionally, a moderate correlation has been identified between foot alignment and both the ellipse area (*r*=0,52) and the antero-posterior average distance covered by the platform (*r*=0.52) for the non-dominant leg.

**Conclusions**

There is a certain degree of relationship between foot alignment and balance in young gymnastic beginners, however its manifestation varies between the leg dominance. Further studies are necessary; however these findings may help PE teachers and trainers in optimizing gymnasts’ balance, which is a basic motor ability needed to master specific gymnastics elements.

## A107 Does running performance differ on domestic and international games in TSC football team?

### Szabolcs Halasi^1,2^, Nándor Angyal^3^, Anita Štajer^1^, István Thékes^2^, Ferenc Győri^2^, Dušan Stupar^4^, Tihomir Dugandžija^5^

#### ^1^Hungarian Language Teacher Training Faculty, University of Novi Sad, Subotica, Serbia; ^2^Research Institute, Gál Ferenc University, Szeged, Hungary; ^3^Faculty of Sport and Physical Education, University of Novi Sad, Novi Sad, Serbia; ^4^Faculty of Sport and Psychology-TIMS, Educons University, Novi Sad, Serbia; ^5^Faculty of Medicine, University of Novi Sad, Novi Sad, Serbia

##### **Correspondence:** Szabolcs Halasi (halasi.sabolc@gmail.com)

*BMC Proceedings 2024*, **18(11)**: A107

**Background**

GPS technology is now increasingly used in major football teams worldwide and in Serbia, too. Data provide sports scientists, coaches, and trainers with comprehensive and real-time analysis of on-field player performance during and after competition or training. The aim of the study was to investigate the difference in TSC football team performance between domestic and international matches.

**Materials and methods**

We analyzed 14 officials domestic (Mozzart Bet Super Liga Srbija) matches and 8 international (UEFA Champions League 3rd qualification round and UEFA Europe League) matches of the 2023/24 season. The distance- and accelerometry-based measures were recorded in real-time during the matches using a wearable 10-Hz GPS integrated with a 400-Hz Tri-Axial accelerometer and 10-Hz Tri-Axial magnetometer (Playertek, Catapult Innovations, Australia).

**Results**

The average distance by domestic games was 10582.9±350.9 m, by international games 10657.2±362.2 m, in moderate 1256.5±94.8 vs. 1303.75±122.77 m, in high 359.5±66.44 m vs. 323.2±64.2 m, and sprint 131±41.27 vs. 94.1±39.1 m intensity zones. Statistically no significant differences between running performances.

**Conclusions**

The results of this paper showed that some players have personal best results in international games, but the team average in total, moderate, high intensity and sprint runs is almost same in the both competitions. In autumn 2023/24 season TSC had very intensive calendar, and the results show that running structure is enough for the 3rd place in the domestic league, but in the international competition it was enough only for 1 draw game. In football not only the running performance matters, but if we compare European top leagues with the domestic league, it is obvious that the structure of the running performance is below.

## A108 Factors related to aggression and interpersonal violence manifestation in youth sport

### Brigita Banjac^1^, Radenko Matić^1^, Željka Bojanić^2^, Sandra Radenović^3^, Ivana Milovanović^1^

#### ^1^Faculty of Sport and Physical Education, University of Novi Sad, Novi Sad, Serbia; ^2^Faculty of Legal and Business Study, Dr. Lazar Vrkatić, Novi Sad, Serbia; ^3^Faculty of Sport and Physical Education, University of Belgrade, Belgrade, Serbia

##### **Correspondence:** Brigita Banjac (brigita.banjac@fsfvns.edu.rs)

*BMC Proceedings 2024*, **18(11)**: A108

**Background**

Recently, many athletes have spoken up about their experience with aggressive behaviors. Awareness of this social issue has gained, and much research has highlighted that youth are exposed. Therefore, this study aimed to identify factors related to aggression and interpersonal violence (IV).

**Materials and methods**

This study included a cross-sectional design with a sample of 2091 athletes (39% male, 61% female) from the territory of Vojvodina in Serbia. The data were collected through a questionnaire between 2019-2020. The sample characteristics included athletes’ age (younger 11-14/older 15-18 years), training history (1-5 years/more), sports category (individual/collective), and type (contact/non-contact). For testing the differences, the Chi-squared test and the Mann-Whitney U test were applied with a significance level of 0.05.

**Results**

The results revealed that factors such as age (χ2=28.41), category (χ2=48.13), and type (χ2=65.71) of sport are related to aggression and IV presence. Specifically, being older practicing collective or contact sports are associated with a higher percentage of these destructive behaviors’ manifestation. Furthermore, the physical type of violence is more common for older athletes, for members of collective and contact sports. The psychological type is dominant for older athletes, for those with five or more years of training history, and for those included in a collective or non-contact sport.

**Conclusions**

Although sport has many benefits, it is not immune to aggression and IV. More profound knowledge is necessary about their way of manifestation. The initial step is to define some variables that are associated with them.

## A109 The relationship of the body composition and the motor abilities of female soccer players

### Stefan Stojanović^1^, Bojan Jorgić^1^, Ismail Ilbak^2^, Doroteja Rančić^1^

#### ^1^Faculty of Sport and Physical Education, University of Niš, Niš, Serbia; ^2^Institute of Health Sciences, Department of Physical Education and Sports, İnönü University, Malatya, Türkiye

##### **Correspondence:** Stefan Stojanović (stefan.stojanovicfsfv@gmail.com)

*BMC Proceedings 2024*, **18(11)**: A109

**Background**

For top success in soccer, it is necessary to have complete and mutual preparation of top technical, tactical, and physical domains in the form of well-developed motor skills. The most dominant abilities essential for success are agility, explosive power, speed, coordination, and cardiorespiratory endurance. Many studies have confirmed the association between parameters of body composition and the motor skills of female soccer players. Accordingly, the aim of this study was to determine the relationship between body composition parameters and the motor skills of female soccer players.

**Materials and methods**

The research included thirty female soccer players (aged 15.7±1.1 years), who trained soccer for 4.1±1.9 years. Body composition was assessed with bioelectrical impedance (Omron BF511), and body height (BH) using the Martin anthropometer. Body mass index (BMI), was calculated as weight divided by stature squared (kg/m2) Explosive power was determined by squat jump (SJ), countermovement jump (CMJ), and countermovement jump with arm swing (CMJarm). Speed was assessed by sprint tests at 5, 10, 20m. Agility was assessed by: T-test, slalom without ball and slalom with ball.

**Results**

Body height is significantly related to explosive power (*r*= -.676), while BW is significantly related to speed (*r*= .551). Body mass index was significantly correlated with explosive power (*r*= -.546). BF% showed a significant correlation with speed and explosive power, while percent of muscle mass showed a significant correlation with explosive power (*r*= .512; .311, respectively). In the end, no significant correlation was found between body composition parameters and the agility of soccer players.

**Conclusions**

Based on the results of this study, it was proven that body composition parameters are related to motor abilities of female soccer players.

## A110 Effects of supplementation on motor abilities of basketball players

### Tamara Ilić^1^, Stefan Stojanović^1^, Doroteja Rančić^1^, Ismail Ilbak^2^

#### ^1^Faculty of Sport and Physical Education, University of Niš, Niš, Serbia; ^2^Institute of Health Sciences, Department of Physical Education and Sports, İnönü University, Malatya, Türkiye

##### **Correspondence:** Tamara Ilić (tamarailic.fsfv@gmail.com)

*BMC Proceedings 2024*, **18(11)**: A110

**Background**

Although the basic rules of basketball are practically the same to this day, the characteristics of the game itself are changing very quickly and are defined as more complicated. In such conditions, success in basketball does not only require technical, tactical and psychological skills, but also depends on motor abilities and nutrition. Sports supplementation is any dietary manipulation in an attempt to improve sports performance, and it has become very popular among athletes. In this regard, the goal of this study was to gain insight into the effects of supplementation on the motor abilities of basketball players through a systematic review of previous literature.

**Materials and methods**

The sample of respondents consisted of a total of 224 basketball players. The following index databases were used to collect adequate literature: Google Scholar, PubMed, Web of Science. The criteria for the selection of the studies were as follows: studies investigating the effects of supplementation on the motor abilities of basketball players, the existence of one or more experimental groups, or one/more experimental and control groups; existence of data on initial and final measurement, as well as tested parameters and existence of data on used supplements. Fifteen original scientific studies were included in the final analysis.

**Results**

After analyzing the results, we observed that there are numerous supplements that can improve the motor skills of basketball players, such as: caffeine, whey/casein proteins, drinks with electrolytes, glutamine, b-alanine. While antioxidant supplements or beetroot juice were ineffective in improving motor skills.

**Conclusions**

It can be concluded that a variety of substances might enhance the motor abilities of basketball players. However, beetroot juice or antioxidant supplements did not help with motor abilities improvement. In the end, it's critical to emphasize that supplements cannot take the place of food as a source of essential nutrients.

## A111 The influence of music on the performance of general preparatory exercises in preschool children

### Gordana Furjan-Mandić, Ana Mekovec, Josipa Radaš

#### Faculty of Kinesiology, University of Zagreb, Zagreb, Croatia

##### **Correspondence:** Gordana Furjan-Mandić (gfurjan@kif.hr)

*BMC Proceedings 2024*, **18(11)**: A111

**Background**

For healthy psychophysical growth and development, a preschool aged child needs to meet the basic human needs, and one of the most important of them is movement. Proper physical activity of children affects their health status. Physically active children have stronger muscles and bones, a slimmer body because exercise controls the amount of fat tissue, they are less likely to become obese and suffer from diabetes and have lower blood pressure and cholesterol levels.

**Materials and methods**

An experimental procedure was used to analyze the differences between the regularity of performing general preparatory exercises, discipline, and motivation. In the first class, they performed ten preparatory exercises without music. In the next class, they performed the same exercises to a given rhythm with applause, and in the third class they performed the exercises with music. The sample of subjects consisted of 21 preschool children ages 3 to 6. The differences in performance between the three ways of performing movement structures were determined by multivariate analysis (MANOVA), and a post-hoc test was used to determine the difference between practicing without music, at a given rhythm and with music.

**Results**

The results obtained based on the multivariate test (MANOVA) showed that there was no significant difference between the groups (F=1.43; df=2; *p*=0.222).

**Conclusions**

Contrary to the results, but based on practical experience it can be concluded that music helps in the training of preschool children to a great extent. It improves the working atmosphere and gives children the freedom to move. Better discipline during exercise with music was also observed.

## A112 Effects of blood flow restriction training and traditional resistance training on 1rm squat and vertical jump performance following a 4-week intervention in well-trained males

### Roan Kotze^1,2^, Željko Banićević^2^, Ivana Banićević^2^

#### ^1^OMNI Performance, Dubai, United Arab Emirates; ^2^HERC- Health, Exercise & Research Center, Dubai, United Arab Emirates

##### **Correspondence:** Ivana Banićević (ivana@hercme.com)

*BMC Proceedings 2024*, **18(11)**: A112

**Background**

The primary objective was to investigate the impact of lower extremities bilateral cuff pressure (180mmHg) and resistance training on 1RM back squat and vertical jump performance in individuals with ≥ 3 years of resistance training experience following a 4-week intervention.

**Materials and methods**

Fifteen well-trained males (mean ± SD, age:34.3 ± 4.34 years, height:180.06 ± 3.91cm, body mass:82.67 ± 7.48kg, back squat 1RM:129.69 ± 25.99kg, CMJ height:37.94 ± 7.23cm) with ≥ 3 years resistance training were recruited. A 4-week intervention with two sessions per week included a blood flow restriction (BFR) training group and a control group (CON). Pre and post-testing assessed CMJ and 1RM squat performance.

**Results**

A two-way mixed ANOVA showed no statistically significant interaction between the intervention and time on 1RM squat (F(1,13) = 4.050; p = .065; p2 = .408) and CMJ (F(1,12) = 0.132; *p* = .723. p2 = .011). The main effects of time showed a statistically significant increase from pre to post-intervention in mean 1RM squat (F(1,13) = 8.970; *p* = .010; p2 = .408) and a statistically significant decrease from pre to post-intervention in mean CMJ (F(1,12) = 33.786; *p* = .000; p2 = .738). There was no statistically significant difference between intervention groups in mean 1RM squat (F(1,13) = .016; *p* = .901; p2 = .001) and mean CMJ (F(1,12) = 1.248; *p* = .286; p2 = .094).

**Conclusions**

Both BFR training and traditional resistance training improved 1RM squat performance over time. CMJ performance decreased significantly for both groups. No differences were observed between groups for 1RM squat and CMJ, indicating that BFR training combined with resistance training enhances squat strength, but not jump performance.

## A113 The influence of a 12-week circular exercise program on the motor status of a previously inactive female population

### Mateja Očić, Vedran Dukarić, Damir Knjaz

#### Laboratory for Sports Games, Faculty of Kinesiology, University of Zagreb, Zagreb, Croatia

##### **Correspondence:** Mateja Očić (mateja.ocic@kif.unizg.hr)

*BMC Proceedings 2024*, **18(11)**: A113

**Background**

The decreased level of physical activity of nowadays generation is mainly caused by hectic lifestyle and sedentary habits. Most of research determined higher prevalence of insufficient physical activity in female population rather than male. To maintain the functionality of the entire locomotor system, it is necessary to continuously participate in activities aimed at developing the strength of the whole body, with the focus of activating all major muscle groups. According to ACSM recommendations, to develop muscle strength, it is necessary to participate 2-3 times a week in training with external load.

**Materials and methods**

The aim of this study was to determine whether continuous and programmed strength-oriented physical activity in duration of 12 weeks has positive impact on changes in motor status of previously physically inactive middle-aged women (*n* = 51). Specific circuit training program was carried out 2 times a week with the emphasis on the development of strength and improving the overall core stability. Exercise intensity progressively increased, and the exercises were performed from simpler to more complex.

**Results**

Results of repeated measures ANOVA indicate statistically significant differences (F=15.85, *p*<0.001) in all of the observed variables (lunges, sit-ups and push-ups), between 3 time points (initial, after 6 and after 12 weeks). At the final test, significantly better results were achieved compared to the first two measurement points. Based on the obtained results, it is possible to conclude that the respondents achieved significant changes in motor status after 12 weeks of programmed physical exercise.

**Conclusions**

Application of content with external load activates large muscle groups, which affects the increase in core stability and the increase in strength of the lower and upper extremities. Furthermore, results of this study confirm the importance of physical activity in female population, especially because of the well-known beneficial effect of exercise on bone density and muscular fitness.

## A114 Differences in activity level and HR zones of indoor and outdoor training programs

### Vedran Dukarić, Mateja Očić, Damir Knjaz

#### Laboratory for Sports Games, Faculty of Kinesiology, University of Zagreb, Zagreb, Croatia

##### **Correspondence:** Vedran Dukarić (vedran.dukaric@kif.hr)

*BMC Proceedings 2024*, **18(11)**: A114

**Background**

Due to reduction of physical activity, people are starting to have problems with physical and mental health which consequently affect social status of individuals. In order to prevent the consequences of overall inactivity, cardio-type exercises are recommended for which multiple benefits have been proven. The aim of this research was to determine differences between training loads of participants (*N*=36) included in indoor (ergometers) and outdoor (Nordic walking – NW) training programs.

**Materials and methods**

Each participant attended 24 trainings in a period of 3 months (2 trainings per week). Initial measurement was held to determine maximum heart rate (HRmax) and basic anthropological status. At the start of each training, participants were given a smart watch to track duration and HR parameters. Multivariate analysis of variance (MANOVA) was used to determine differences between groups.

**Results**

Results showed significant differences between groups (F=16.13, *P*=0.00). Furthermore, univariate analysis of variance (ANOVA) determined differences in duration of training (F=17.75, *p*=0.00) and time spent in zone 3, 4 and 5 (*p*=0.00). The Nordic walking group spent most time in zone 2 (60 - 70% of HRmax) compared to the indoor group that had most activity in zone 3 (70 - 80% of HRmax). Indoor training group had more active time during one training (66 min vs. 55 min) and spent more time in higher training zones (3, 4 and 5).

**Conclusions**

It can be concluded that indoor training participants were much longer in high intensity and that these two training methods differently effect on physiological parameters of individuals. However, it is recommended to choose some form of cardio exercises in order to have a long-term effect on improving the health status of individuals.

## A115 Differences in eating routines among medical and non-medical faculties in Novi Sad

### Branislava Teofilović^1^, Nevena Grujić-Letić^1^, Emilia Gligorić^1^, Aleksandar Takači^3^, Daniela Kenjerić^2^

#### ^1^Faculty of Medicine, University of Novi Sad, Novi Sad, Serbia; ^2^Faculty of Food Technology, “Josip Juraj Strossmayer” University of Osijek, Osijek, Croatia; ^3^Faculty of Technology, University of Novi Sad, Novi Sad, Serbia

##### **Correspondence:** Branislava Teofilović (branislava.teofilovic@mf.uns.ac.rs)

*BMC Proceedings 2024*, **18(11)**: A115

https://scindeks.ceon.rs/Article.aspx?artid=0018-68722202015T&lang=en

## A116 Implications of structured physical activity and sports programs in reducing inmates’ aggression - Systematic review

### Marian Cosmin Tomescu

#### Doctoral School of Psychology, Faculty of Physical Education and Sport, West University of Timisoara, Timisoara, Romania

##### **Correspondence:** Marian Cosmin Tomescu (cosmin.tomescu@yahoo.com)

*BMC Proceedings 2024*, **18(11)**: A116

**Background**

Structured, purposeful physical activity has effects on personality dimensions and behavior of offenders. This type of activity can contribute significantly to reducing maladaptive behavior. The European Prison Rules recommend that all prisoners should be given the opportunity to participate regularly in organized sport and physical education. Previous research has shown that by channeling energy into physical education, these can be reduced, thus contributing to the effective social reintegration of prisoners.

**Materials and methods**

The aim of this systematic review is to highlight the importance of physical activity on the development of inmates' abilities to control their aggression, thereby contributing to increased chances of social reintegration and prevention of recidivism by shaping self-esteem and locus of control. After browsing the existing literature on scientific platforms, 10 relevant articles were included in the study and analyzed. The selection of studies was performed through a 3-step process: title evaluation, abstract evaluation and full text evaluation. The selected studies were conducted on adult and young male and female populations, regardless of ethnicity and published between 2001-2021.

**Results**

All participants in the studies reviewed were inmates who participated in structured physical education and sport programs in the prison environment. The exercise protocols varied, so there were training programs focused on resistance and strength training; and their effect was correlated with reduced aggression, increased self-esteem, and other aspects specific to prison life.

**Conclusions**

The redirection of aggressive impulses, together with the improvement of self-esteem and locus of control, can be achieved through training and practice of physical skills. Thus, it contributes significantly to the prevention and reduction of harm associated with aggressive behavior in detention.

## A117 Analysis of the six-minute walk test (6MWT) as an indicator of the circulatory system in older female

### Ferenc Ihász, Angéla Somogyi

#### Széchenyi István University, Győr, Hungary

##### **Correspondence:** Angéla Somogyi (somogyi.angela@sze.hu)

*BMC Proceedings 2024*, **18(11)**: A117

**Background**

Biological ageing is associated with a loss of muscle mass, strength and cardiorespiratory fitness, which leads to decline in the ability to engage in everyday activities. The aim of this study was to investigate the relationship between self-reported health-related quality of life and current circulatory status (specifically based on the results of a 6-minute walking test) among community-dwelling older women.

**Materials and methods**

The cross-sectional study included 149 female, 79.63 (SD = 8.68, min=51.91, max=94.42) from four nursing homes in the West-Hungary region. Three groups were created: under 75 years: 24.8% (*n*=37); 75-85 years: 49.7% (*n*=74); 85 years and over: 25.5% (*n*=38). Aerobic fitness was evaluated using the six-minute walk test (6MWT), which is a physical performance test widely used in research, especially to obtain valid measures of submaximal aerobic endurance in older adults (Rikli RE, Jones CJ. 1998). Subjective health-related quality of life was assessed using the perception of health on the HRQoL SF-36 (Staquet MJ, et al. 1998).

**Results**

We have found differences between age groups in the 6MWT walking test. The highest score was achieved by those under 75 years (267.28±146.75) significantly lower by people over 85 years (191.23±102.74); F=3.279; *p*<0.036 and between 75-85 years (225.57±124.27). The distance accomplished in the 6MWT walking test was -193.47±141.23 m shorter than the expected distance. Between the age groups, the SF-36 mental item scored highest among over 85-year-olds, significantly lower among the 75–85-year-olds, and under 75-year-olds scored in between.

**Conclusions**

Declines in aerobic fitness were strongly associated with quality of mental health during the ageing process.

## A118 Effect of race days' locomotor stress on the circulatory system in elite rowers

### Zsolt Bálint Katona^1^, Zoltán Alföldi^1^, Tamás Gyömörei^1^, Angéla Somogyi^2^, Ferenc Ihász^2^

#### ^1^Recreation and Sports Centre, Széchenyi István University, Győr, Hungary; ^2^Faculty of Health and Sport Science, Széchenyi István University, Győr, Hungary

##### **Correspondence:** Zsolt Bálint Katona (zsbkatona@googlemail.com)

*BMC Proceedings 2024*, **18(11)**: A118

**Background**

Research has shown that oxygen is the main physiological determinant of critical power (Walsh ML., 2000), but during exercise many different tissue systems need to increase their metabolism and each tissue system, such as the heart, respiratory and leg muscles, has its own critical power. Fatigue is defined as a decrease in maximal voluntary effort. The main objective of this study is to determine the effects of locomotor stress on the circulatory system during race days in elite rowers.

**Materials and methods**

Two groups were created to reflect the frequencies of the three-day competition events. Data were recorded using a “Polar Team Pro” system including accelerometer, a gyroscope and GPS.

Explosiveness tests were carried out on the three days, before and after each heat, intermediate races and finals. A PJS-4P60S power meter “plateau” (Gajewski, J. et al., 2018) with 400 Hz sampling frequency (“JBA” Zb. Staniak, Poland) was used to measure the power output of the cast (“JBA” Zb. Staniak, Poland). "Daily wellness questionnaire" - fatigue, stress, sleep, muscle was completed online by the participants. part (Gastin, P.B., et al., 2013).

**Results**

No differences were found in the time averages spent in the pulse intensity zones of the first, second and third day with respect to the pulse patterns in either the first or second group. The pattern of the explosiveness test on the force plateau coincides with fatigue. In terms of differences, the medians of the heats, semi-finals and finals patterns during the days and race days are in the negative range. For the Wellness questionnaire, the 3 days muscle fatigue pattern is significantly different, the value of day 3rd is the smallest.

**Conclusions**

Peripheral fatigue could not be confirmed by pulse results, but could be confirmed by the explosiveness test and the muscular questions of the wellness questionnaire.

## A119 The relationship between primitive reflex profile and development of vestibular maturity in early school years

### Erzsébet Stephens-Sarlós

#### Faculty of Health and Sport Science, Széchenyi István University, Győr, Hungary

##### **Correspondence:** Erzsébet Stephens-Sarlós (stephens-sarlos.erzsebet@sze.hu)

*BMC Proceedings 2024*, **18(11)**: A119

**Background**

Previous research indicates that vestibular perception is related to muscle tone regulation. Muscle tone influences auditory and visual perception. Research findings suggest that functioning of primitive reflexes affects the maturation and condition of the vestibular organ. The goal of the study is to examine whether primitive reflex profile can be influenced by appropriate exercises and whether inhibition of persistent primitive reflexes affects vestibular functions.

**Materials and methods**

The sample consisted of 506 children aged 5–8 years (237 boys and 269 girls). In kindergartens and schools, 443 participants performed reflex inhibitory exercises 3–5 times a week for 7 months. A control group of 63 individuals attended only regular PE lessons. Input and output results were compared using the Mann-Whitney test, and effect sizes were calculated. Input primitive reflex profile and vestibular maturity: not significant. Effect size: primitive reflex profile: 0.109; vestibular maturity: 0.052. Output: primitive reflex profile: *p* <0.001; vestibular maturity: *p* <0.001. Effect size: primitive reflex profile: 0.572; vestibular maturity: 0.306.

**Results**

The results obtained with appropriate non-parametric measurements show that children participating in the intervention achieved statistically significantly higher scores in all tested variables of primitive reflex profile and vestibular maturity compared to the control group. The children in the intervention group showed significant improvement in both primitive reflex profile and vestibular maturity: *p* <0.001; effect size: primitive reflex profile: 0.714; effect size: vestibular maturity: 0.664, while there was no significant improvement in the control group: effect size primitive reflex profile: 0.01; Vestibular maturity: 0.06.

**Conclusions**

We found a strong, significant correlation between the inhibition of primitive reflexes and vestibular maturity: *r*=0.000; rho (ρ)=0.000. It is worth considering incorporating these exercises into physical education for 5–8-year-old children, since vestibular maturity plays a significant role in muscle tone and auditory and visual perception.

## A120 Relationship between physiological and psychological characteristics in elite rowers

### Zoltán Alföldi^1^, Ivan Petrov^2^, Imre Soos^3^, Angela Somogyi^1^, Laszlo Suszter^4^, Emese Santa^5^, Tamas Gyomorei^6^, Erzsebet Sarlos^1^, Zsolt Katona^1^, Ferenc Ihasz^1^

#### ^1^Faculty of Health and Sport Science, Széchenyi István University, Győr, Hungary; ^2^Faculty of Health Sciences, Doctoral School of Health Sciences, University of Pécs, Pécs, Hungary; ^3^Faculty of Health Sciences, Institute of Physiotherapy and Sport Science, University of Pécs, Pécs, Hungary; ^4^Sports and Health Sciences Research Group, Eszterházy Károly Chatolic University, Eger, Hungary; ^5^Faculty of Health Sciences, Institute of Emergency Care, Pedagogy of Health and Nursing Sciences, University of Pécs, Pécs, Hungary; ^6^Széchenyi István University Győr, Sport and Recretional Centre, Győr, Hungary

##### **Correspondence:** Zoltán Alföldi (alfoldi.zoltan@sze.hu)

*BMC Proceedings 2024*, **18(11)**: A120

**Background**

The psychobiological model predicts that any psychological or physiological factor that increases potential motivation or reduces perception of effort will improve endurance performance. The aim of the study was to examine the associations between somatic and mental distress and self-efficacy on stress management, concentration and performance, in our case 2000 m rowing ergometer run time, estimated aerobic capacity by gender and age.

**Materials and methods**

Fourteen rowers (6 boys, 8 girls), aged 15-18 years (M=16.1, SD=1.3), with a training age of 1-7 years (M=4.2, SD=1.9) participated in the study. The participants were among the top of the national rowing sport and among the international midfield. Anthropometric (Body height (BH) cm, body mass (BM) kg measured and BMI calculated, physiological (2000 m test (sec.) completed on a certified rowing ergometer (Concept 2 D-model) characteristics were performed in the middle of the 2020-racing season. The estimated relative aerobic capacity (ErVO2max) was calculated by using the formula of McArdle et al. (2006). Competitive State Anxiety Inventory-2 (CSAI-2) was used before the 2000 m rowing ergometer test, and Sport Competition Anxiety Test (SCAT) and Athletic Coping Skills Inventory (ACSI-28) were used after the test. Linear regression analyses were employed to constitute two different prediction models.

**Results**

The main influencing factors on the result of the 2000m ergometer performance are age, gender and ErVO2max, which explain nearly 80% of the variability in results of the rowing tests. Dependent variable: 'Time 2000 m (sec.)', Independent variables: ‘age, SCAT, ACSI-28, CSAI-2. Some independent variables, measuring very similar constructs, should be omitted from the model. Based on this consideration: Individual factors of CSAI are unsurprisingly correlated: cognitive anxiety-somatic anxiety: 0.805; cognitive anxiety-self-consciousness: -0.821; somatic anxiety-self-consciousness: -0.877.

**Conclusions**

If physiological performance characteristics show lower physical fitness, the psychological profile does not contribute to better performance.

## A121 Body height of elite volleyball players

### Duško Cvijović, Lana Bajić, Sunčica Poček

#### Faculty of Sport and Physical Education, University of Novi Sad, Novi Sad, Serbia

##### **Correspondence:** Duško Cvijović (dusko.cvijovic@fsfvns.edu.rs)

*BMC Proceedings 2024*, **18(11)**: A121

**Background**

Volleyball is a sport played by two teams on a playing court divided by a net. The object of the game is to send the ball over the net in order to ground it on the opponent’s court, and to prevent the same effort by the opponent. It is reasonable to assume that players who achieve greater height above the net through performance of basic skills are in advantage. The aim of this study was to examine body height values of volleyball players who competed at world championships 2022.

**Materials and methods**

The sample of subjects consisted of 672 players, subsampled by gender (336 men and 336 women). Univariate analysis of variance was applied to determine body height (BH) by player’s position (setter, opposite, middle blocker, outside hitter, and libero), and performance level (1st – 4th; 5th – 8th; 9th to last). Statistical significance was set at p≤0.05.

**Results**

Regarding player’s position, there are differences both for men (F=60.51, *P*=0.00), and women (F=74.59, *P*=0.00). All positions are statistically different from one another, with exception in female subsample in which middle blockers and opposites represents the same group in terms of BH. The order in values is as follows: middle blockers (2.03±0.05, 1.89±0.05), opposites (2.00±0.06, 1.87±0.06), outside hitters (1.96±0.06, 1.83±0.05), setters (1.92±0.06, 1.80±0.05) and libero players (1.84±0.07, 1.70±0.06), for men and women, respectively. In terms of performance level there are no statistically significant differences.

**Conclusions**

Based on the player’s characteristics and abilities, coaches should assign player a role on the court that would maximize the player’s contribution to the team. In pursuit of elite performance, it is of great importance to differentiate between the trainable and non-trainable qualities of a player. Even though advanced BH represents advantage for each player, for some of the players (middles and opposites), it is absolute requirement.

## A122 DNA methylation-based aging clocks of 59 Olympic Champions: Are they younger or older than their chronological age?

### Zsófia Bábszky

#### Research Center for Molecular Exercise Science, Hungarian University of Sports Science, Budapest, Hungary

##### **Correspondence:** Zsófia Bábszky (radak.zsolt@tf.hu)

*BMC Proceedings 2024*, **18(11)**: A122

**Background**

Because lifestyle, including exercise habits, are modulators of DNA methylation, we suggested that the DNA methylation-based aging clocks of Olympic champions would differ from the mean population.

**Materials and methods**

Fifty-nine Olympic champions voluntarily participated in our study, which was approved by the National Center for Public Health (7147-6/2022EUIG). The control group was a non-Olympic champion group. Blood samples were collected and stored in evacuated tubes containing EDTA as an anticoagulant to determine erythrogram. Blood samples were centrifuged and stored at − 80 °C. Epigenetic clocks are considered highly promising molecular biomarkers of aging. The following epigenetic clocks were used: Hannum’s blood-specific clock, Horvath’s pan-tissue clock, PhenoAge, GrimAge, the DunedinPACE clock, and DNAmFitAge. The telomere length was also evaluated by Horvath’s software which estimates the telomere length from methylation.

**Results**

The first-generation epigenetic clocks are associated with mortality risk Hannum and Horvath clocks, the second-generation clocks are the prediction of aging-related morbidity, disability, and mortality by DNAm biomarkers is enhanced by the incorporation of physiological data like PhenoAge, GrimAge, and DunedinPACE. DNAmFitAge is a third-generation epigenetic clock sensitive to the level of physical fitness. There is a relationship between chronological age and DNA methylation-based aging clocks, and except Hannum and DNAmFitAge Olympic champions epigenetic age is younger than their chronological age. When the age acceleration was examined again, Olympic champions had significantly decelerated DNA methylation-based aging, except Hannum and DNAmFitAge. We have calculated the telomere length from the methylation data. Olympic champions have significantly longer telomeres than non-champions.

**Conclusions**

It is advisable to conduct further investigations into other epigenetic clocks, as their results may vary. An epigenetic clock provides insights into this process only from a singular perspective.

## A123 Determinants of body image in reproductive-age women: A cross-sectional investigation

### Olívia Dózsa-Juhász^1^, Viktória Prémusz^2^, Pongrác Ács^2^, Alexandra Makai^2^, Márta Hock^1^

#### ^1^Faculty of Health Sciences, Institute of Physiotherapy and Sport Sciences, University of Pécs, Pécs, Hungary; ^2^Physical Activity Research Group, Szentagothai Research Centre, University of Pecs, Pécs, Hungary

##### **Correspondence:** Olívia Dózsa-Juhász (olivia.juhasz@etk.pte.hu)

*BMC Proceedings 2024*, **18(11)**: A123

**Background**

Body image (BI) encompasses self-perception and attitudes. This study investigates the impact of body mass index (BMI), premenstrual symptoms (PMS), physical activity (PA), sitting and sleeping durations, and sleep quality (SQ) on BI, discerning their influence on positive or negative BI development.

**Materials and methods**

291 women (aged 18-45) participated in the online cross-sectional research. Surveys, including the Body Appreciation Scale, Global Physical Activity Questionnaire, Sleep Quality Scale, and Premenstrual Assessment Form-Short Form, were applied to the complex questionnaire. IBM SPSS 28.0 analyzed the data with *p*<0.05 significance.

**Results**

Participants (average age: 27.16±0.36 years) had an average BMI of 22.77±3.93 kg/m2. Body Appreciation Scale scores averaged 44.64±10.99 points, sleep quality 35.23±12.55 points, and PMS 29.50±9.57 points. Weekly, they spent 206.00±455.44 minutes in PA and 3015.56±1428.86 minutes sitting. Multivariate linear regression (R2=0.238; F=22.321; *p*<0.001) confirmed BMI (*p*<0.001), PMS (*p*<0.001), SQ (*p*<0.001), and physical inactivity (*p*=0.008) significantly affect BI.

**Conclusions**

BMI, PMS severity, sleep, and inactivity notably impact BI development in women, underscoring their importance for women's health and quality of life. These factors collectively contribute to the overall well-being and daily functioning of women, highlighting the intricate relationship between physical health, psychological well-being, and quality of life. By addressing BMI, managing PMS symptoms effectively, prioritizing restful sleep, and promoting physical activity, women can not only mitigate the risk of negative BI development but also enhance their overall quality of life, fostering a sense of vitality and fulfillment in their daily experiences.

**Acknowledgment**

Supported by the únkp-23-2 new national excellence program of the ministry for culture and innovation from the source of the national research, development and innovation fund. This research was sponsored by the National Research, Development and Innovation Fund of Hungary (TKP-2021-EGA-10).

## A124 Physical form as panacea: Fitness in elderly is related to cognition, quality of life and brain metabolism

### Dragana Zanini, Nikola Todorović, Darinka Korovljev, Valdemar Štajer, Sergej M. Ostojić

#### Faculty of Sport and Physical Education, University of Novi Sad, Novi Sad, Serbia

##### **Correspondence:** Dragana Zanini (zadragupsi@yahoo.com)

*BMC Proceedings 2024*, **18(11)**: A124

**Background**

The growing number of older people is associated with an increasing risk of age-related diseases. Old age portrays decline in cognitive performance and functional movement limitations, conjoined with altered and disrupt brain circuits, and brain energy deficiencies.

**Materials and methods**

Extracted from the RCT double blind study, forty participants older than 70 y. of age (F = 20, mean age 74.6 ± 4.9) were included in correlational study aimed to address association between fitness on the one hand and cognition, quality of life and brain metabolism on the other hand. Senior Fitness Test and additionally, handgrip strength test were used as fitness indicators. Cognitive domain was assessed trough score of global cognitive function (MMSE; and ADAS-cog-11) and measures of specific cognitive functions (processing speed, visual search and scanning, memory, executive function, working memory). Subjective data were obtained for quality of life (SF-36), and quality of sleep (SQS). Subsample of 10 volunteers underwent magnetic resonance spectroscopy for the brain metabolism scanning.

**Results**

Flexibility of lower extremities (sit and reach test) and upper extremities (back scratch test) is positively correlated with the speed of cognitive processing, short-term memory, working memory and executive functions. Aerobic endurance (two-minute step test) is associated with short-term memory and executive functions. Agility and dynamic balance (8-foot test) is positively linked with global cognitive function and memory, and with subjective experience of physical functioning. Interestingly, strength tests neither show significant association with cognition, nor subjective measures of quality of life. Elderly with higher BMI demonstrated poorer executive functions. Sit and reach test, step test, and 8-foot test are indicative for empowering brain metabolism, with consistent positive association with Choline found in left parietal-mesial grey matter.

**Conclusions**

Physical fitness has potential to boost cognitive functions, underlying neurometabolism, and quality of life in old people.

## A125 Serve reception techniques used of elite female volleyball players

### Lana Bajić, Duško Cvijović, Sunčica Poček

#### Faculty of Sport and Physical Education, University of Novi Sad, Novi Sad, Serbia

##### **Correspondence:** Sunčica Poček (suncica.pocek@fsfvns.edu.rs)

*BMC Proceedings 2024*, **18(11)**: A125

**Background**

Serve reception in volleyball is a basic skill, crucial element of the attack organization. Ideal serve reception allows setter, and subsequently attacker numerous attacking options. There are different techniques possible to perform serve reception, with underhand pass being the most efficient. The aim of this study was to examine serve reception techniques used of elite female volleyball players.

**Materials and methods**

This research was conducted on 204 serve receptions, performed by National teams of Serbia and Italy, gold and silver medalists from World Championship of 2018 for female volleyball players. In order to examine differences between two teams and serve reception techniques used of elite female volleyball players we applied chi square test, with statistical significance set at p≤0.05.

**Results**

There are no statistically significant differences between the National teams of Serbia and Italy in the frequency of usage of different types of serve reception techniques (χ2=2.801, *p*=0.592). Within both national teams, there are differences in the type of technique used when receiving a serve, where the technique of underhand pass is represented by 84.3%, while in sporadic cases, the technique of overhand pass is represented by 3.4% and volleyball dives and rolls by only 1.5% (10.8% were errors).

**Conclusions**

In contemporary game, elite level volleyball players are exclusively organized by 5:1 composition (5 attackers and 1 setter). The main feature of the 5:1 composition is the assignment of specific tasks to each player on the court. Three players are in charge of serve reception on the court, two outside hitters and a libero player. The most efficient way and frequency of usage of serve reception is through underhand pass technique, which was confirmed by the results of this study.

## A126 Implementing a physical activity game to engage students in a learning environment

### Panagiota Chrysopoulou, Katerina Papadimitriou, Efstratia Tsitskari, Dimitra Kotsi, Ourania Matsouka, George Costa

#### Department of Physical Education and Sport Science of Democritus University of Thrace, Komotini, Greece

##### **Correspondence:** Ourania Matsouka (oumatsou@gmail.com)

*BMC Proceedings 2024*, **18(11)**: A126

**Background**

Previous research has indicated that incorporating playfulness in learning can effectively enhance students' levels of knowledge and motivation. Games can provide students with an exciting opportunity to channel their energy and enjoy the learning process when combined with physical engagement. The primary objective of this study was to compare the effectiveness of a physical activity game to a traditional teaching method in English language instruction.

**Materials and methods**

The study comprised 35 first-grade students from the Music School of Komotini. 19 students were taught using the traditional method, while 16 students were taught using a physical activity game. Both methods aimed to teach ten English adjectives. In the traditional method, students had to complete vocabulary exercises after attending a vocabulary presentation. On the other hand, the physical activity game was a modified version of the traditional game hopscotch, which included three types of vocabulary questions. The researcher collected data by conducting a pre-intervention and post-intervention test and qualitative observation. The data were analyzed using IBM SPSS Statistics 27.

**Results**

According to the results obtained, both teaching methods had a positive impact on the students' knowledge levels. Although the physical activity game group performed slightly better, there was no statistically significant difference in their knowledge levels when compared to the traditional group. The results were consistent for both male and female students. Nonetheless, students who took part in the physical activity game displayed a greater level of motivation towards actively participating in the learning process.

**Conclusions**

According to the study, using playful teaching methods can be as effective as, or even more effective than, traditional methods. Playful methods can also motivate students to participate more actively in the learning process. The study's findings have important implications for educators who want to improve their students' learning outcomes, especially in subjects where engagement and motivation are crucial.

## A127 Effectiveness of EMMETT technique on Iliotibial band tightness in football players

### Teo Radić^1^, Jelena Paušić^2^, Mario Rak^3^

#### ^1^Department of Physical Medicine and Rehabilitation, General Hospital “Šibenik”, Šibenik, Croatia; ^2^Department of Kinesitherapy and Sports recreation, Faculty of Kinesiology, University of Split, Split, Croatia; ^3^Department of Conditional and Strength Training, Croatian Football Club “Šibenik”, Šibenik, Croatia

##### **Correspondence:** Teo Radić (teo.radic23@gmail.com)

*BMC Proceedings 2024*, **18(11)**: A127

**Background**

EMMETT technique is a method where, by applying light pressure on a person's body for 5-20 seconds, muscle relaxation, flexibility and ROM are improved. The ITB is the longest thick tendon which, due to its anatomical structure, is mostly tight and limits flexibility in the pelvis and lower extremities. With all the above, it is one of the most common overused injuries among runners, including football players. The aim of this study was to determine whether there are differences in flexibility in the ITB after applying the EMMETT technique.

**Materials and methods**

The sample was conducted of 50 male physically active young football players (25 cadets aged 2007/08 and 25 juniors aged 2005/06). At random, we took 13 cadets and 13 juniors as an experimental group, and 12 cadets and 12 juniors as a control group. Three measurements of the ROM were measured before and after applying two EMMETT corrections (ITB and ITB/Sartorius) in the experimental group, while no therapy was performed in the control group. ROM was measured with the modified Ober test and with EasyAngle goniometer. Interrater and interrater reliability of the recorded hip range of motion were calculated using intra-class correlation coefficients (ICC) (3,1).

**Results**

Interrater and interrater reliability were good to excellent for both hip ROM measurements. The results showed that the ROM in the experimental group of juniors and cadets was significant different after the applying of EMMETT technique compared to the control group that did not perform the technique itself.

**Conclusions**

Therefore, the presented results show that the EMMETT technique has an acute effect in increasing flexibility in the ITB, which indicates that in the future we should try to use the EMMETT technique as a prevention of overuse syndromes and possible pelvic and lower extremities injuries.

## A128 Comparison of motor skills among junior triathlon athletes

### Ádám Balog^1^, Zoltán Alföldi^2^, Anna Horváth-Pápai^1^, Ferenc Ihász^3^

#### ^1^Faculty of Health Sciences, Doctoral School of Health Sciences, University of Pécs, Pécs, Hungary; ^2^Faculty of Health and Sport Sciences, Széchenyi István University Győr, Győr, Hungary; ^3^Faculty of Pedagogy and Psychology, Institute of Sports Science, Eötvös Lóránd University, Szombathely, Hungary

##### **Correspondence:** Ádám Balog (balogadam85@gmail.com)

*BMC Proceedings 2024*, **18(11)**: A128

**Background**

In our study, we investigated the motor skills of junior triathletes training in clubs in four different regions of the country. The aim of the study was to compare the performance of children of different ages and genders.

**Materials and methods**

The study included 153 participants (60 girls, 93 boys). Conditioning ability was measured using the NETFIT test battery tasks. Coordination skills were measured on the unified cycling skill course. Joint mobility was measured using the Functional Movement System (FMS) tasks. Data were analyzed using a two-sample t-test and one-way ANOVA with random error at the *p*<0.05 level.

**Results**

When comparing children of the same age group by gender, no significant differences were found in any of the characteristics. However, when comparing the results of children of the same sex in different age groups, a number of differences were found. In the results for toddlers and prepubertal age groups, significant differences were found for boys and girls in all anthropometric characteristics, in the long jump, hanging, 30 m flat run, shuttle run, and in the time and error rates of the cycling skill course. Significant differences were found in the estimated aerobic capacity of age groups, gender, and regions for boys in each age group when comparing region two with regions one and four. In the 30 m flat run, we found a significant difference in the results for boys in age group one in region two compared to all other regions. Also, for girls, the results of region two were significantly different from those of regions one and four.

**Conclusions**

The results of our research confirmed that absolute performance increases with age. There is no difference between the sexes in the younger age group, and this is also true for the prepubertal age group.

## A129 The relationship between sitting time with exercise habits, body composition and bone density

### Ferenc Győri^1,2^, Beáta Vári^3,4^, Szabolcs Halasi^15^, Gábor Herczeg^1^, Tamás Berki^6^

#### ^1^Sport Science Research Group, “Gál Ferenc” University, Szeged, Hungary; ^2^Institute of Physiotherapy and Sports Science, University of Pécs, Pécs, Hungary; ^3^Doctoral School of Health Science, University of Pécs, Pécs, Hungary; ^4^Institute of Physical Education and Sports Science, University of Szeged, Szeged, Hungary; ^5^Department of Art and Physical Education, Hungarian Language Teacher Training Faculty, University of Novi Sad, Subotica, Serbia; ^6^Department of Physical Education Theory and Methodology, Hungarian University of Sports Science, Budapest, Hungary

##### **Correspondence:** Ferenc Győri (gyori.ferenc@gfe.hu)

*BMC Proceedings 2024*, **18(11)**: A129

**Background**

People who sit a lot because of their work are more exposed to the dangers caused by inactivity (e.g. metabolic syndrome, deteriorating bone quality) than others. Our research examines how much people who sit a lot due to their work and in their free time compensate for it with physical activity and how much this is reflected in their body composition or bone quality.

**Materials and methods**

The basis of our study was a survey of office workers. The respondents were employees of the state offices of 7 settlements in Hungary (*N*=307). Our questions related to the duration of sitting time at work and at home, as well as the frequency of sports and their nature. We used an InBody 230 device to measure body composition, while a SONOST 3000 device was used to measure bone density (BQI, T-score). To analyze our data, we used a t-test, a one-way analysis of variance and correlation calculation.

**Results**

Athletes who exercised more and/or at a higher level had higher BQI, lower T-score and body composition parameters indicative of obesity. We also found significantly lower values for obesity among those who perform vigorous, sweat inducing, but non-sports activities. We also found no correlation between the time spent sitting at work and the test parameters, but there was a correlation with the time spent sitting in leisure time (e.g. using the computer or watching TV).

**Conclusions**

Our results reflect those sports and physical activity, as part of a lifestyle, have an impact on many variables related to body composition. The frequency and duration of sports, as well as the level of activity, are also determining factors. Our research has shown that high sitting time at work is harmful, especially if it is combined with sitting time at home, i.e. it becomes a sedentary lifestyle.

## A130 Analysis of situational efficiency indicators between the champions of the Champions League in seasons 2021/2022 and 2022/2023

### Ivan Belčić, Ivan Ljubičić, Ivan Krakan

#### Faculty of Kinesiology, University of Zagreb, Zagreb, Croatia

##### **Correspondence:** Ivan Belčić (ivan.belcic@kif.hr)

*BMC Proceedings 2024*, **18(11)**: A130

**Background**

Analyzes of football matches and competitions and the recording of performance and efficiency indicators enable consistent and reliable quantification of key events and consequently quantitative and qualitative feedback, and thus are the subject of a large amount of research. By analyzing the situational effectiveness of the game, the coaches gain an insight into the objective state of their team, and thus the data for improving the technical and especially tactical elements of the game.

**Materials and methods**

All group and cup matches (13) of the Champions League winners in seasons 2021/2022 (Real Madrid) and 2022/2023 (Manchester City) are included in this research. The sample consists of 21 variables of technical-tactical elements of defense and attack phase in matches that influence situational efficiency in football. T-test for independent samples was used to determine differences with statistical significance set to *p*<0.05.

**Results**

The results show a significant difference (*p*<0.05) between the Champions League winners in 3 variables out of 21 variables. Significance is found in variables possession (*p*=0.0006), percentage of accurate passes (*p*=0.025) and blocked shots (*p*=0.049).

**Conclusions**

Real Madrid had a statistically higher number of blocked shots in defense than Manchester City, which shows a higher number of defensive tasks. Manchester City was more successful in almost all indicators of situational efficiency compared to Real Madrid, while the statistically significant difference was in variables possession, percentage of accurate passes and blocked shots. For complete results and a more detailed insight into the situational efficiency between Champions League winners, a larger number of seasons should be taken in the sample.

## A131 Application of portfolio matrix for resource allocation purposes in sports: The case of Hungary

### Márk Hoffbauer^1^, Pongrác Ács^1^, Miklós Stocker^2^

#### ^1^Faculty of Helath, University of Pécs, Pécs, Hungary; ^2^Corvinus University, Budapest, Hungary

##### **Correspondence:** Márk Hoffbauer (hoffbauer.mark@pte.hu)

*BMC Proceedings 2024*, **18(11)**: A131

**Background**

Sports can have several aspects in the society, and therefore, government-related financial contribution can also be justified, the resource allocation decisions of the contributor are always a very actual topic, however.

**Materials and methods**

In this paper, we try to create a modified portfolio matrix tool for the resource allocation purposes, which can be used at a national, regional, municipal or organizational level. In the primary research, top managers of selected major sport clubs have been surveyed with a CAPI-method questionnaire, covering 40+ sports. From the received answers, the sport portfolio matrix has been created, which shows the ranking of evaluated sports according to the basis of future vision and the current impact.

**Results**

It is not surprising that soccer turned out to be the highest ranked sport, but it is interesting to see archery or surfing in the top-12. We also modelled the Hungarian governmental expenditure on competitive sport in 2022 according to the sport portfolio matrix.

**Conclusions**

It shows a comprehensive resource allocation solution, which favors present performance, and therefore, should be applied complemented with a strategic resource allocation tool as well.

## A132 Seasonal changes of body fat percentage in elite soccer players

### Nikola Andrić^1^, Aleksandar Karać^1^, Radenko Pantović^2^, Alen Ninkov^2^, Bogdan Ilić^2^, Marko Stojanović^1^

#### ^1^Faculty of Sport and Physical Education, University of Novi Sad, Novi Sad, Serbia; ^2^Trenažna ekspertiza, Novi Sad, Serbia

##### **Correspondence:** Marko Stojanović (marko.stojanovic@fsfvns.edu.rs)

*BMC Proceedings 2024*, **18(11)**: A132

**Background**

Body fat percentage is an important aspect of fitness for soccer players as excess adipose tissue acts as inert load likely detrimental to physical performance. In addition, excess body fat predisposes players to a heightened risk of injury. The aim of this study was to examine changes of body fat percentage during both preseason and competitive season.

**Materials and methods**

Twenty-three male professional soccer players (24.63±3.71 yrs., 186.00±4.53 cm, 78.40±6.35 kg), all members of soccer team competing in Serbia first division, participated in the study. All the players were team members for at least 2 seasons and participated in 9+ hours of soccer training in the last 20 weeks. All of them were healthy, medication-free and without any special diet or supplementation during study. This longitudinal, observational study assessed body fat percentage using skinfold measures at 4 time points (SPS - starts of preseason; EPS - end of preseason; MS - middle of first part of the season; FS - finish of the first part of season) of competitive season 2022/2023. A one-way ANOVA test was used to analyze the seasonal variation at the four-separate timepoints, with *p* value set at 0.05. Post-hoc analyses were analyzed with Bonferroni correction factor.

**Results**

There were significant differences between groups (*p* = 0.014) with post hoc test revealing significant difference between SPS and both MS (*p* = 0.039) and FS (*p* = 0.023).

**Conclusions**

Study findings suggest that there are significant fluctuations in body fat percentage during season in top level soccer players. In addition, it seems that preseason is not long enough or is not intensive enough to produce a significant drop in body fat percentage. However, accumulation of additional training sessions during the season produces stable and favorable alterations in body fat percentage.

## A133 Correlations between smartphone use and physical activity among Hungarian university students

### Bettina Horváthné Tóth^1,2^, András Salamon^12^, Gabriella Császár^1^

#### ^1^Department of Physiotherapy, Institute of Physiotherapy and Sport Science, Faculty of Health Sciences, University of Pécs, Zalaegerszeg, Hungary; ^2^Doctoral School of Health Sciences, Faculty of Health Sciences, University of Pécs, Pécs, Hungary

##### **Correspondence:** Bettina Horváthné Tóth (bettina.toth@etk.pte.hu)

*BMC Proceedings 2024*, **18(11)**: A133

**Background**

Not only in Hungary, but throughout the world, the number of smartphone users is increasing year by year. Previous research has shown that smartphone addiction can be associated with lower physical activity and musculoskeletal problems. The aim of our research is to examine the level of smartphone addiction among Hungarian university students and to find a correlation between the amount of smartphone use and physical activity.

**Materials and methods**

A total of 409 (314 female, 95 male) Hungarian undergraduate students attended in this study (average age: 21.44, SD 2.78). We used convenience sampling. The participants filled out an online questionnaire. The online questionnaire included: smartphone addiction (Smartphone Addiction Scale), physical activity (International Physical Activity Questionnaire short form- IPAQ-SF), smartphone usage habits (screen time, most used application). Statistics: descriptive statistics, correlation calculation, Kruskal-Wallis test (SPSS 24 v., *p* <0.05).

**Results**

Participants use their smartphone for an average of 4.43 hours a day and spend an average of 10.45 hours in physical activity a week. The most frequently used application was Messenger. Students who scored higher on the SAS performed significantly less physical activity in one week. (*p*=0.016, *r*=-0.119). We also found a significant correlation between the number of hours spent sitting per day and smartphone addiction. (*p*=0.028, *r*=0.109). Finally, we found no difference between the levels of physical activity and the total score of smartphone addiction.

**Conclusions**

A weak correlation can be discovered between smartphone addiction and physical activity; however, further research is needed to confirm this.

**Acknowledgment**

This research was sponsored by the National Research, Development and Innovation Fund of Hungary (TKP-2021-EGA-10).

## A134 Kinematic determinants of repeated transition from underwater to surface swimming

### Jelena Stošić^1^, Xiao Qiu^1^, Stefan Fuhrmann^2^, Santiago Veiga^1^

#### ^1^Departamento de Deportes, Universidad Politécnica de Madrid, Madrid, Spain; ^2^Hamburg Olympic Training Centre, Hamburg, Germany

##### **Correspondence:** Santiago Veiga (santiago.veiga@upm.es)

*BMC Proceedings 2024*, **18(11)**: A134

**Background**

Recent research on competitive swimming focused on transition performance, as an important segment of swimming races. The way swimmers perform transition from underwater to surface swimmers was shown to be very individual as large inter-subject variability was shown recently. Therefore, the aim of the study was to test intra-subject variability of transition performance.

**Materials and methods**

International and national level swimmers (*n*=26, 10 females; 16.04±1.99 years, 186.08±7.08 cm and 79.77±10.52 kg), performed 10 x 25 m all – out at front-crawl from a starting block. Two lateral underwater cameras operating at 50 Hz filmed transition phase. Stability of transition performance was assessed using correlation coefficient between the mean and standard deviation of the 1) cyclic kinematics: transition stroke length, transition stroke rate and transition velocity, 2) segmental kinematics (trunk and body inclinations, body depth) and 3) coordinative parameters (discrete relative angles). In addition, linear regression model was used to assess what sets of variables determine the best transition performance.

**Results**

Mean values of each kinematic variable were only related to their standard deviation for the body depth (*r*=0.506, *p*=0.008) and stroking length (*r*=0.553, *p*=0.026), while no statistical significance was shown for the cyclic, segmental, or coordinative parameters. The best predictor of transition performance was linear regression model in which transition greater stroke length and larger discrete relative phase 1 (indicating lower time gaps between propulsive arms activities) were combined (*p*=0.006).

**Conclusions**

Results showed no relationships between intra-subject variability and transition performance in competitive swimmers indicating consistency across trials, which contradicts predictions based on large inter-subject variability. On the other hand, transition performance was related to greater stroke lengths and lower time gaps. This partially differed to previous finding in transition from a push start and it could be related to the positive effect of dive start on forward velocity.

## A135 The role of chronic exercise on attention and working memory in adolescents and older adults

### Alexandru Octavian Mihai Stoica

#### West University of Timisoara, Timisoara, Romania

##### **Correspondence:** Alexandru Octavian Mihai Stoica (stoicaefs@yahoo.com)

*BMC Proceedings 2024*, **18(11)**: A135

**Background**

Good WM performance was associated with improved cognitive function. Recent studies show that exercise causes functional changes in specific WM-related brain regions.

In this systematic review, we aim to investigate the effects of exercise on attention and working memory in adolescents/adults.

**Materials and methods**

In the electronic databases PubMed, Web of Science, MEDLINE and ERIC we searched for studies investigating the effects of physical activity on attention, executive function and/or academic performance, all published between 1997-2024. We then grouped the information as follows: four subdomains of executive function (inhibition, working memory, cognitive flexibility, and planning), three subdomains of attention (selective, divided, and sustained), and three subdomains of academic performance (reading, mathematics, cross-disciplinary).

**Results**

Domain analyses showed that WM measures scored significantly given the effect of exercise on WM (Q(5) = 16.63, *p* = 0.005). The effect size of the outcomes on these domains (Cohen's d = 0.59) was larger than that of the n-back (Cohen's d = 0.55), DSF (Cohen's d = 0.38), and DSB (Cohen's d = 0.19) outcomes. Exercise type significantly influenced the effect on WMC (Q(3) = 27.10, *p* < 0.001).

**Conclusions**

In conclusion, chronic exercise is a promising way to improve WMC in adolescents/adults We found that chronic exercise can lead to improved WMC in older adolescents/adults, provided it is performed at moderate intensity (motor actions with cognitive involvement or chronic resistance exercise) for 45-60 min performed 3-4 times per week for 8-12 weeks. The recommended chronic exercise is based on elements of running, sports games, and physical elements, including the body-mind relationship of moderate intensity (at a heart rate up to max. 140 -160 beats/min, for 45-60 min. at a frequency of 3 times a week for more than 6 months.

## A136 Analysis of pulse pattern indicated by Fit Dance workout (Pilot study)

### Anna Horváth-Pápai^1^, Zoltán Alföldi^2,3^, Sándor Gergely Gabnai^4^, Ádám Balog^1^

#### ^1^Doctoral School of Health Sciences, University of Pécs, Pécs, Hungary; ^2^Faculty of Health Sciences, Institute of Physiotherapy and Sport Science, University of Pécs, Pécs, Hungary; ^3^Faculty of Health and Sport Sciences, Széchenyi István University, Győr, Hungary; ^4^Illés Academy, Szombathely, Hungary

##### **Correspondence:** Anna Horváth-Pápai (papai.anna@gmail.com)

*BMC Proceedings 2024*, **18(11)**: A136

**Background**

Aerobic is one of the most popular forms of exercise today. The aim of the present study is to examine heart rate values indicated by aerobic dance exercise among young female university students.

**Materials and methods**

We analyzed the heart rate values of 16 university students, aged (20.7±1.78 yrs.), with a relative body fat mass of (35.4±7.9 kg). The Fit Dance workout lasted 50 minutes, and we divided it into five sections. The beat of the music was 130-136 beat per minute. During the test the university students wore a Polar H10 heart rate sensor belt. We used the HA7 mood scale to define the mood changes before and after the aerobic training. One-way analysis of variance was used to examine differences between groups. The level of significance was set at 0.05.

**Results**

We found a statistically significant difference between the heart rate averages of the first (132.4±17.46) and second part 160.6±13.56, F(2.56), 3.845 *p*<0.0000), and between the maximum heart rate averages (156.5±12.5 - 173.0±9.81, F(2.56), 4.213 *p*<0.0002. The highest pulse values are during dynamic dance and strengthening training sections. The mood before and after the training session and the pulse pattern vary. Sometimes the mood change is opposite to the physiological response and sometimes it coincides.

**Conclusions**

Based on the body composition examination the participants were generally overweight or obese. Observing the values of the pulse pattern, it can be stated that the group is quite diverse in terms of fitness level. Following the heart rate pattern shows that a well-structured aerobic training session induces a load of suitable intensity, which contributes to the development of aerobic capacity.

## A137 The potential use of virtual reality in the treatment of anxiety in athletes

### Melinda Trpkovici^1,2,3^, Krisztina Rácz^1^, Viktória Prémusz^2,3^

#### ^1^Doctoral School of Health Sciences, Faculty of Health Sciences, University of Pécs, Pécs, Hungary; ^2^Faculty of Health and Sport Sciences, Széchenyi István University, Győr, Hungary; ^3^Physical Activity Research Group, Szentágothai Research Centre, Pécs, Hungary

##### **Correspondence:** Melinda Trpkovici (melinda.bite-trpkovici@pte.hu)

*BMC Proceedings 2024*, **18(11)**: A137

**Background**

One of the most successful techniques is “stress inoculation” training (SIT), which is increasingly being used to reduce anxiety and improve athletic performance. The purpose of our research was to to examine to what extent the stress situation created by us in virtual reality elicits psychological responses from the athletes, compared to what the athletes experience in a competitive match.

**Materials and methods**

The sample consisted of 24 female athletes (age average was 18.71 ± 5.42); out of the total sample, were elite 9 basketball players, 8 were table tennis players and 7 were handball players. Those participants completed the Sports Anxiety Questionnaire, which is suitable for measuring anxiety in a high-stakes situation, as well as for determining the level of concentration and self-confidence experienced during a match. Furthermore, in the virtual reality we created, the athletes were placed in a sports environment that contained stress factors that are scientifically proven to induce stress in the athletes. SPSS 28.0 software was used for statistical analysis, where p≤0.05 values were considered significant.

**Results**

Our results show that the sports stress situation created in virtual reality elicits the same level of stress responses from the athletes as those experienced by the athletes in a competitive match. No significant difference can be detected in any factor between the total scores of the test completed after VR and the test completed after the match (*p*> 0.05).

**Conclusions**

Based on the results, we can conclude that the sports stress situation created by virtual reality can induce the same level of stress in the athletes compared to the match, so it can be suitable for the development of athletes' stress management and the device can be a huge advantage in the process of sports psychology preparation.

**Acknowledgment**

This research was sponsored by the National Research, Development and Innovation Fund of Hungary (TKP-2021-EGA-10)

## A138 Physical literacy as a determinant of physically active lifestyle in association with well-being, a cross-sectional study

### Alexandra Makai

#### Faculty of Health Sciences, University of Pécs, Pécs, Hungary

##### **Correspondence:** Alexandra Makai (alexandra.makai@etk.pte.hu)

*BMC Proceedings 2024*, **18(11)**: A138

**Background**

Physical literacy (PL) refers to the ability to establish lifelong adherence to physical activity (PA) for a healthier life. The purpose of the current study was to introduce PL testing in Hungary, examine Physical Literacy of adults in association with physical activity patterns and well-being.

**Materials and methods**

A cross-sectional, prospective cohort study was carried out among 244 young adults. We measured PL by Holler’s Physical Literacy Questionnaire (PLQ), physical activity (PA) by the Global Physical Activity Questionnaire (GPAQ), and quality of life (QoL) by the World Health Organization Five Well-Being Scale (WBI-5). Descriptive statistics and Spearman’s rank correlation coefficients were used. The subgroup differences were tested using Mann-Whitney U test. IBM SPSS 28.0 software was used for data analysis and the level of significance was set at *p*<0.05.

**Results**

Average age of the participants in the study was 24.51 (SD 6.44) years. The rate of male participants was 13.04% and the females was 86.96%. 77.39% of them did some sport at least three times a week. The average well-being index was 8.63 (SD 2.91). The PL subscales as motivation, self-efficacy, active lifestyle and self-confidence were showed significant correlation (*r* = 0.289-0.591; *p*<0.001). PA (*r* = 0.227-0.652; *p*<0.001) and well-being (*r* = 0.339-0.474; *p*<0.001) showed significant association with all domains of PL.

**Conclusions**

Adults who are motivated and feel able to do sports and are knowledgeable in the PL live a more active life and demonstrate better HRQoL. The findings of our cross-sectional study proved the association between well-being, PA and PL, what could help to develop appropriate strategies and intervention programs for the Hungarian adult population.

**Acknowledgment**

This research was sponsored by the National Research, Development and Innovation Fund of Hungary (TKP-2021-EGA-10).

## A139 The change in the popularity of online workouts among the Hungarian population during the pandemic period

### Evelin Derkacs^1,2^, Alexandra Makai^1,2^

#### ^1^Faculty of Health Sciences, University of Pécs, Pécs, Hungary; ^2^Doctoral School of Health Sciences, Faculty of Health Sciences, University of Pécs, Pécs, Hungary

##### **Correspondence:** Evelin Derkacs (evelin.derkacs@etk.pte.hu)

*BMC Proceedings 2024*, **18(11)**: A139

**Background**

The restriction of outdoor activities and mobility has influenced exercise habits and consistency, but today's modern technology has opened new opportunities for those who want to engage in sports. In our research, we examined the changes in the popularity of online workouts during the pandemic.

**Materials and methods**

Our study is a representative longitudinal trend analysis (*N* = 3600), conducted among the Hungarian adult population (regular athletes, *N* = 729; non-athletes, *N* = 2712; participants in online workouts, *N* = 159) during the first three waves of the COVID-19 pandemic. The questionnaire included questions regarding sports habits, physical activity (IPAQ-SF), and well-being (WBI-5). Female respondents accounted for 51.86% (*N* = 1867) of the total, while males constituted 48.14% (*N* = 1733), with a mean age of 43.49±15.13 years. Descriptive and inferential statistical methods (chi-square test) were used during data processing. A significance level of *p* < 0.05 was considered significant.

**Results**

During the first wave of the pandemic, the proportion of regular exercisers decreased to 21.67%, among them 21.98% of them opting for online workout methods. Throughout the three waves of the pandemic, significantly more women (*p*<0.001), individuals aged 18-29 (*p*<0.001), those with higher education (*p*<0.001), and residents of the capital city (*p*<0.001) participated in online workouts.

**Conclusions**

The pandemic has altered exercise habits, with the popularity of online workouts increasing. Further research is needed to examine the changes in online workout preferences since the onset of the pandemic.

**Acknowledgment**

This research was sponsored by the National Research, Development and Innovation Fund of Hungary (TKP-2021-EGA-10).

## A140 A study of the effectiveness of core training with EMG in recreational kayak and canoe athletes

### Csaba Melczer, Tamás Laczkó, Bence Raposa, Péter Tardi, Dorina Czakó

#### Faculty of Health Sciences, University of Pécs, Pécs, Hungary

##### **Correspondence:** Csaba Melczer (csaba.melczer@etk.pte.hu)

*BMC Proceedings 2024*, **18(11)**: A140

**Background**

In our research we studied junior kayak-canoe athletes. We examined trunk muscle strength and dynamic balance in the athletes' lower limbs. We measured muscle activity using surface electromyography (EMG). We also examined how the training program we developed affected the athletes' performance.

**Materials and methods**

The type of research is a quantitative longitudinal prospective study conducted between January and April 2021. The number of participants in the sample was 12 (aged 14-18 years). The exercise program lasted 15 weeks, 45 minutes twice a week. Subjects went through assessment of lower limb dynamic balance (Stork test, Star Excursion balance test), and strength of core muscles (plank test). Statistical test used: one-sample T-test.

**Results**

Strength of core muscles was significantly improved by the exercise program (plank test *p* < 0.001). Lower limb balance tests showed a significant improvement. Star test for the right anterior, anteromedial, medial, posterior, lateral (*p* < 0.001), posteromedial direction (*p* = 0.005). All directions were also considered significant for the left side. Improvement can be seen. EMG examination on a stable surface showed significant results on the left side m. obl. externus. (*p* = 0.023), internus. (*p* = 0.044), right m. obl. int. (*p* = 0.022), M. longissimus (*p* = 0.049). EMG test measured on an unstable surface on the right m. obl. int. abd. (*p* = 0.010), left m. obl. int. abd. (*p* = 0.011) significant improvement.

**Conclusions**

Overall, the training program had a positive effect on the athlete's ability to balance trunk muscle strength and muscle activity as measured by EMG.

**Acknowledgment**

This research was sponsored by the National Research, Development and Innovation Fund of Hungary (TKP-2021-EGA-10).

## A141 Neck pain and its association with physical activity, quality of life and perceived stress level

### Nikolett Tumpek^1,2^, Marcell Lendvay^3^, Rebeka Orbán^4^, Melinda Járomi^1^

#### ^1^Faculty of Health Sciences, Institute of Physiotherapy and Sport Science, Pécs, University of Pécs, Pécs, Hungary; ^2^Faculty of Health Sciences, Doctoral School of Health Sciences, University of Pécs, Pécs, Hungary; ^3^Medical School, Department of Rheumatology and Immunology, University of Pécs, Pécs, Hungary; ^4^Faculty of Humanities and Social Sciences, Doctoral School of Developmental and Clinical Psychology, University of Pécs, Pécs, Hungary

##### **Correspondence:** Nikolett Tumpek (tumpek.nikolett@pte.hu)

*BMC Proceedings 2024*, **18(11)**: A141

**Background**

Worldwide, including Hungary, the occurrence of spinal complaints is very common, with a plethora of possible causes. Our research aims to investigate what factors influence the prevalence and extent of neck pain. The aim of our study was to investigate the association of neck pain in the adult population with physical activity, quality of life and perceived stress levels.

**Materials and methods**

After recording sociodemographic data, standard questionnaires such as the VAS (Visual Analogue Scale), NPDS (Neck Pain and Disability Scale), SF-36 (36-Item Short Form Health Survey), IPAQ-SF (International Physical Activity Questionnaire-Short Form), and PSS (Perceived Stress Scale) were used. Pearson correlation analysis and independent samples t-test were used for statistical analysis using IBM SPSS 28.0 software. Our results were considered significant at *p*<0.05.

**Results**

Our cross-sectional research was conducted among the population aged 18 and over (*N*=542), 23% of whom were male and 77% were female, with an average age of 31.16(± 14.69) years. 68% of the respondents experienced neck pain in their lifetime, 19% currently have neck pain and 44% had experienced neck pain in the last three months. The total number of diagnosed cases is 35. Our results depict a significant association between perceived stress level and the occurrence of neck pain (*p*<0.00). Regarding quality of life, a significant association for all eight items were found (*p*<0.05). Although, a significant association between physical activity and neck pain could not be demonstrated (*p*>0.05).

**Conclusions**

The prevalence of neck pain among our respondents was extremely high. The incidence of neck pain is negatively influenced by perceived stress level. Quality of life is significantly impaired by the prevalence of neck pain, while no association with physical activity levels was found.

**Acknowledgment**

This research was sponsored by the National Research, Development and Innovation Fund of Hungary (TKP-2021-EGA-10).

## A142 Monitoring fatigue among elite male basketball players

### Dóra Nagy^1^, Kenza Szabó^2^, Stefan Pajić^2^, László Rátgéber^1^

#### ^1^Department of Training Science, University of Pécs, Pécs, Hungary; ^2^Ratgéber Basketball Academy, Pécs, Hungary

##### **Correspondence:** Dóra Nagy (doranagy1975@gmail.com)

*BMC Proceedings 2024*, **18(11)**: A142

**Background**

Monitoring fatigue is important among elite basketball players for optimizing training load and periodization, preventing injuries and reaching the highest performance. It is a demanding challenge to select the most essential and relevant parameters from the extensive data available in the rapidly developing world of performance monitoring. Our study aimed to explore potential correlations between three neuromuscular fatigue monitoring methods to find the most applicable ones.

**Materials and methods**

Elite male basketball players (*N* = 12, 24.15±4.54 yrs.) performed countermovement jumps (CMJ) twice a week, a pulse-oximeter measurement twice a day, and filled in a 10-point Borg rating of perceived exertion scale (RPE) after each training session and game for six weeks. The investigated variables were: CMJ: Modified Reactive Strength Index ((MRSI); Jump Height max (JHm) and average (JHa); Eccentric-concentric Phase duration (ECPd); relative Peak Power (rPP); Countermovement depth (CMd); concentric and eccentric Peak Force (cPF, ePF). Among the data derived from the pulse-oximeter, we examined Heart rate variability (HRV), Recovery, Body balance and Cardiac overtraining indicators. Multiple Correlation Analysis was conducted using SPSS22 software (R 0.4-0.6 moderate, 0.8< strong correlation).

**Results**

Pilot Multiple Correlation Analysis revealed the highest correlation (R2 = 0.78) between RPE evening data and CMd. A correlation was found between RPE evening and JHa, ECPd (R2= 0.45 and 0.51). ECPd showed a moderate correlation with HRV, Body balance, and Cardiac overtraining, while JHm showed a moderate correlation with HRV, Body balance, and Recovery.

**Conclusions**

Our findings can have practical benefits for basketball and strength and conditioning coaches. Results showed a moderate correlation between neuromuscular fatigue measured by the pulse-oximeter and CMJ parameters, which can help reduce the data volume and choose the most relevant parameters for monitoring neuromuscular fatigue among elite male basketball players.

**Acknowledgment**

This research was sponsored by the National Research, Development and Innovation Fund of Hungary (TKP-2021-EGA-10).

## A143 Investigating the relationships between endometriosis-related pelvic pain, pain self-efficacy, perceived stress, quality of life and physical activity

### Zsófia Kovács-Szabó, Márta Hock

#### Faculty of Health Sciences, Institute of Physiotherapy and Sport Science, University of Pécs, Pécs, Hungary

##### **Correspondence:** Zsófia Kovács-Szabó (zsofia.szabo@etk.pte.hu)

*BMC Proceedings 2024*, **18(11)**: A143

**Background**

Chronic pelvic pain affects a significant number of women, especially those suffering from endometriosis. A physically active lifestyle can affect coping with pain and can be related to quality of life. Our study aimed to examine the differences between two subgroups created by the level of pain and to evaluate the correlation between endometriosis-related pelvic pain, health-related quality of life, perceived stress, pain-related self-efficacy, and physical activity in both subgroups.

**Materials and methods**

After recording sociodemographic data, NRS (Numeric Rating Scale), SF-36 (36-item Short Form Survey), GPAQ (General Physical Activity Questionnaire), PSS (Perceived Stress Scale), PSEQ (Pain-related Self-Efficacy) and PPIQ (Pelvic Pain Impact Questionnaire) were implemented. For statistical analysis we used IBM SPSS 28.0 software. Spearman's rank correlation analysis, independent sample’s t-test were conducted. We considered *p*<0.05 as statistically significant.

**Results**

The cross-sectional study was anonymous research conducted in Hungary among reproductive-age women aged 18-50 (34.8±6.7, *n*=240). The sample was divided into 2 subgroups based on NRS values (mild pain: NRS=0-4, *n*=81, moderate or strong pain: NRS=5-10, *n*=159). Our results revealed significant differences between the two subgroups regarding PSEQ SF-36, PPIQ (*p*<0,001) recreational activity (*p*=0,002) and PSS (*p*=0.003), dimensions. In the higher NRS subgroup we have found positive significant correlations between SF-36 social life domain and the moderate and intensive sport (*r*=0.188*, *p*=0.022 (intensive), *r*=0.164*, *p*=0.042) and between the PSEQ and the regular recreational activity (*r*=0.169*, *p*=0,040), and significant negative correlations between the PPIQ and regular recreational activity (*r*=-0.165, *p*=0,044).

**Conclusions**

Engaging in regular recreational activities and leading an active lifestyle can improve pain-related self-efficacy and can indirectly influence health-related quality of life, perceived stress levels and the impact of pelvic pain on everyday life even in high pain-level groups.

**Acknowledgment**

This research was sponsored by the National Research, Development and Innovation Fund of Hungary (TKP-2021-EGA-10).

## A144 New ways of sport: The phenomenon of eSports and its impact on our lives

### Pál Novák

#### Faculty of Health Sciences, University of Pécs, Pécs, Hungary

##### **Correspondence:** Pál Novák (novak.pal@pte.hu)

*BMC Proceedings 2024*, **18(11)**: A144

**Background**

The author’s research has revealed that the phenomenon of e-sports has been poorly assessed from a social and academic standpoint, with significant issues, particularly for the most vulnerable actors in the esports industry, namely the esports players, not being adequately studied.

**Materials and methods**

The author employed a broad spectrum of literature analysis as one of the methods. Additionally, legal research was necessary, as e-sports encompasses various areas and levels of legislation, along with contractual relations between market actors and others involved in esports. Consequently, rules in these legal domains were analyzed to ascertain the classification of an e-sport player, such as whether it qualifies as labor or not.

**Results**

Every e-sport athlete starts as a recreational gamer and it is hard to find out who is an e-sport player and who is a gamer. The problem with that differentiation is that from a legal point of view e-sport players do not get different protection from the law as gamers. Also, it turned out that players often doing work related activities as e-sport athletes but they are not paid as workers and they do not have the same labor law protection either.

**Conclusions**

It would be important to find common, international agreements on the terms of e-sport industry and also clarify the misunderstood position of e-sport players because the e-athletes need more protection as they have now.

**Acknowledgment**

This research was sponsored by the National Research, Development and Innovation Fund of Hungary (TKP-2021-EGA-10).

## A145 Healthy Lifestyle Network Europe project

### Susana Franco^1^, Vera Simões^1^, Carla Chicau-Borrego^1^, John van Heel^2^, Simona Pajaujiene^3^, Aurimas Maciukas^4^, Farid Kempenaers^5^, Eric Vandenabeele^5^, Manel Valcarce-Torrente^6^, Sergio García Ortega^7^, Eva Rýzková^8^, Gabriela Luptakova^8^, Branislav Antala^8^, Jana Labudová^8^, Adriana Kaplánová^8^, Iris Španjol^9^, Bojana Harrison^10,11^, Darinka Korovljev^10,11^, Sergej M. Ostojić^10,11^

#### ^1^Sport Science School of Rio Maior – Santarém Polytechnic Institute, Rio Maior, Portugal; ^2^New Health Foundation, Weert, Netherlands; ^3^Lithuanian Sports University, Kaunas, Lithuania; ^4^Lithuanian Association of Health and Fitness Clubs, Kaunas, Lithuania; ^5^Fitness.be, Brussels, Belgium; ^6^Valencian International University, European Association Sport, Exercise and Health, Valence, Spain; ^7^European Association Sport, Exercise and Health, Valence, Spain; ^8^Faculty of Physical Education and Sports, Comenius University, Bratislava, Slovakia; ^9^European Network of Sport Education, Vienna, Austria; ^10^Faculty of Sport and Physical Education, University of Novi Sad, Novi Sad; ^11^Center for Health, Exercise and Sport Sciences, Belgrade, Serbia

##### **Correspondence:** Susana Franco (sfranco@esdrm.ipsantarem.pt)

*BMC Proceedings 2024*, **18(11)**: A145

**Background**

“Good health and well-being” are one of United Nations’ Sustainable Development Goals. It is proven that an unhealthy lifestyle can result in several non-communicable diseases. A combined intervention with healthy behaviors, such as physical activity, healthy eating, and a positive mindset is essential. Healthy literacy and lifestyle behavior awareness and changing should be promoted.

Healthy Lifestyle Network Europe (HLNE) is a 3 years European project (2023-2026), with 8 full partners, financed by ERASMUS Sport (no. 101133533) with 400.000€, following the previous European project New Health 2022 (NH2022; 2020-2022). Both aim to improve the healthy lifestyle of the European population, using physical activity, eating, and mindset.

**Case report**

To improve European citizens healthy lifestyles, during the NH2022 project, several materials were developed: a “New Health” website platform (www.new-health.eu) and a free App, where professionals, consumers, partners, and companies can register and do a Lifestyle Scan; a Serie of open 29 healthy lifestyle videos and an Open e-Book, about lifestyle coaching and behavior change. It was also developed EuropeActive’s Educational standards (EQF level 2) for Healthy Lifestyle Promoters (HLP) and a free HLP b-learning training course (divided into 3 parts: Exercise as Medicine; Food as Medicine; Brain as Medicine). HLNE will use the tools created in the project NH2022 and will: train more HLP, that will promote a healthy lifestyle to the population; develop the Healthy Lifestyle Coaches (HLC) qualification standards and create a training course for HLC who will coach consumers with health inequalities. The consortium will develop a European Healthy Lifestyle Education Institute.

**Conclusion**

It is expected, with these two projects improve the healthy lifestyle of citizens throughout Europe, and train professionals to become HLP or HLC. HLNE contributes to the quality of life of European citizens, and fight against obesity, stress and inactivity.

## A146 Role of sports in the psychological well-being of Hungarian adult population in COVID-19 pandemic

### Tamás Laczkó^1^, Csaba Melczer^1^, Kata Morvay Sey^1^, Miklós Stocker^2^

#### ^1^Faculty of Health Sciences, University of Pécs, Pécs, Hungary; ^2^Corvinus University of Budapest, Budapest, Hungary

##### **Correspondence:** Tamás Laczkó (joola.hu@gmail.com)

BMC Proceedings 2024, **18(11)**: A146

https://www.mdpi.com/1660-4601/20/1/660

## A147 The importance of health behavior for infertility-specific quality of life, with particular regard to physical activity

### Viktória Prémusz^1,2,3^, Eszter Skriba^4^, Gabor Szmatona^4^, Zoltan Tandor^4^, Kalman Kovacs^25^, and Akos Varnagy^2,3,5^

#### ^1^Institute of Physiotherapy and Sport Science, Faculty of Health Sciences, University of Pécs, Pécs, Hungary; ^2^National Laboratory on Human Reproduction, University of Pécs, Pécs, Hungary; ^3^János Szentágothai Research Center, University of Pécs, Pécs, Hungary; ^4^Doctoral School of Health Sciences, Faculty of Health Sciences, University of Pécs, Hungary; ^5^Department of Obstetrics and Gynecology, Medical School, University of Pécs, Pécs, Hungary

##### **Correspondence:** Viktória Prémusz (premusz.viktoria@pte.hu)

*BMC Proceedings 2024*, **18(11)**: A147

**Background**

The aim of the research was to examine the relationship between health, perceived stress, well-being and sports habits and physical activity among women undergoing assisted fertility treatment (ART).

**Materials and methods**

Cross-sectional study was conducted (81 patients, 31.79±4.64 years, BMI 25.51±5.32 kg/m2, child-wish 3.88±2.52 years) at the UP. Data were collected on well-being (WBI5), perceived (PSS), and infertility-related stress (COMPI-FPSS) and physical activity (GPAQ). Wilcoxon's signed rank test, Spearman's rho was performed with SPSS 28.0, p<0.05.

**Results**

40.74% played sports before, 20.99% during the treatments, half outdoors. 55.55% fulfilled the WHO recommendation. A positive correlation between playing sports during treatment and well-being (*R*=0.372, *p*<0.001), and a negative with perceived stress (*R*=-0.259, *p*=0.020) was found. Work-related MVPA was negatively correlated (*R*=-0.349, *p*=0.002), while recreational MVPA and somatic health were positively correlated (*R*=0.265, *p*=0.019). Based on the social subscale of the COMPI-FPSS, intensive recreational activity, refer to sports, showed an inverse relationship with infertility-related stress (*R*=-0.225, *p*=0.047).

**Conclusions**

We conclude that sport and recreational PA can have a positive effect on the health, well-being and perceived stress levels of women receiving ART. However, a randomized clinical trial could provide further evidence for our findings.

**Acknowledgment**

This research was sponsored by the National Research, Development and Innovation Fund of Hungary (NKFI FK-147404, ÚNKP-23-4-II-PTE-2061, TKP-2021-EGA-10).

## A148 Managing studies and sport: A qualitative investigation on student-athletes’ dual career pathways

### Giampaolo Santi^1^, Irene Lardschneider^1^, Ross Wadey^2^, Attilio Carraro^1^

#### ^1^Faculty of Education, Free University of Bozen-Bolzano, Bolzano, Italy; ^2^Faculty of Sport, Technology, and Health Sciences, St. Mary’s University Twickenham, London, United Kingdom

##### **Correspondence:** Giampaolo Santi (giamsanti@unibz.it)

*BMC Proceedings 2024*, **18(11)**: A148

**Background**

Dual Career programs for student-athletes have seen increasing interest in Europe in the last decade, with benefits for athletes’ sporting and academic careers, future employments, and personal lives. This Study explores student-athletes’ experiences and aims to identify: (1) different types of dual career pathways; (2) perceived challenges and opportunities in managing more pathways; and (3) competencies that participants felt to have acquired and transferred from sporting to academic contexts and vice versa.

**Materials and methods**

The Study consists of a qualitative investigation design (still ongoing), that involved seven student-athletes (2 females; age range: 19-44 years old) with past or current experience in managing two or more career pathways. Participants were interviewed online using a semi-structured grid and interviews were then transcribed verbatim. The first two authors also utilized the same interview grid for a self-reflection on their own experiences. Data were then analyzed through reflexive Thematic Analysis.

**Results**

Five different career pathways emerged: continuous dual career, continuous student-discontinuous athlete, discontinuous student-continuous athlete, discontinuous student-discontinuous athlete, and dual career plus (e.g., participants that also had part-time jobs). Challenges and opportunities were classified according to a multilevel approach from the macro- (environmental factors, e.g., COVID-19 pandemic), to the meso- (supporting or obstructive behaviors experienced in organizations or in the family), to the micro-level (e.g., personal strengths and weaknesses in managing adversity). Focusing on their youth experiences, participants brainstormed several skills that they had acquired in sport and transferred to educational contexts, or the other way around, including time management, goal setting, ability to dialogue with adults to conciliate commitments.

**Conclusions**

This investigation highlights multiple career pathways that are possible for people who pursue both academic and sport achievements (and maybe other life goals) and provides information on the possible benefits of these choices. Findings may advice traditional and online universities on which services best fit student-athletes’ needs.

## A149 Effectiveness of set-plays depending on the area of the field and the time period of the match in women's soccer

### Katerina Papadimitriou, Sofia Papaioannou, Katerina Daskalaki, Anestis Giannakopoulos, Xanthi Konstantinidou, Ourania Matsouka

#### Department of Physical Education and Sport Science, Democritus University of Thrace, Komotini, Greece

##### **Correspondence:** Katerina Papadimitriou (kpapadim@phyed.duth.gr)

*BMC Proceedings 2024*, **18(11)**: A149

**Background**

In soccer one of the factors that affect performance is static phases. Research has shown that 1/3 of goals in football matches are scored after the execution of the static phases. The purpose of this research was to study the effectiveness of static phases in high level women's soccer and how this was affected by the area of the field and the time period of the match.

**Materials and methods**

The sample consisted of 61 matches from the Women's Champions League that took place in the 2021-2022 season. A total of 703 set-plays were observed. For the games analysis, the PC program Sportscout was used. Crosstabs and the significance value Chi-square test (*p*<.05) was used for data analysis. The parameters of observation: a) type of set-plays, b) effectiveness, c) zone of the field, c) time period of the mach.

**Results**

The results showed that most of set-plays were corners, indirect free kicks, direct free kicks and penalties. Most goals were scored from the right attacking area and the penalty area. Also, most of the goal opportunities were made from the right as well as from the left attacking area. On the contrary, unsuccessful set-plays were taken from the left and right areas, while the loss of possession of the ball occurred behind the center circle of the field

**Conclusions**

The effectiveness of the set-plays depended significantly only on the field area. This information can be used by the coaches for the development of women's football in lower categories.

## A150 Heat alert and heat-related mortality in Hungary in the period 2011-2023

### Anna Paldy^1^, Dragan Milošević^2,3,4^, Marijana Ranisavljev^4,5^, Nikola Todorović^4,5^, Zoltán Ábrám^6^, Loránd Ferencz^6^, Valentin Nădăsan^6^

#### ^1^National Center for Public Health and Pharmacy, Budapest, Hungary; ^2^Meteorology and Air Quality Section, Wageningen University & Research, Wageningen, Netherlands; ^3^Hydrology and Environmental Hydraulics Section, Wageningen University & Research, Wageningen, Netherlands; ^4^Center for Health, Exercise and Sport Sciences, Belgrade, Serbia; ^5^Faculty of Sport and Physical Education University of Novi Sad, Novi Sad, Serbia; ^6^Department of Hygiene, George Emil Palade University of Medicine, Pharmacy, Science, and Technology of Targu Mures, Targu Mures, Romania

##### **Correspondence:** Anna Paldy (paldy.anna@nnk.gov.hu)

*BMC Proceedings 2024*, **18(11)**: A150

**Background**

The impact of heatwaves was recognized across Europe in 2003 and although heat alerts and preventive measures have been introduced in many countries, attributable mortality is still between 11% and 35%. in Europe. We aimed to analyze the factors influencing excess mortality during heatwaves in Hungary.

**Materials and methods**

To develop a heat-health alert system (HHAS), the relationship between meteorological parameters obtained from the National Meteorological Service and mortality data provided by the Central Statistical Office was assessed using time series analysis based on daily data series from Budapest for the period 1970-2000. For the assessment of HHAS, daily mortality data were obtained from the Civil Registry for the period 2011-2023. Excess mortality is defined as the difference between the average mortality on summer days with a mean temperature below 25 °C and on days above this temperature threshold. Additionally, data on flu-like mortality were obtained from the Euromomo network.

**Results**

A 5°C increase in average daily temperature above 25°C increased all-cause mortality by 10%. in Budapest. Based on this, a 3-level HHAS was developed in 2005. The characteristics of excess mortality during heat alerts and its association with the severity of influenza epidemics are presented. The excess mortality was 27%, 36%, and 23% during the first summer heatwave in 2012, 2013, and 2014, years characterized by mild influenza epidemics. In years when excess mortality during influenza epidemics was high, mortality during August heatwaves was relatively high (15-20%). Furthermore, we found a slight downward trend in excess deaths during heat alerts between 2011 and 2023.

**Conclusions**

Despite warmer summers since 2015, excess mortality has been decreasing since the establishment and application of HHAS in Hungary. To further reduce the impact of heat, the education of healthcare professionals and communication should be strengthened as key elements of heat adaptation.

## A151 Impact of summer heat on mortality and hospital admissions in the cities of Serbia

### Dragan Milošević^1,2,3^, Anna Paldy^4^, Marijana Ranisavljev^3^, Nikola Todorović^3^, Frances Shiely^5^, Séamus Mc Monagle^5^

#### ^1^Meteorology and Air Quality Section, Wageningen University & Research, Wageningen, Netherlands; ^2^Hydrology and Environmental Hydraulics Section, Wageningen University & Research, Wageningen, Netherlands; ^3^Center for Health, Exercise and Sport Sciences, Belgrade, Serbia; ^4^National Center for Public Health and Pharmacy, Budapest, Hungary; ^5^HRB Clinical Research Facility and School of Public Health, University College Cork, Cork, Ireland

##### **Correspondence:** Dragan Milošević (dragan.milosevic@dgt.uns.ac.rs)

*BMC Proceedings 2024*, **18(11)**: A151

**Background**

Studies have shown that the impact of heat on mortality and hospital admissions can be significant, especially during heatwaves. High temperatures can directly lead to health problems such as heat exhaustion and dehydration, increasing mortality rates, especially among vulnerable populations, but studies have also shown the significant impact of heat stress in sports and exercise. In Serbia, like many other countries, heatwaves can have a significant impact on public health, including increased mortality rates and hospital admissions.

**Materials and methods**

The effects of summer air temperatures on mortality in Serbia in the period 2001-2015 were assessed. Air temperature data for Belgrade, Novi Sad, Niš, Loznica, and Vranje are obtained from the Official Meteorological Yearbooks. For each location, mortality data were obtained from the Statistical Office of the Republic of Serbia. Data on daily hospital admissions and summer air temperatures in Novi Sad were obtained from the database of the Institute of Public Health of Vojvodina for the period 2016-2017.

**Results**

The results showed an increase in the average daily number of deaths in Serbian cities (Belgrade, Novi Sad, Niš, Loznica, and Vranje) during extreme temperature periods in summer. A 1 °C increase in air temperature was associated with a 1.7% increase in crude death rates for Belgrade, Novi Sad, and Niš, and 2.0% for Loznica. Regarding hospital admissions, the data for Novi Sad showed that when the daily temperature range is between 10 °C and 14 °C, there is a significant increase in hospital admissions of people aged 65 or more.

**Conclusions**

Public health interventions such as heat advisories and cooling centers should be developed to mitigate the impacts of heat on health and well-being, especially in the context of climate change and urbanization that will increase the frequency and intensity of heatwaves in the coming decades.

## A152 Training medical and public health students of the future: open online CLIMATEMED program

### Frances Shiely^1^, Séamus Mc Monagle^1^, János Girán^2^, Ágnes Szenczi^2^, Gergely Márovics^2^, Paul Edit^3^, Zsuzsanna Máté^3^, Zoltán Abrám^4^, Loránd Ferencz^4^, Valentin Nădăsan^4^, Darinka Korovljev^5,6^, and Sergej M. Ostojić^5,6^

#### ^1^HRB Clinical Research Facility and School of Public Health, University College Cork, Cork, Ireland; ^2^Medical School, Department of Public Health, University of Pécs, Hungary; ^3^Albert Szent-Györgyi Medical School, Department of Public Health, University of Szeged, Szeged, Hungary; ^4^Department of Hygiene, George Emil Palade University of Medicine, Pharmacy, Science, and Technology of Targu Mures, Romania; ^5^Center for Health, Exercise and Sport Sciences, Belgrade, Serbia; ^6^Faculty of Sport and Physical Education, University of Novi Sad, Novi Sad, Serbia

##### **Correspondence:** Frances Shiely (f.shiely@ucc.ie)

*BMC Proceedings 2024*, **18(11)**: A152

**Background**

The urgency of addressing the profound health impacts of climate change has become increasingly apparent, with projections indicating significant risks to human populations and ecosystems in the coming decade. The World Health Organization predicts a staggering rise in additional deaths due to climate change-related health issues, highlighting the critical need for immediate action. However, medical education on climate-related health risks remains inadequate, with only a fraction of medical schools worldwide offering dedicated courses on the subject. Recognizing this gap, the CLIMATEMED project aims to revolutionize medical and public health education by developing comprehensive curricula that integrate climate change and health topics, with a focus on preventative measures.

**Materials and methods**

The consortium of five European universities has developed a curriculum comprising twelve lessons for both face-to-face and online learning, along with related pedagogical training materials, all available on an open-access e-learning platform.

**Results**

The project targets undergraduate medical and public health students, practicing medical professionals, and academic staff, seeking to emphasize the relevance of climate change-related health challenges and promote their integration into as many European Union medical and public health schools as possible. Outputs include: (1) semester-long lecture series and learning materials for students, emphasizing preventative measures in English, Hungarian, Romanian and Serbian languages; (2) training programs and materials for academic staff; (3) a teachers’ guide for curriculum integration; (4) postgraduate training material for medical doctors, including a curriculum and exercises for 90-minute lectures/seminars.

**Conclusions**

CLIMATEMED addresses the gap between the increasing demand for healthcare due to climate change and the current knowledge taught in medical and public health schools. The project's outputs will assist participating universities and others in the EU and beyond to integrate climate change education into their curricula. Furthermore, its pedagogical approach enhances institutions' capacity for effective digital education delivery.

## A153 Voices from the classroom: Students and academics' opinions on climate change and health in medical and health education

### János Girán^1^, Ágnes Szenczi^1^, Gergely Márovics^1^, Frances Shiely^2^, Séamus Mc Monagle^2^, Zoltán Ábrám^3^, Ferencz Loránd^3^, Valentin Nădăsan^3^, Dragan Milošević^4,5,6^, Bojana Harrison^6,7^, and Valdemar Štajer^6,7^

#### ^1^Medical School, Department of Public Health, University of Pécs, Hungary; ^2^Clinical Research Facility and School of Public Health, University College Cork, Cork, Ireland; ^3^George Emil Palade University of Medicine, Pharmacy, Science, and Technology of Targu Mures, Department of Hygiene, Romania; ^4^Meteorology and Air Quality Section, Wageningen University & Research, Wageningen, Netherlands; ^5^Hydrology and Environmental Hydraulics Section, Wageningen University Camp; Research, Wageningen, Netherlands; ^6^Center for Health, Exercise and Sport Sciences, Belgrade, Serbia; ^7^Faculty of Sport and Physical Education, University of Novi Sad, Novi Sad, Serbia

##### **Correspondence:** János Girán (janos.giran@aok.pte.hu)

*BMC Proceedings 2024*, **18(11)**: A153

**Background**

The CLIMATEMED Project aims to support medical and health education by developing learning materials that focus on climate change’s health aspects (CcHA). To ensure that the learning materials can be used effectively, opinions were collected from relevant target groups, namely students and academics.

**Materials and methods**

The opinions were collected using the World Café method, which encourages participants to share their insights on the topic under study through small group discussions and to develop ideas synergistically. The data collection involved 119 students and 52 academics from medical and public health schools in four universities in four countries (Hungary, Ireland, Romania, and Serbia). The questions focused on the presence of CcHA in current education, the need to integrate CcHA into curricula and teaching materials, and how the topics should be integrated into education.

**Results**

The students highlight the inadequate presence of CcHA in current education and argue for its integration because of its paramount importance. Academics also recognize the relevance of CcHA, but note time constraints for addressing other crucial topics. Students would either have CcHA-related knowledge in the framework of a stand-alone subject or if the relevant associations were highlighted during the education of different subjects. The academics propose enhancing effectiveness by integrating climate change aspects into the core subject matter and that some closely related subjects (hygiene, public health) could devote some whole lectures to CcHA. This could be complemented by an optional stand-alone CcHA e-learning module.

**Conclusions**

According to the opinions collected, there is strong support from students but also a need from academics to address the CcHA-related deficiencies in the current curriculum of medical and health education. Consequently, the outcomes of the CLIMATEMED Project can effectively address the articulated needs of students and academics.

## A154 Comparison of the effects of voluntary physical activity on mitochondrial function and lipid breakdown in the liver of aged rats with different running capacities

### Soroosh Mozaffaritabar^1^, Smaragda Giannopoulou^2^, Lei Zhou^1^, Zsolt Radak^1^

#### ^1^Research Institute of Molecular Exercise Science, Hungarian University of Sports Science, Budapest, Hungary; ^2^Department of Pharmacognosy, University of Vienna, Vienna, Austria

##### **Correspondence:** Soroosh Mozaffaritabar (sorooshmozaffari@gmail.com)

*BMC Proceedings 2024*, **18(11)**: A154

**Background**

Aging induces physical decline, affecting tissues like liver. Aging leads to substantial degenerative changes, increasing susceptibility to oxidative stress. Mitochondrial dysfunction, a hallmark of cellular aging, results in oxidative damage, affecting liver's redox balance. Accumulated liver fat requires lifestyle changes, including healthy diet and exercise. Physical activity promotes lipid metabolism, mitigating liver disorder risks. Exercise reinforces the liver's antioxidant defense, improving mitochondrial activity.

**Materials and methods**

A six-month study, supported by the Hungarian New National Excellence Program (ID no. 23-3-II-MTSE-5), explored voluntary exercise effects on 23 aged rats (HCR and LCR groups: LCR-SED, LCR-EX, HCR-SED, HCR-EX). Under standard conditions, rats underwent post-exercise sacrifice, and livers were extracted. Biochemical analysis used Western blotting targeting lipases associated with mitochondrial function and lipid breakdown. Statistical analysis employed graph pad prism software (*p < 0.05).

**Results**

Analysis revealed significant increases in response to voluntary exercise in both types, indicating distinct molecular reactions. HCR rats exhibited elevated levels of ATGL, HSL, Sirtuin 3 (Sirt3), Citrate Synthase (CS), and Lon Peptidase 1 (LONP1). Conversely, LCR rats showed increased ATGL, Sirt3, Sirtuin 1 (Sirt1), CS, and LONP1 levels.

**Conclusions**

Our findings deepen our understanding of voluntary exercise effects on hepatic markers in aging rats, offering insights for therapeutic development against age-related hepatic lipid problems. Alterations in lipid metabolism (ATGL, HSL), mitochondrial function (CS, LONP1), and key regulatory components (Sirt3, Sirt1 emphasize exercise's potential preventive and therapeutic role in age-related liver disorders.

## A155 Understanding the treat of physical inactivity to increase adherence to physical activity: Insights from two decades of research at the Bedrest Centre in Koper

### Rado Pišot^1^, Boštjan Šimunič^1,2^, Uroš Marušič^1,2^, Mladen Gasparini^3^, Gianni Biolo^4^, Marco Narici^1,5^

#### ^1^Science and Research Center Koper, Institute for Kinesiology Research, Koper, Slovenia; ^2^Department of health sciences, Alma Mater Europaea – ECM, Maribor, Slovenia; ^3^General Hospital of Izola, Izola, Slovenia; ^4^Clinical Department of Medical, Surgical and Health Sciences, Trieste, Italy; ^5^Department of Biomedical Sciences, University of Padova, Padua, Italy

##### **Correspondence:** Rado Pišot (rado.pisot@zrs-kp.si)

*BMC Proceedings 2024*, **18(11)**: A155

**Background**

Microgravity poses numerous health challenges for astronauts, necessitating the development of effective physical exercise modalities to counteract its adverse effects on their well-being. However, the significance of physical activity (PA) extends beyond space missions, playing a crucial role in maintaining optimal health on Earth. PA serves as a catalyst for physical fitness, enhanced work efficiency, fortified immune system resilience, and the preservation of psychophysical balance. While PA has always been integral to human evolution, our evolving relationship with gravity is transitioning from opposition to acquiescence (or countering and acceptance). Abrupt periods of physical inactivity (PI) can expedite the deterioration of health, particularly in individuals already compromised by the natural aging process. The negative repercussions of hospitalization on the health of older individuals may be attributed in part to the challenges arising from the primary illness and the disruptions in daily habits, leading to a sudden reduction in PA.

**Materials and methods**

The experimental bed rest protocol, offers a pragmatic and relevant model for studying the detrimental consequences of inactivity and muscle wasting in bedridden (physical inactive) individuals.

**Results**

Over the past two decades, the Bedrest Centre Koper, department of Institute for Kinesiology Research at ZRS Koper, Slovenia, has conducted seven BED-REST studies in collaboration with various partners, fostering fruitful partnerships with the Izola General Hospital and the Valdoltra Orthopedic Hospital. Key observations and specific variations observed among participants, their age, bed-rest durations, modalities, and countermeasures employed in these studies will be presented. Approaches to research and study changes will be presented, as well as mechanisms of decline in monitored functional abilities, the understanding of which provides insights into similar changes in the process of ageing or sedentarism.

**Conclusions**

The findings contribute to the understanding of the multifaceted impacts of microgravity simulation on Earth and underscore the importance of mitigating the adverse effects of prolonged inactivity for overall people's health and well-being

## A156 Is walking associated with better self-rated health?

### Ivana Radić^1,2^, Sonja Čanković^1,2^, Sanja Harhaji^2^, Nataša Dragnić^2^, Vesna Mijatović Jovanović^1,2^, Zorana Ostojić^2^

#### ^1^Faculty of Medicine, University of Novi Sad, Novi Sad, Serbia; ^2^Institute of Public Health of Vojvodina, Novi Sad, Serbia

##### **Correspondence:** Ivana Radić (ivana.radic@mf.uns.ac.rs)

*BMC Proceedings 2024*, **18(11)**: A156

**Background**

Walking has a positive impact both on physical and mental health. As it is accessible behavior for most of the people it has a great potential for increasing population physical activity levels. The aim of this study is to assess the association between walking and self-rated health.

**Materials and methods**

The study is a part of the National Health Survey, a cross-sectional study conducted in the year 2019. Instruments of the survey were questionnaires designed in line with the European Health Interview Survey and physical activity was assessed using the International Physical Activity Questionnaire. We used data for 6211 women and 6544 men aged 18 and over. Multivariable logistic regression was used to analyze the association between walking for transportation and self-rated health (adjustment was made for gender, age, educational level, marital status, work-related physical activity, cycling for transportation and leisure time physical activity).

**Results**

In Serbia, every second adult walks for transportation more than 300 minutes weekly (56.2%) and 7.1% never does this kind of activity. Among people who walk 300 minutes or more weekly 72.3% report their health as good or very good, while this proportion significantly decreases to 38.8% among those who never walk. According to logistic regression analysis, longer walking time was associated with better self-rated health. Adults who walked 300 minutes or more weekly (OR 1.98, 95%CI 1.65-2.37) or 150 to 299 minutes weekly (OR 1.87, 95%CI 1.41-2.50) had almost two times higher odds to assess their health as good than those who never walk for transportation.

**Conclusions**

The findings of this study suggest that public health interventions that promote walking, as easily achievable form of physical activity, may improve population health status.

## A157 Physical activity and depressive symptoms in adolescents in Vojvodina

### Sonja Čanković^1,2^, Vesna Petrović^3^, Dušan Čanković^1,2^, Ivana Radić^1,2^, Vesna Mijatović Jovanović^1,2^

#### ^1^Faculty of Medicine, University of Novi Sad, Novi Sad, Serbia; ^2^Institute of Public Health of Vojvodina, Novi Sad, Serbia; ^3^Primary Health Care Centre “Dr. Milorad Mika Pavlović”, Inđija, Serbia

##### **Correspondence:** Sonja Čanković (sonja.cankovic@mf.uns.ac.rs)

*BMC Proceedings 2024*, **18(11)**: A157

**Background**

Physical activity is one of the factors that was identified as beneficial to the mental health status. The main objective of this study is to determine the prevalence of physical inactivity and the presence of depressive symptoms among adolescents and to examine whether physical inactivity is the risk factor for the development of depressive symptoms.

**Materials and methods**

The sample consisted of 986 high school students (47.4% girls and 52.6% boys) from ten government high schools in all seven districts of the province of Vojvodina. The International Physical Activity Questionnaire (IPAQ) was used as a self-report measure of physical activity using the last week as a reference period, and the Kutcher Adolescents Depression Scale (KADS-6) as a screening test for symptoms of depression in young individuals (KADS-6 score ≥6). Socio-demographic data was collected using a questionnaire developed for this study. Logistic regression analyses were used to explore the association between the presence of depressive symptoms and physical inactivity.

**Results**

Analysis has shown that 27.9% of girls and 14.7% of boys (p<0.001) could have depression according to the KADS-6 score, and the prevalence of physical inactivity among adolescents was 29.3%, significantly more among girls (33.4% vs. 25.6%; p<0.001). Adjusted models revealed that inactive boys had almost two times higher risk of having depressive symptoms compared to those who were moderate or vigorous physically active (OR = 1.82; 95% CI: 1.04–3.18). Among girls, physical inactivity was not a predictor of depressive symptomatology.

**Conclusions**

The study revealed a high prevalence of physical inactivity and depressive symptoms among adolescents in Vojvodina. Enhancing physical activity levels in adolescents, considering the specific characteristics of this population group, is of great importance to prevent mental health disorders.

## A158 CLIMBWISE project: Children's holistic development through sport climbing

### Ahac Istenič, Luka Šlosar, Uroš Marušič

#### Science and Research Center Koper, Institute for Kinesiology Research, Koper, Slovenia

##### **Correspondence:** Ahac Istenič (ahac.istenic@zrs-kp.si)

*BMC Proceedings 2024*, **18(11)**: A158

**Background**

The rising prevalence of sedentary lifestyles in children raises concerns about adverse health outcomes. The CLIMBWISE project aims to address this issue by promoting sport climbing as a holistic solution to engage children in physical activity, enhancing overall health, motor skills, and psychological well-being. The project's initiative focuses on making sport climbing more accessible, improving coaching practices, and opening new horizons for using motion analysis in sport climbing practices.

**Materials and methods**

The guidelines will be crafted by analyzing current coaching practices and conducting a comprehensive literature review. To tackle personnel shortages and enhance inclusivity, coaches will undergo comprehensive training, starting with theoretical instruction. Subsequently, they will seamlessly transition this knowledge into practice by actively leading climbing courses. Additionally, the project aims to incorporate motion analysis, utilizing highly accurate, real-time tracking systems and motion analysis based on simple video recordings.

**Results**

The project anticipates three main outcomes: increased participation of children in climbing activities, educated coaches, and the implementation of motion analysis into climbing practices. Sport climbing will contribute to well-rounded growth of engaged children. The dynamic interplay of strength, continuous motion, flexibility, coordination, and balance underscores sport climbing's transformative role in facilitating comprehensive development. Moreover, the initiation of motion analysis in this project marks a significant milestone, providing a fantastic opportunity to expand its integration into sport climbing training and research.

**Conclusions**

Project targets sedentary lifestyles in children by promoting sport climbing for holistic well-being. Crafted guidelines, coach education, and motion analysis integration anticipate increased child participation, educated coaches, marking a pivotal step in advancing sport climbing practices. This initiative not only addresses urgent health concerns but also opens a door to new horizons in sport climbing research, enhancing the number of children involved and fostering lifelong fitness habits and comprehensive child development.

## A159 Use of mobile applications in estimating the speed of serve in tennis (preliminary research)

### Fran Masnjak, Petar Barbaros, Zlatan Bilić

#### Faculty of Kinesiology, University of Zagreb, Zagreb, Croatia

##### **Correspondence:** Petar Barbaros (petar.barbaros@kif.unizg.hr)

*BMC Proceedings 2024*, **18(11)**: A159

**Background**

The use of mobile applications in modern tennis is on the rise. There are many mobile apps on the market appearing to measure the speed of the serve. However, the accuracy and practicality of these applications have not been adequately demonstrated or objectively assessed.

**Materials and methods**

This study aims to determine the validity of four mobile applications (Speedup Tennis, Tennis Serve Tracker, Speed Gun for Tennis, Velocity by Athla) that are available on the iOS 15.0 or earlier app store. The serve speeds recorded by these applications are compared to the results obtained from the Stalker ATSII radar. The sample group consists of 20 tennis players, with an average age of 22±1.12 years. The variables measured include the serve speed recorded by each application and the serve speed measured by the Stalker ATSII radar.

**Results**

The findings reveal that the Tennis Serve Tracker app exhibits the strongest correlation with the radar (ICC=0.88), followed by Speed Gun for Tennis (ICC=0.63), Speedup Tennis (ICC=0.20), and Velocity by Athla (ICC=0.01) displaying the weakest correlation with the radar.

**Conclusions**

The practicality of mobile applications lies in their affordability and simplicity of use for tennis players and coaches, making them a valuable supplementary tool for optimal training planning and programming.

## A160 Correlation between speed and vertical jump performance in professional football players

### Slavko Molnar, Dejan Javorac

#### Faculty of Sport and Physical Education, University of Novi Sad, Novi Sad, Serbia

##### **Correspondence:** Dejan Javorac (dejan.javorac@fsfvns.edu.rs)

*BMC Proceedings 2024*, **18(11)**: A160

**Background**

Football as a sport provides young people with the physical ability and movement habits necessary to perform various tasks in training and contribute to the game. Speed and vertical jumping are critical motor abilities in training in almost all team sports. This research analyzes the correlation between speed and vertical jump in professional football players of the First League of Serbia.

**Materials and methods**

The sample of respondents consisted of 26 professional football players (24.85±5.63 yrs.) from the First League of Serbia. Sprinting abilities were tested with a Witty system device (micro gate) by running 5m, 10m, and 20m, while explosive abilities were tested on a tensiometric platform. Initially, the normality of the data distribution was established using the Kolmogorov Spearman test and the calculation of basic descriptive statistics. The Pearson correlation coefficient was used to determine the correlations between football players' speed and vertical jump (p≤0.05).

**Results**

The results showed a statistically significant correlation between speed and vertical jumping in professional football players. A correlation was established between the tests of Vertical jump and the speed at 20m (*r*=-0.44, *p*=0.05). Then, the correlation between the variables Squat jump and speed at 5m (*r*=-0.49, *p*=0.03) and speed at 20m (*r*=-0.56, *p*=0.01). Finally, a correlation was found between the Countermovement Jump and the speed at 20 m (*r*=-0.56, *p*=0.01).

**Conclusions**

Speed and vertical jumping are related abilities in football players. Coaches should better understand these motor abilities and include them in the training process in an optimized way, thus developing them and improving other sports performances in football.

## A161 Age-related differences in single- and dual-task walking after 10 days of bed rest

### Uroš Marušič, Manca Peskar, Luka Šlosar, Rado Pišot

#### Science and Research Center Koper, Institute for Kinesiology Research, Koper, Slovenia

##### **Correspondence:** Uroš Marušič (uros.marusic@zrs-kp.si)

*BMC Proceedings 2024*, **18(11)**: A161

**Background**

Prolonged bed rest can impair mobility and balance, leading to muscle atrophy, reduced cardiovascular fitness and neuromuscular changes. Single-task walking measures basic mobility, while dual-task walking assesses multitasking and gait stability in real-life situations. The aim of this study was to investigate the effects of prolonged bed rest on single- and dual-task walking in young and older individuals exposed to 10 days of bed rest.

**Materials and methods**

A group of eight older people (mean age = 68 years) and eight younger people (mean age = 22 years) underwent 10 days of bed rest. Before and after bed rest, the participants' gait speed was measured under self-selected conditions in both single and dual-task conditions. The dual-task condition involved walking while engaging in a texting task, with accuracy and number of letters typed monitored. Gait parameters were measured using both the Optogait and an Inertial Measurement Unit - based system. Gait speed served as the primary outcome measure, which was analyzed using repeated measures analysis of variance to examine the effects of bed rest on gait performance in both age groups.

**Results**

A significant time effect was observed in the single-task condition (*p* = 0.039), with post-hoc analyses showing a tendency towards reduced gait speed in older adults (*p* = 0.091) from pre- to post- bed rest. Similarly in the dual-task condition, only older adults showed a decrease in gait speed from pre- to post- bed rest (*p* = 0.008).

**Conclusions**

The study shows that prolonged bed rest leads to a significant deterioration in both single and dual-task walking ability, which is particularly evident in older adults. This emphasizes the importance of interventions to mitigate these effects and maintain functional mobility in older adults exposed to prolonged bed rest.

## A162 Cognitively enhanced physical activity and executive function in preschool children: A systematic review

### Nemanja Lakićević^1^, Marko Manojlović^2^, Ambra Gentile^3^, Sergey Leonov^1^, Aleksander Veraksa^1^, Patrik Drid^2^

#### ^1^Department of Psychology of Education and Pedagogy, Moscow State University Lomonosov, Russia; ^2^Faculty of Sport and Physical Education, University of Novi Sad, Serbia; ^3^Sport and Exercise Sciences Research Unit, University of Palermo, Italy

##### **Correspondence:** Nemanja Lakićević (lakinem89@gmail.com)

*BMC Proceedings 2024*, **18(11)**: A162

**Background**

Preschool children spend majority of their time in kindergartens in sedentary pursuits. Low levels of physical activity (PA) combined with cognitively non-stimulating environment can impede executive function (EF) which is deemed a as crucial factor for optimal academic achievements later on. Time efficient Interventions that are combing PA and EF are therefore gaining increasing attention.

**Materials and methods**

We sought to perform systematic review to identify all studies that conducted PA intervention jointly with cognitive tasks in preschool children (3-5 years old) and measured EF as the main outcome. Only randomized control trials published in English were deemed appropriate to be included.

**Results**

Seven studies met the inclusion criteria and had a total of 760 participants. Our findings indicated inconclusive evidence with some studies showing improvement in working memory and inhibition, and no changes in cognitive flexibility. Intensity of PA and difficulty of cognitive task seem to be the determining factors in developing EF in preschool children.

**Conclusions**

Cognitively enhanced PA has the potential to improve EF in preschool children but PA sessions need to carried out by teachers who have been trained properly and can adjust the intervention in accordance to individual capabilities of children and setting where the PA is conducted.

